# Single-cell genomics reveal low recombination frequencies in freshwater bacteria of the SAR11 clade

**DOI:** 10.1186/gb-2013-14-11-r130

**Published:** 2013-11-28

**Authors:** Katarzyna Zaremba-Niedzwiedzka, Johan Viklund, Weizhou Zhao, Jennifer Ast, Alexander Sczyrba, Tanja Woyke, Katherina McMahon, Stefan Bertilsson, Ramunas Stepanauskas, Siv G E Andersson

**Affiliations:** 1 Department of Molecular Evolution and Science for Life Laboratory, Uppsala University, 751 24 Uppsala, Sweden; 2 Computational Metagenomics, Center for Biotechnology, Bielefeld University, 335 01 Bielefeld, Germany; 3 DOE Joint Genome Institute, Walnut Creek, CA 94598, USA; 4 Departments of Civil and Environmental Engineering and Bacteriology, University of Wisconsin, Madison, WI 53706-1691, USA; 5 Department of Ecology and Genetics, Limnology and Science for Life Laboratory, Uppsala University, 752 36 Uppsala, Sweden; 6 Bigelow Laboratory for Ocean Sciences, East Boothbay, ME 04544-0380, USA

## Abstract

**Background:**

The SAR11 group of Alphaproteobacteria is highly abundant in the oceans. It contains a recently diverged freshwater clade, which offers the opportunity to compare adaptations to salt- and freshwaters in a monophyletic bacterial group. However, there are no cultivated members of the freshwater SAR11 group and no genomes have been sequenced yet.

**Results:**

We isolated ten single SAR11 cells from three freshwater lakes and sequenced and assembled their genomes. A phylogeny based on 57 proteins indicates that the cells are organized into distinct microclusters. We show that the freshwater genomes have evolved primarily by the accumulation of nucleotide substitutions and that they have among the lowest ratio of recombination to mutation estimated for bacteria. In contrast, members of the marine SAR11 clade have one of the highest ratios. Additional metagenome reads from six lakes confirm low recombination frequencies for the genome overall and reveal lake-specific variations in microcluster abundances. We identify hypervariable regions with gene contents broadly similar to those in the hypervariable regions of the marine isolates, containing genes putatively coding for cell surface molecules.

**Conclusions:**

We conclude that recombination rates differ dramatically in phylogenetic sister groups of the SAR11 clade adapted to freshwater and marine ecosystems. The results suggest that the transition from marine to freshwater systems has purged diversity and resulted in reduced opportunities for recombination with divergent members of the clade. The low recombination frequencies of the LD12 clade resemble the low genetic divergence of host-restricted pathogens that have recently shifted to a new host.

## Background

Microbial genomes change in gene content by duplications, deletions and horizontal gene transfers, and in sequence by nucleotide substitutions and homologous recombination. The relative contribution of recombination to sequence divergence has been determined in a wide range of microorganisms [1]. Much of the results have been inferred from the analyses of rRNA genes and protein-coding genes used for multilocus sequence typing. Comparisons of such data between species have shown that the ratio at which a nucleotide becomes substituted as a result of recombination versus mutations (*r/m*) ranges from very low (<1) to extremely high (>10) [1]. Because of these differences, some bacterial populations can be sorted into discrete subclusters, whereas others are best described as a continuum of sequence variants.

Bacteria with a clonal population structure, low levels of genetic diversity and low *r/m* ratios tend to be host-adapted. They have often experienced a population bottleneck associated with the adaptation to a single host and are referred to as ‘genetically monomorphic species’ [2]. On the other extreme are environmental bacteria, such as the SAR11 group of Alphaproteobacteria, which can make up to 30% of the total marine bacterioplankton in the upper surface waters of the oceans [3,4]. The *r/m* ratio for SAR11 isolates has been estimated to 63 [1,5], which is one of the highest *r/m* ratios recorded for bacteria.

It has been hypothesized that a high recombination frequency in the marine SAR11 bacteria is the result of selection to ensure variability in the phage receptor protein sequences [1,5]. Indeed, highly abundant SAR11-specific pelagiphages were recently identified in the oceans [6] and the presence of hypervariable regions (HVRs) in the marine SAR11 genomes that code for the biogenesis of outer membrane components [7] provides indirect support for co-evolutionary interactions with pelagiphages. The SAR11 bacteria are characterized by having very small genome sizes, in the 1.4 to 1.6 Mb range, and extremely small cell volumes, which is suggested to result from selection to enhance the surface to volume ratio [7-9].

The SAR11 group of aquatic bacteria contains a freshwater clade that has diverged relatively recently from the marine groups [7]. The freshwater clade of the SAR11 group [10-12] was first identified in an Arctic Toolik lake, and is referred to as LD12 [13,14]. Based on ribosomal RNA phylogenies, LD12 is currently classified as subtype IIIb of the SAR11 clade [7]. Isotope trace studies have provided some information on organic substrate use [15] and single cell surveys indicate that LD12 constitutes 1% to 21% of freshwater bacterioplankton [15]. The transition from marine to freshwater ecosystems was a unique event, which is thought to have happened only once in the evolutionary history of the SAR11 clade [12,16]. Because of their close relatedness, freshwater and saltwater SAR11 lineages enable comparative studies of the population dynamics of bacteria that have shifted their ecological niche from the open oceans to physically constrained lake ecosystems. However, such studies have been hampered because there are no cultivation methods available for LD12, and thus, no genomes have yet been sequenced.

Single cell genomics is emerging as a new method to study the genomes of uncultivated microorganisms [17,18]. Here, we report a comparative analysis of ten single-cell genomes from LD12 along with recruited metagenome reads from six lakes. The results indicate that the transition to freshwater has been associated with dramatic changes in the population dynamics of the SAR11 group of bacteria.

## Results and discussion

### Single cell genome sequencing of LD12

To gain insight into the LD12 genomes, fluorescence-activated cell sorting was used to isolate single bacterial cells from lakes Sparkling, Damariscotta and Mendota [19]. Their genomic DNA was amplified by the multiple displacement amplification method and cells putatively belonging to the freshwater group of the SAR11 clade were identified through sequencing of partial 16S rRNA genes using universal bacterial primers. Ten unique LD12 single amplified genomes (SAGs) were selected for genomic sequencing (Table 1; Table S1 in Additional file 1). Genome assemblies ranged from 627 to 925 kb, with the exception of N17, which assembled into a genome sequence of only 328 kb. Three assemblies contained scaffolds larger than 100 kb and all but the N17 SAG contained two or more scaffolds larger than 50 kb.

**Table 1:** Details of the assembly and annotations

**SAG**	**Size**	**Scaffolds**	**Genes**
D10	925,141	57	1,091
C07	846,566	32	974
J10	792,980	82	952
L09	774,923	76	921
P20	720,523	65	838
L15	719,587	56	840
B11	674,250	47	815
M09	627,365	97	800
N17	328,144	45	397
C06	775,384	90	936

### The LD12 phylogeny reveals distinct microclusters

**Figure 1:**
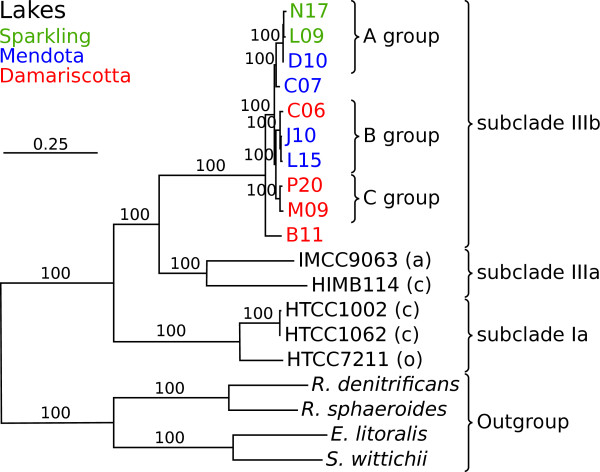
**Phylogenetic relationships of the LD12 SAGs.** The phylogenetic tree of the SAGs and the previously sequenced SAR11 genomes was inferred from an alignment of 57 concatenated pan-orthologous proteins. The abbreviations of SAG names from the Integrated Microbial Genomes (IMG) are as in Table S1 in Additional file 1. The subclades to which the SAR11 genomes belong have been defined previously [20] and are indicated with brackets to the right. The letter within parentheses indicates the water systems that the SAR11 cells were isolated from: (a) arctic waters, (c) coastal waters or (o) open ocean waters. All SAGs are from freshwater. Bootstrap support values less than 100% are not shown. The SAGs are colored according to the lake of origin (green = Sparkling; blue = Mendota; red = Damariscotta).

We first determined the phylogenetic placement of the 10 LD12 SAGs in relation to the genomes of the saltwater bacteria of the SAR11 clade. For this purpose, we selected 58 pan-orthologous proteins encoded by one copy per genome in 67 alphaproteobacterial genomes [9], of which 57 could be identified in the SAGs. The phylogeny, which was inferred with the maximum likelihood method, revealed three monophyletic clades (Figure 1). Subclade Ia and IIIa contained genomes from oceanic and coastal strains, while subclade IIIb contained the freshwater SAGs, consistent with previous phylogenies based on rRNA sequence data [7].

Within subclade IIIb, three distinct micro-clusters were indicated with strong bootstrap support values, here referred to as the A, B and C groups (Figure 1). The A group contains the N17, L09 and D10 SAGs obtained from lakes Sparkling and Mendota, the B group the C06, J10 and L15 SAGs from lakes Mendota and Damariscotta, and the C group the P20 and M09 SAGs from lake Damariscotta. The SAG designated C07 is a sister taxa to the A group SAGs with 100% bootstrap support, but was not classified as an A group strain since the node suggesting this placement was very short. Finally, the analyses placed the B11 SAG from lake Damariscotta as the earliest diverging SAG of those examined here.

### Hypervariable regions

**Figure 2:**
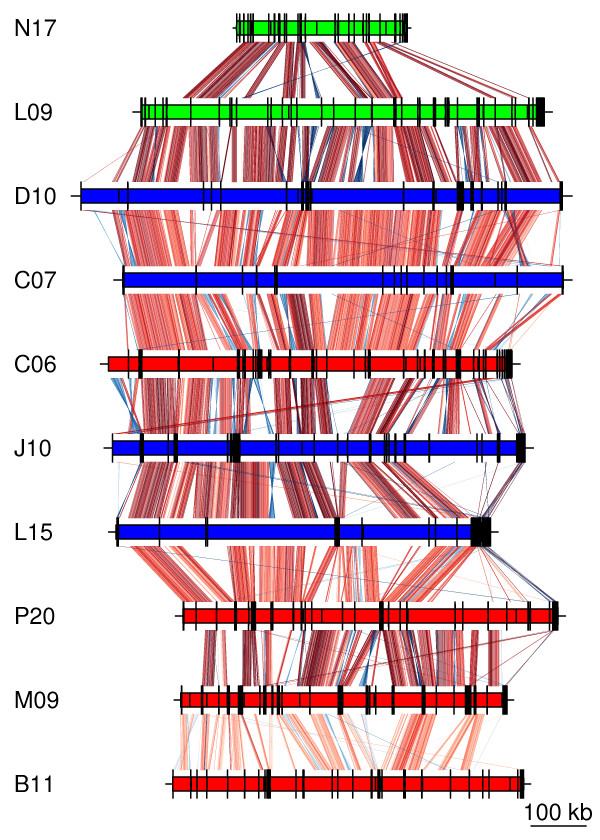
**Comparative genomics of the LD12 SAGs.** The SAGs are colored to indicate lake of origin, as in Figure 1. The scaffold blocks are separated by black lines. The colored lines between each of the SAGs and the patchwork genome indicate sequence similarities, with the intensity of the color reflecting the e-value of the blastn hit.

Pair-wise comparisons of the SAG assemblies revealed substantial overlaps between genomes, with long stretches of highly similar sequences (Figure 2). To search for recent changes in gene content related to the emergence of the LD12 clade, we performed a gene flux analysis in which we evaluated the gains and losses for the whole clade. To this end, we generated alphaproteobacterial protein clusters (α-COGs), including all LD12 SAGs, 5 marine SAR11 genomes and 61 other genomes that represent the main orders of the Alphaproteobacteria (Figure 1 in [9]). As the reference tree for the ancestral reconstruction, we used the tree topology presented in Figure 1 in [9], but replaced the single taxon *Ca.* Pelagibacter ubique with six taxa from the SAR11 clade. One of these taxa, here called ‘LD12’, was chosen to represent the total set of predicted proteins in all 10 SAGs, only excluding hypothetical genes solely present in a single SAG and not identified in any of the other genomes. Thus, the LD12 taxa in the reference tree contain most of the core genome of the LD12 clade and part of its pan-genome.

**Figure 3:**
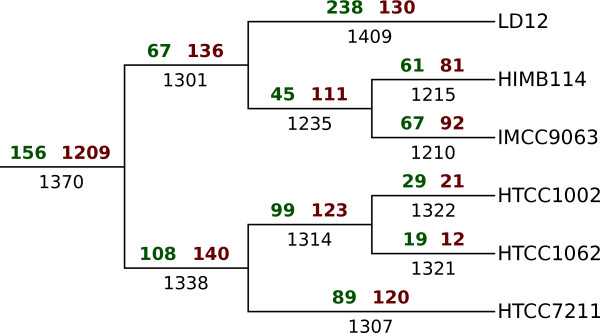
**Inference of deletions/duplications and gains in the evolution of the SAR11 clade.** The number on the top left of branches is gains (green), top right is losses (brown) and below the branch is presence. The losses and gains have been inferred with PAUP* using parsimony with cost 2 for gain and 1 for loss using accelerated transformation.

The occurrences of α-COGs were mapped onto the reference tree, with the gains and losses of clusters inferred according to the most parsimonious reconstruction of ancestral states, with a cost of 1 for loss and a cost of 2 for gain (Figure 3; Figure S1 in Additional file 1). Consistent with previous analysis, we inferred a dramatic loss of more than 1,200 protein clusters on the node separating the SAR11 clade from the rest of the Alphaproteobacteria, [9]. Genes for replication-repair processes such as the mismatch repair system present in all alphaproteobacterial genomes except *P. ubique*[9] could not be identified in the LD12 SAGs, and were therefore inferred to have been lost in the last common ancestor of the SAR11 clade.

We mapped 135 of the putatively gained and 113 of the putatively lost proteins to the COG families to infer their functions, of which 109 were not present in any of the other Alphaproteobacteria (Table 2). A total of 22 proteins were inferred as gained in the functional category for cell wall and cell membrane biosynthesis in the 10 LD12 SAGs, of which 9 mapped to COG families for glycosyltransferases that are involved in the transfer of sugar residues to other molecules, such as the outer variable O-antigen repeating unit of the lipopolysaccharide. A phylogeny of the glycosyltransferases indicated at least three distinct SAR11 clades that included both freshwater and marine isolates, indicative of vertical descent (Figure S2 in Additional file 1). In contrast, the glycosyltransferases classified as recently acquired were interspersed with sequences from other bacterial phyla, indicative of horizontal gene transfer.

**Table 2:** Functional categories of protein clusters inferred to have been gained and lost in the LD12 clade

**Information storage and processing**		**Gains**	**Losses**
A	RNA processing and modification	0	0
J	Translation, ribosomal structure and biogenesis	1	2
K	Transcription	5	0
B	Chromatin structure and dynamics	0	1
L	Replication, recombination and repair	7	2
	*Total*	*13*	*5*
**Metabolism**			
H	Coenzyme transport and metabolism	2	0
F	Nucleotide transport and metabolism	0	2
Q	Secondary metabolites biosynthesis, transport and catabolism	11	9
C	Energy production and conversion	8	8
P	Inorganic ion transport and metabolism	9	16
I	Lipid transport and metabolism	4	5
G	Carbohydrate transport and metabolism	12	12
E	Amino acid transport and metabolism	23	22
	*Total*	*69*	*74*
**Cellular processes and signaling**			
O	Posttranslational modification, protein turnover, chaperones	5	7
W	Extracellular structures	0	0
T	Signal transduction mechanisms	5	4
N	Cell motility	2	1
Y	Nuclear structure	0	0
V	Defense mechanisms	3	1
Z	Cytoskeleton	0	0
M	Cell wall/membrane/envelope biogenesis	22	6
D	Cell cycle control, cell division, chromosome partitioning	0	0
U	Intracellular trafficking, secretion, and vesicular transport	3	1
	*Total*	*40*	*20*
**Poorly characterized**			
S	Function unknown	15	14
R	General function prediction only	26	31
	*Total*	*41*	*45*
**Unmapped to the COG database**		103	17
**Mapped to the COG database**		135	113

**Figure 4:**
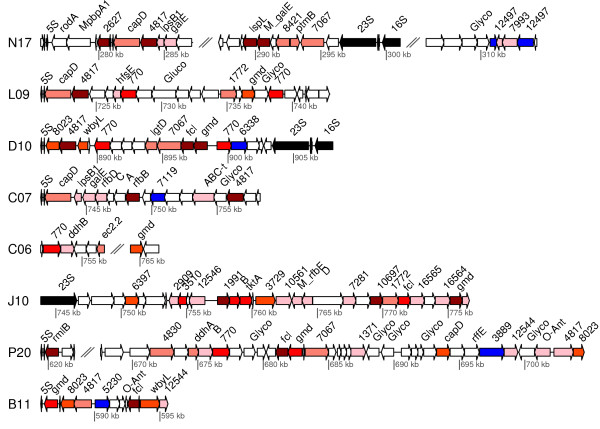
**Gene order structures of hypervariable segments.** SAG contigs with homologs to genes located in the hypervariable region HVR2 in the marine SAR11 genomes, as identified by BLAST search (E < e-10). The intensity of the red color indicates the number of SAR11 genomes for which a homolog was identified in HVR2 (pink = 1; dark red = 5). Blue color indicates glycosyltransferases identified as gained in LD12. Cluster annotations are shown, with cluster numbers used for clusters with no annotated gene name.

This is of particular interest since these and other genes involved in the biosynthesis of the lipopolysaccharide are located in HVRs in the marine SAR11 genomes [7]. One of these islands, referred to as HVR2, is about 50 kb in size and is flanked by the 5S rRNA gene on one side and the 16S and 23S rRNA genes on the other. Likewise, the acquired gene for glycosyltransferase in the D10 SAG is located in a region that also contains other lipopolysaccharide-associated genes and is flanked by the 5S rRNA and the 16S-23S rRNA genes (Figure 4). Four such regions in other SAGs contained either the 5S or the 23S rRNA genes and all contigs contained paralogs to genes located in the HVR2 region (E < e-10). Despite the similarity in broad functions there was little conservation in gene orders in these regions, contrasting with extensive synteny in most other chromosomal segments.

### Low recombination rates in freshwater bacteria of the SAR11 clade

**Figure 5:**
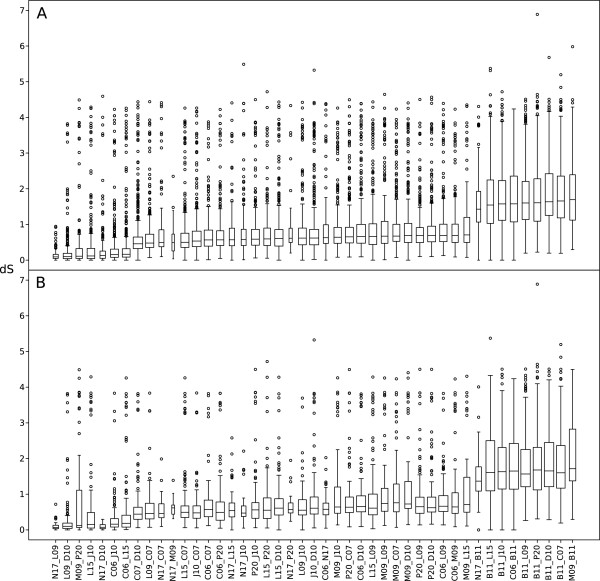
**Synonymous substitution frequencies.** Boxplot of dS values of **(A)** 775 and **(B)** 179 genes for all possible pairwise combinations of the 10 SAGs, ordered by the median values estimated for the dataset of 775 genes. The width of the boxes is normalized by the number of genes used for the calculations for each pair of SAGs.

To quantify the sequence divergence levels within and between the microclusters, we calculated synonymous substitution frequencies (dS) for all pairwise combinations of SAGs using a dataset of 775 genes that were present in at least 2 SAGs and 2 other SAR11 genomes (Figure 5). The median dS values for pairs of SAGs from within the same microcluster were <0.05 substitutions per site, whereas comparisons of SAGs from different microclusters yielded dS values of about 0.5 substitutions per site while all comparisons with B11 yielded dS values close to saturation. The dS values were relatively homogeneous among genes for any particular pair of SAGs, indicating that the observed divergences were mostly caused by single nucleotide substitutions.

**Figure 6:**
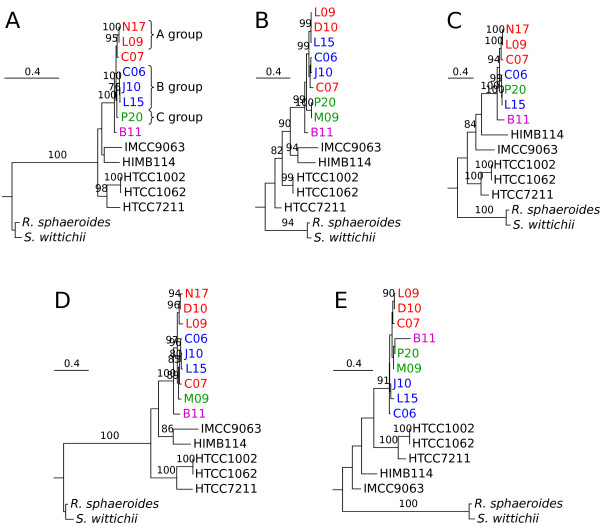
**Phylogenetic relationships of the LD12 SAGs inferred from single genes.** Five representative trees inferred from **(A)***groEL*, **(B)***dapE*, **(C)***priA*, **(D)***purH* and **(E)***nusA* that illustrate different clustering patterns of the SAGs. Abbreviations of the IMG names of the SAGs are as in Table S1 in Additional file 1. Twelve strains from Rhodobacterales and Sphingomonadales were used as outgroups, following Viklund *et al*. [9], but excluding *Hyphomonas neptunium* and *Maricaulis maris*. One representative outgroup species for each phylum is shown. Trees were inferred with the maximum likelihood approach. Only bootstrap support values higher than 75% are shown.

We also examined the congruence of single gene tree topologies. For this analysis, we selected a dataset of 179 gene clusters for which orthologs were present in at least i) two of the three SAGs in each of the A and B groups, ii) one of the two SAGs in the C group, iii) two isolates from each of the two marine SAR11 subclades, and iv) B11. We manually inspected 42 of the trees inferred from alignments of single genes >1 kb in size and for which orthologs were present in at least 11 of the 12 outgroups (Figure 6; Additional file 2). Most of these were fully congruent in topology with the concatenated tree, while the rest deviated in the placement of one or a few SAGs, possibly indicative of recombination events.

To quantify the influence of recombination to the divergences, we estimated the ratio at which recombination to mutations (*r/m*) contributes to the substitutions in the SAGs using ClonalFrame. For this analysis, we extracted sequences present in all SAGs except N17. After removal of segments with gaps using Gblocks, the alignment contained 31 genes in 25 kb of well aligned sequences (Table S2 in Additional file 1). Using this alignment, the *r/m* ratio was estimated to 0.14, which is at the lower end of *r/m* ratio estimates for bacteria. A re-analysis of 9 marine genomes using the same settings yielded a ratio of 61, consistent with the previously published estimate of 63 [1,5]. The higher recombination rate estimate for the marine strains was not simply an effect of a higher divergence in the sequences used for the tests since the mean dS value of the freshwater dataset was several-fold higher than for the marine dataset.

### Recruitment of lake metagenome sequences reveals the abundance profiles of the microclusters

To examine the relative abundance of LD12 microclusters in the environment, we analyzed metagenome sequence data from the three American lakes from which the single cells were sampled (Table 3). We also included metagenome sequence data from three Swedish lakes in the analysis. As observed previously, Actinobacteria and Betaproteobacteria dominated these metagenomes. The overall abundance of LD12 in Lake Erken was estimated to 16% based on an analysis of the rRNA sequences in the data set, while the other metagenomes contained less than 5% of such sequences. The coverage was two- to four-fold for Lakes Ekoln, Damariscotta (summer) and Mendota (spring), and more than ten-fold for reads recruited from the metagenome dataset of Lake Erken. Metagenome sequence reads associated with LD12 were recruited to a patchwork LD12 genome of 1.05 Mb, pieced together from the largest scaffolds in the genome assemblies (Figure S3 in Additional file 1).

**Table 3:** List of metagenome data sets, sizes and fractions of LD12

	**Preprocessed**		**Nucmer**^**a**^		
Lake	**Reads**	**Mb**	**Number**^**a**^	**Percentage reads**	**Cov**^**b**^
Damariscotta_spring	281,625	121	378	0.1%	0.2
Damariscotta_summer	323,939	140	5,268	1.6%	2.2
Ekoln	284,609	115	5,661	2.0%	2.2
Erken	554,862	233	29,262	5.3%	11.7
Mendota_spring	319,321	133	6,886	2.2%	2.7
Mendota_summer	447,054	192	2,063	0.5%	0.8
Vattern	285,637	117	3,031	1.1%	1.2
Sparkling_spring	66,160	26	43	0.1%	0.0
Sparkling_summer	38,977	15	815	2.1%	0.3

^a^Metagenomic reads recruited with the program Nucmer and filtered for >75% identity and >100 bp alignment.

^b^Cov = coverage, calculated using 1.054 Mb patchwork genome size.

**Figure 7:**
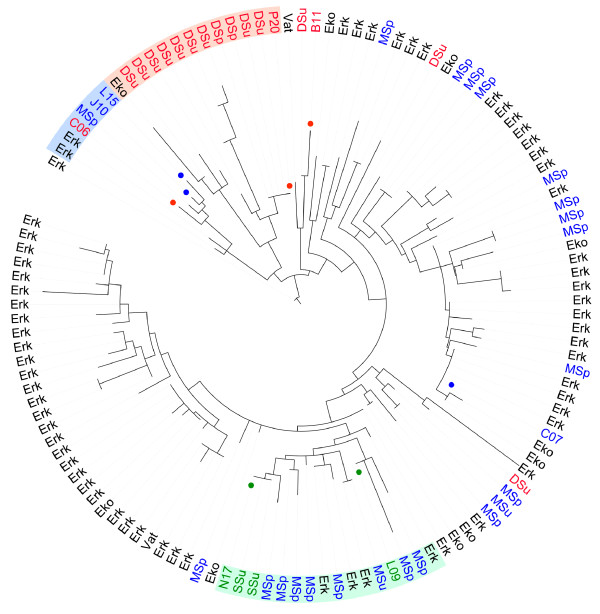
**Phylogenetic analysis of the *groEL *gene, including the LD12 SAGs and recruited metagenome sequences.** A phylogenetic inference of metagenomic reads with the reference nucleotide sequences from the LD12 SAGs indicated with circles (green = Sparkling; blue = Mendota; red = Damariscotta). The placements of metagenome reads affiliated with microclusters A, B and C, respectively, are highlighted. Abbreviations indicate the sampling season and the lake. SSu, Sparkling summer (green); DSu and DSp, Damariscotta summer and spring (red); MSu and MSp, Mendota summer and spring (blue). Erk, Erken; Eko, Ekoln; Vat, Vättern.

Phylogenetic trees were inferred from each of the 42 genes >1 kb, including both SAG and metagenome sequences (Additional file 3). The previously identified microclusters were clearly recognizable, as here exemplified with the *groEL* gene tree (Figure 7). In most trees, only a few reads were placed earlier than the divergence of the SAGs and even these were affiliated with LD12 as inferred from their short branch lengths, which confirms that we have sampled the diversity of LD12 without recruiting reads outside the SAR11 clade. Strikingly, the metagenome reads from the mesotrophic lake Damariscotta (summer) clustered almost exclusively with the C group, which consists of the Damariscotta-derived SAGs P20 and M09. In contrast, metagenome reads from the mesotrophic lake Erken were affiliated with both A and B groups, as well as with microclusters not represented by the SAGs analyzed here. Likewise, metagenome reads from lake Mendota (spring) and Ekoln were affiliated with several microclusters in the trees.

Finally, we inferred the population recombination (rho) and mutation (theta) rates separately for the total set of reads recruited from each lake to the patchwork genome using a composite likelihood method specifically developed for assembled metagenome sequences [21]. The analyses indicated a low, albeit measurable ratio of about 0.1 for the frequency of recombination relative to mutation events for each of the lakes. Such low genome-wide estimates for the LD12 lineage overall based on the metagenome data are consistent with the low *r/m* ratio estimated from the LD12 genome data.

### Possible artifacts and biases when using SAGs

SAGs are useful to identify novel genes and lineages, but due to their partial natural and uneven coverage, we were worried that artifacts could be introduced that would make them unsuitable for population genomic analyses.

One concern was that the frequency of erroneously called base pairs in SAGs is much higher than in conventionally sequenced genomes, which could compromise mutation and recombination rate estimates from closely related pairs of SAGs. Unfortunately, since the LD12 cells have not been cultivated, no reference genomes are available that could be used to estimate the error rate. Instead, we turned to a benchmarking data set consisting of seven SAGs from *Escherichia coli* and eight SAGs from each of *Meiothermus* and *Pedobacter*, for which reference genomes sequenced by conventional whole-genome sequencing techniques were available. All SAGs were sequenced with the Illumina sequencing technology, and the LD12 SAGs were sequenced to a greater depth than the benchmark SAGs, which suggest that the datasets are comparable.

We aligned the assembled contigs of the benchmark SAGs to their corresponding reference genomes and estimated the error frequency to 5 × 10^-6^ SNPs per base pair on average (Figure S4 in Additional file 1). For comparison, the median frequency of polymorphisms for SAGs of the same microcluster types were in the range of 13 and 16 SNPs per kb, as inferred by aligning each SAG sequence to the corresponding consensus microcluster sequence for each of the 42 genes used to estimate the synonymous substitution frequencies (Figure S5 in Additional file 1) as well as for the 25 kb fragments used to estimate the *r/m* ratio (Figure S5 in Additional file 1). Thus, the overall sequence divergence for the most closely related pairs of SAGs was estimated to 1.5 × 10^-2^ SNPs per base pair on average, which is more than 10,000-fold higher than the error rate. The extremely low error rate of the benchmark data suggests that the mutation and recombination rate estimates of the SAR11 SAGs have not been inflated by sequencing errors.

Another concern was that missing data due to the uneven coverage might cause problems in the phylogenies. For example, 57 of the 58 proteins used to infer the phylogenetic relationships of the SAGs were identified in 6 SAGs on average, and in 9 SAGs at most (Table S3 in Additional file 1). This means that each protein alignment will have data missing from one or more SAGs. The missing sequences were managed by introducing gaps in the concatenated protein sequence alignment. For the single protein phylogenies we specifically selected genes for which sequence data were available for two or more SAGs from the A and B groups and from one or more SAGs of the C group. This procedure made it possible to compare the single protein topologies with the concatenated protein topology. The overall congruence in topologies indicated that missing data for one or more SAGs have not affected the diversification patterns. Moreover, our estimates of the relative frequency of recombination to mutation were based on sequence data present in all 10 SAGs, with no missing data. Thus, neither the tree topologies nor the mutation and recombination rate estimates have been affected by the uneven sequence coverage.

More seriously, missing data could compromise the gene flux analysis since missing sequences could incorrectly be interpreted as absent genes. To minimize this problem we used the total set of genes identified in all 10 SAGs to represent the LD12 core genome, excluding open reading frames present in single SAGs with no hits to any other genome. If we assume that the amplification bias is random, we expect most of the core genes to have been sampled at least once in these SAGs. But even so, we cannot exclude that a few core genes were not sampled due to biased amplification.

Thus, in the gene flux analysis, the terminal taxa called LD12 contains proteins identified in all 10 SAGs. This is different from the gene content of the other terminal taxa, in which the gene count represents all genes in one complete genome. Hence, the actual number of gains and losses on the branch to LD12 in the gene flux analyses are not directly comparable to the numbers on the other branches, nor are they proportional to the branch length to the LD12 clade. Moreover, the relative fraction of gained and lost genes depends on the penalties for gains and losses. To test how the penalties affected the gene flux analysis, we increased the penalty for gain in a stepwise manner from 2 to 5, upon which the number of gained genes gradually decreased from 238 to 134 clusters (Table S4 in Additional file 1). This suggests that 134 protein clusters have almost certainly been gained, while another 104 clusters have tentatively been gained once in the freshwater group or lost twice or more in the marine clades. Irrespective of this, it can be concluded that these clusters are variably present in the bacteria of the SAR11 clade, as also indicated by the observation that several of these genes were located in regions that were homologous to a hypervariable region in the marine SAR11 genomes.

### Comparison of LD12 with human pathogen specialists

There is an interesting parallel between LD12 and human pathogen specialists in that both have low recombination frequencies and are nested within a group of much larger diversity. For example, *Salmonella enterica* has a broad host range of animals and a very high *r/m* ratio of 30.2 [1], that is, close to the *r/m* estimate for the marine SAR11 bacteria. In contrast, the human-restricted pathogen *S. enterica* serovar Typhi shows no evidence of recombination between isolates of Typhi [22], resembling the clonal population structure of the freshwater LD12 clade. Likewise, human pathogens such as *Yersinia pestis*[23], *Bacillus anthracis*[24], *Burkholderia mallei*[25], *Mycobacterium tuberculosis*[26] and *Bartonella quintana*[27] show extremely low levels of sequence diversity and are nested within genetically more diverse groups of bacteria with broader host ranges.

Most of the previously studied monomorphic lineages are pathogens, indicating a link between disease and transmission [28]. As pointed out, however, the association between low genetic diversity and disease could reflect a discovery bias [28]. Indeed, the identification of similar population dynamics in the SAR11 clade suggests that it is the bottleneck induced by the shift to a restricted growth niche, rather than the association with disease that purges the diversity in these populations irrespective of whether the adaptation is to a novel host or a new environment.

As these lineages adapt to their new niche, recombination with divergent members in the former environment is no longer possible. Recombination would then only happen within each adapting lineage, which would have little effect on genetic diversification, resulting in low *r/m* ratios. Moreover, each transmission event between host individuals may introduce new bottlenecks in case of the human pathogen specialists, depending on the mode of transmission. Likewise, geographic barriers and strong seasonal variability could potentially introduce additional bottlenecks after which recombination events will only occur between nearly identical genomes and may go undetected. This could lead to a situation in which each lake contains its own unique genotypes, with little mixing with strains from other lakes. The identification of a few highly abundant genotypes of microcluster type C in lake Damariscotta, not detected elsewhere, is consistent with this scenario. However, sequences affiliated with microclusters A and B could be identified in several geographically distant lakes, suggesting that dispersal occurs globally but that not all genotypes are present everywhere.

The overall sequence diversity of the classical monomorphic pathogens is in the range of only 0.1 to 2 SNPs per kilobase [28]. In comparison, we identified about 15 SNPs per kilobase in core genomes for the most closely related LD12 strains. One explanation for this difference could be that the transition from salt to freshwater occurred earlier than the specialization to a single host. Indeed, the dN/dS ratios are much below one for genes in the LD12 core genome, suggesting that there has been enough time for selection to act on the accumulation of mutations after the shift.

Other characteristic features of strict host-specialization are reductions of genome size and low rates of horizontal gene transfer. Unfortunately, due to biased amplification and partial data for the individual SAGs, the frequency of deletions cannot be determined with any precision until cultivated strains of LD12 with sequenced genomes are available. However, the identification of a highly variable region in the SAGs that is homologous to the hypervariable regions of the marine SAR11 strains indicate that horizontal transfers have influenced the LD12 genomes. This may not be surprising since lakes contain many other groups of bacteria that provide a rich source of novel genes. As suggested for the marine SAR11 genomes, it is possible that the hypervariable regions in the freshwater SAR11 genomes evolve under selection for diversity of surface structures to evade phages and predators [6,29]. If so, this suggests that selection for variability in surface structures need not necessarily be associated with high genome-wide recombination rates as in the marine SAR11 strains.

## Conclusion

We have shown in this study that the relative contribution of recombination to the observed substitutions in fresh and saltwater bacteria of the SAR11 clade differs by more than two orders of magnitude. These results are remarkable in that the *r/m* ratio for the freshwater genomes is in the lower range of such estimates for bacteria, whereas the saltwater genomes represent the upper end of the spectrum [1]. This implies that specialization to freshwater ecosystems has had a dramatic effect on the population dynamics of the SAR11 group of bacteria, much like the reduction in genetic diversity observed for human pathogen specialists.

## Materials and methods

### Sequencing and assembly

Single cells were sorted from freshwater samples and their DNA was amplified as described in Martinez-Garcia *et al*. [19]. The LD12 single-cell genomes were identified through sequencing of the 16S rRNA genes using universal primers.

Both the LD12 and the benchmark single-cell genomes were generated at the US Department of Energy Joint Genome Institute (JGI) using Illumina technology. An Illumina standard shotgun library was constructed and sequenced using the Illumina v3 HiSeq 2000 platform, run mode 2 × 150 bp. All general aspects of library construction and sequencing performed at the JGI can be found at [30]. Raw Illumina sequence data were filtered for known Illumina sequencing and library preparation artifacts and then screened and trimmed according to the k-mers present in the dataset. High-depth k-mers, presumably derived from the bias introduced by the multiple displacement amplification (MDA) reaction, cause problems in the assembly, especially if the k-mer depth varies by orders of magnitude for different regions of the genome. Reads representing highly abundant k-mers were removed such that no k-mers with a coverage of more than 30× were present after filtering. Reads with an average k-mer depth of less than 2× were removed.

The following steps were then performed for assembly. First, filtered Illumina reads were assembled using Velvet version 1.1.04 [31]. The VelvetOptimiser script (version 2.1.7) was used with default optimization functions (n50 for k-mer choice, total number of base pairs in large contigs for cov_cutoff optimization). Second, 1 to 3 kbp simulated paired-end reads were created from Velvet contigs using the wgsim software. Third, the normalized Illumina reads were assembled together with simulated read pairs using Allpaths-LG (version 41043) [32]. Parameters for assembly steps were: (i) VelvetOptimiser (--v --s 51 --e 71 --i 4 --t 1 --o ‘-ins_length 250 -min_contig_lgth 500’); (ii) wgsim (-e 0-1 100-2 100 -r 0 -R 0 -X 0); (iii) Allpaths-LG (prepareAllpathsParams: PHRED_64 = 1 PLOIDY = 1 FRAG_COVERAGE = 125 JUMP_COVERAGE = 25 LONG_JUMP_COV = 50, runAllpathsParams: THREADS = 8 RUN = std_pairs TARGETS = standard VAPI_WARN_ONLY = True OVERWRITE = True). The minimum coverage for a base to be called was 10 × .

### Bioinformatic analysis of SAG sequences

The gene predictions from the JGI annotation (accession numbers in Table S1 in Additional file 1) were the basis of the analysis. The name and function annotations were verified for consistency within larger protein clusters, and can vary from the JGI annotation for individual SAGs. The SAG proteomes were clustered using orthoMCL [33] (following the default user guidelines) together with five SAR11 genomes (*Ca.* Pelagibacter ubique HTCC1062, HTCC1002, HTCC7211, IMCC9063 and HIMB114) and the alphaproteobacterial genomes used in Viklund *et al*. [9]. Cluster annotations were based on ‘gene’ and ‘product’ NCBI annotations for all genes in the same cluster, and were used consistently for all cluster members.

#### Phylogeny

Protein sequences of 57 alphaproteobacterial pan-orthologs from the SAGs and the SAR11 genomes were aligned with the linsi option of MAFFT v6.864b [34] and masked with Gblocks [35] (default settings except allowing for gaps in at most half of the taxa). For each protein the best model was chosen with ProtTest [36]. Phylogenetic analysis of the concatenated protein alignment was performed with RAxML version 7.3.5 [37], using protein-specific protein models and the PROTCAT model with 100 quick bootstraps. Single gene trees were built with RAxML version 7.2.8 [37] using a GTRGAMMA model, with 100 bootstraps.

#### Substitution frequencies

Orthologous genes from orthoMCL present in at least two SAGs and two other SAR11 genomes were used for estimation of nucleotide sequence divergence levels in the LD12 population. The nucleotide sequences were aligned with the guidance of amino acid translations provided by TranslatorX [38] (−p F MAFFT v6.923 [34] linsi option). The pairwise synonymous substitution frequencies (dS) were calculated by the yn00 program from PAML v4.5 [39,40]. Sequences that covered less than 70% of the gene were removed, and also genes that had less than 70% of the alignment length used for the calculation were excluded, leaving a dataset of 775 genes.

#### Recombination

Whole-genome alignments of nine LD12 SAGs, excluding N17, were performed with progressiveMauve (Mauve v2.3.1) with default settings [41] and well-aligned blocks were further extracted by Gblocks v. 0.91 [35] with default stringent settings. The relative contribution of recombination and mutation to nucleotide substitutions (*r/m*) was estimated with ClonalFrame 1.2 [42] (three chains with the following number of generations, burn in and sampling frequency, respectively: -x 1000000 -y 1000000 -z 100). Convergence was assessed for all parameters and the *r/m* ratio was the same for each chain. SAR11 results were obtained from the multilocus sequence typing data published [5], using ClonalFrame with the same settings. dS values were calculated for the genes in the ClonalFrame input datasets (20 freshwater, 9 marine) and mean values per gene were tested with Mann–Whitney U test, to verify that higher recombination in marine dataset is not due to higher sequence diversity.

#### Gene flux

The occurrence matrix (that is, presence/absence disregarding number of copies) of orthologous groups was made for each genome based on the clustering described above. The most parsimonious ancestral presence/absence patterns were inferred using PAUP* (acctran option, gain cost 2, loss cost 1). The patterns were plotted on the tree using Treegraph 2 [43]. Gains and losses inferred on the LD12 branch were annotated with hmmer [44] (NCBI COG database as queries against the clusters database, e-value cutoff 10^-10^) choosing the best match for each cluster as the representative COG.

### Bioinformatic analysis of the metagenomes

The largest scaffolds from different SAGs were checked for substantial overlaps to obtain the relative ordering. The starting point was the largest 250 kb scaffold from cell L15. Additional scaffolds were added in phylogenetic order, if there was a choice, resulting in the eight largest L15 scaffolds constituting the patchwork and complemented with sequence from the other SAGs. The process of joining overlapping scaffolds continued until all information from substantial overlaps (>10 kb) was utilized (Table S1 in Additional file 1). Non-overlapping or small scaffolds were placed at the end of each of the individual SAGs.

#### Recruitment

Metagenomic reads were recruited to the patchwork with nucmer [45] and further filtered (>75% identity and >150 bp alignment and >50% read length aligned). Cluster-based annotation of patchwork genes was used to obtain the corresponding SAG and other SAR11 sequences. Before phylogenetic analysis, metagenomic reads were cut to the gene boundary, removing reads with <150 bp overlap. The use of different SAGs as the reference for different regions was verified to have little effect on the recruitment results.

#### Phylogenies

Nucmer alignments of the metagenomic reads were used for phylogenies. Reference SAG and other SAR11 nucleotide sequences were aligned with the guidance of amino acid translations by TranslatorX [38] (−p F MAFFT v6.92 [34] linsi option). The metagenomic and the genomic alignment blocks were merged with mafft-profile [46]. Maximum likelihood phylogenies with 100 bootstrap replicates were obtained using RAxML version 7.2.8 with the GTRGAMMA model [37].

#### Recombination

Population recombination (rho) and mutation (theta) rates for the recruited reads were inferred by PIIM [21] (sequential mode, theta starting points 0.01 and 0.1). Xml input files for PIIM were generated from nucmer alignments and the corresponding read quality files.

### Accession numbers

The SAR11 SAGs listed in Table 1 have been deposited in GenBank under accession IDs: ATTB00000000, ATTD00000000, ATTC00000000, AQPD00000000, AQUE00000000, AQUF00000000, AQUG00000000, AQUH00000000, AQZA00000000, AZOF00000000. The benchmark data set is available on the JGI portal [30] under proposal ID 300869.

## Abbreviations

α-COG: Alphaproteobacterial cluster of orthologous genes; bp: base pair; HVR: hypervariable region; JGI: Joint Genome Institute; PP: Posterior probability; SAG: Single amplified genome; SNP: single nucleotide polymorphism.

## Competing interests

The authors declare that they have no competing interests.

## Authors’ contributions

KZN analyzed the SAGs, compared the hypervariable regions, pieced together the patchwork genome and performed the metagenomic analyses. JV clustered the proteins, inferred the concatenated and glycosyltransferase phylogenies, and performed the gene flux analysis. WZ calculated synonymous substitution frequencies, inferred single gene phylogenies and estimated the ratio of recombination to mutation. JA and JV performed pilot experiments. AS and TW sequenced, assembled and annotated the SAGs. KM and SB contributed samples and metagenome sequence data. RS performed the single cell sorting and the identification, extraction and amplification of genomic DNA from the LD12 cells. SB, RS, KM and SGEA planned the sequencing project of the LD12 SAGs. SGEA designed the study, helped interpret the data and wrote the paper, with contributions from KZN, JV, WZ, TW, SB and RS. All authors read and approved the final manuscript.

## Supplementary Material

Additional file 1: Table S1 SAG assembly and annotation data from Integrated Microbial Genomes pipeline [47]. **Table S2.** Sequences included in the mutation to recombination analysis with ClonalFrame. **Table S3.** Number of SAGs for each of the 57 proteins used in the concatenated protein alignment. **Table S4.** Protein clusters putatively gained in gene flux analyses with penalties for gain ranging from 2 to 5. **Figure S1.** Gene flux analysis of the number of clusters gained (top left), lost (top right) and the total number of clusters at the node (below the branch). **Figure S2.** Phylogenetic inference of glycosyltransferases with the maximum likelihood method. Highly supported SAR11 clades are highlighted in dark yellow and recently acquired glycosyltransferases are highlighted in blue. **Figure S3.** Visualization of the similarity between the patchwork genome and each of the individual SAGs with genoPlotR [48]. The colored lines between each of the SAGs and the patchwork genome indicate sequence similarities, with the intensity of the color reflecting the e-value of the blastn hit. Blue color show inverted segments. **Figure S4.** Boxplot of error frequencies, estimated as the number of **(A)** global SNPs and **(B)** total SNPs per assembled Mb in seven to eight independently sequenced SAGs compared to the corresponding reference genomes from *Escherichia coli* (Eco), *Meiothermus* (Mru) and *Pedobacter* (Phe). Global SNPs were counted as the number of SNPs flanked by a 20 bp stretch of 100% identical sequences. **Figure S5.** Boxplot of substitution frequencies, estimated as the number of SNPs per kilobase in each SAG compared to the corresponding microcluster consensus sequence for two datasets; the 42 genes >1 kb in length used for the single gene trees (median 13.4 and mean 15.7 SNPs/kb) and the 25 kb fragment used for the recombination to mutation analysis with ClonalFrame (median 15.9 and mean 36.3 SNPs/kb). Click here for file

Additional file 2 **Phylogenetic analysis of 42 genes from the LD12 SAGs.** A phylogenetic inference of genes from the LD12 SAGs. Abbreviations of SAGs show microcluster and the name and cluster number from Integrated Microbial Genomes (IMG). Click here for file

Additional file 3 **Phylogenetic analysis of 42 genes from the LD12 SAGs and recruited metagenome sequences from six lakes.** A phylogenetic inference of genes from the LD12 SAGs and affiliated genes obtained from the metagenomic dataset. Abbreviations of SAGs show microcluster, and the name and cluster number from Integrated Microbial Genomes (IMG). Abbreviations of metagenomic sequences indicate the lake and the sampling season. SSu = Sparkling summer (green); DSu and DSp = Damariscotta summer and spring (red); MSu and MSp = Mendota summer and spring (blue); Erk = Erken; Eko = Ekoln; Vat = Vättern. The marine SAR11 strains were used as outgroups (not shown). Click here for file
