# Supplementary material for: Single-cell genomics reveal low recombination frequencies in freshwater bacteria of the SAR11 clade
Source: Genome Biol. 2013 Nov 28;14(11):R130. doi: 10.1186/gb-2013-14-11-r130 (PMC4053759; doi:10.1186/gb-2013-14-11-r130)
Supplement: Additional file 1: Table S1 — SAG assembly and annotation data from Integrated Microbial Genomes pipeline [47]. Table S2. Sequences included in the mutation to recombination analysis with ClonalFrame. Table S3. Number of SAGs for each of the 57 proteins used in the concatenated protein alignment. Table S4. Protein clusters putatively gained in gene flux analyses with penalties for gain ranging from 2 to 5. Figure S1. Gene flux analysis of the number of clusters gained (top left), lost (top right) and the total number of clusters at the node (below the branch). Figure S2. Phylogenetic inference of glycosyltransferases with the maximum likelihood method. Highly supported SAR11 clades are highlighted in dark yellow and recently acquired glycosyltransferases are highlighted in blue. Figure S3. Visualization of the similarity between the patchwork genome and each of the individual SAGs with genoPlotR [48]. The colored lines between each of the SAGs and the patchwork genome indicate sequence similarities, with the intensity of the color reflecting the e-value of the blastn hit. Blue color show inverted segments. Figure S4. Boxplot of error frequencies, estimated as the number of (A) global SNPs and (B) total SNPs per assembled Mb in seven to eight independently sequenced SAGs compared to the corresponding reference genomes from Escherichia coli (Eco), Meiothermus (Mru) and Pedobacter (Phe). Global SNPs were counted as the number of SNPs flanked by a 20 bp stretch of 100% identical sequences. Figure S5. Boxplot of substitution frequencies, estimated as the number of SNPs per kilobase in each SAG compared to the corresponding microcluster consensus sequence for two datasets; the 42 genes >1 kb in length used for the single gene trees (median 13.4 and mean 15.7 SNPs/kb) and the 25 kb fragment used for the recombination to mutation analysis with ClonalFrame (median 15.9 and mean 36.3 SNPs/kb). [file gb-2013-14-11-r130-S1.pdf]

Supplementary Table S1

SAG assembly and annotation data

cell	D10	C07	J10	L09	P20	L15	B11	M09	N17	C06
Lake	Mendota	Mendota	Mendota	Sparkling	Damariscotta	Mendota	Damariscotta	Damariscotta	Sparkling	Damariscotta
Collected (2009)	May 19	May 19	May 19	May 28	Aug 19	May 19	Aug 19	Aug 19	May 28	Aug 19
IMG name	alpha proteobacterium SCGC AAA028-D10	alpha proteobacterium SCGC AAA028-C07	alpha proteobacterium SCGC AAA027-J10	alpha proteobacterium SCGC AAA023-L09	alpha proteobacterium SCGC AAA280-P20	alpha proteobacterium SCGC AAA027-L15	alpha proteobacterium SCGC AAA280-B11	alpha proteobacterium SCGC AAA487-M09	alpha proteobacterium SCGC AAA024-N17	alpha proteobacterium SCGC AAA027-C06
Assembly size bp	925,141	846,566	792,980	774,923	720,523	719,587	674,250	627,365	328,144	775,384
Scaffolds	57	32	82	76	65	56	47	97	45	90
Genes	1091	974	952	921	838	840	815	800	397	936
Proteins	1057	955	921	892	814	821	797	776	383	916
rRNA genes[a]	5,16,23S	5S	5,16,23S	5S, 23S, 23S, 23S	5,16,23S	-	5,16,23S	-	5S,5S, 16S,23S	16S, 23S,23S
Largest scaffolds bp (GC)	176,148 (0.29) 145,420 (0.29) 126,387 (0.29) 103,388 (0.31)	203,146 (0.29) 138,806 (0.30)	96,767 (0.30) 91,089 (0.31) 69,426 (0.29) 52,972 (0.31)	59,964 (0.29) 54,879 (0.32)	90,870 (0.30) 48,602 (0.31) 48,167 (0.28)	245,217 (0.30) 126,188(0.28) 88,810 (0.29) 79,072 (0.30)	54,526 (0.28) 53,946 (0.31)	54,427 (0.28) 54,274 (0.29)	35,260 (0.28) 29,946 (0.30)	70,157 (0.30) 65,619 (0.29) 64,320 (0.29) 52,346 (0.29)
microcluster	A		B	A	C	B		C	A	B
Patchwork contruction (patchwork start-end):scaffold:start-end:orientation	(1-42082)sc5:21559-63640:1 (693535-695310)sc6:17015-18790:-1 (944309-963781)sc4:72945-92417:-1 (967411-1035006)sc4:1309-68904:-1	(120287-138346)sc2:68210-86269:1 (470765-525902)sc1:45082-100219:1 (555014-569229)sc1:134439-148654:1	(223342-227654)sc1:62601-66913:1 (705898-723218)sc12:3330-20650:1 (827988-862604)sc6:5222-39838:1 (879880-944308)sc2:16613-81041:-1 (963782-967410)sc18:4512-8140:-1	(862605-879879)sc7:17273-34547:1	(785837-827987)sc1:15416-57566:1	(42083-120286)sc4:237-78440:1 (138347-223341)sc3:2869-87863:1 (227655-470764)sc1:85-243194:1 (525903-555013)sc5:10515-39625:-1 (569230-693534)sc2:1153-125457:1 (695311-	(1035007-1054073)sc5:16867-35933:-1	(753541-760945)sc1:14137-21541:-1		

						705897)sc8:671-11257:-1 (723219-753540)sc6:10247-40568:-1 (760946-785836)sc7:1714-26604:-1				

[a] Multiple annotations indicate partial genes located on different contigs

Supplementary Table S2

Sequences included in the analysis with ClonalFrame

Block	Position in patchwork genome[a]	Gene name
1	108973..110196	coaBC
2-1	110196..110636	dut
2-2	110643..111395	moeB
3	111392..111871	485[b]
4-1	111943..112449	fabA
4-2	112459..113673	fabB
5	113684..114463	fabI
6 & 7-1	c(114460..116535)	pnp
7-2	c(116542..116811)	rpsO
8	c(116815..117693)	truB
9-1	c(117686..118063)	rbfA
9-2	c(118070..120286)	infB
10	143515..144288	dapB
11	144318..144956	nth
12	c(145907..146539)	11939[b]
13 & 14	150239..152770	leuS
15	152760..153224	23820[b]
16-1	153230..154252	[b]
16-2	c(154211..155077)	parB
17-1	c(155078..155878)	parA
17-2	c(155887..156516)	gidB
18	c(156522..158390)	gidA
19	c(158438..159763)	trmE
20-1	c(309116..310042)	acuC
20-2	c(310039..311199)	dapE
21	347456..348757	11936[b]
22	348771..349685	OG11893[b]
23	c(350465..351775)	OG11892[b]
24-1	503872..504540	23783[b]
24-2 & 25	504544..505776	OG11235[b]
26	507641..509677	ligA

[a] Numbers listed give full-length gene position in the patchwork genome, from which gaps were removed by Gblocks as described in Methods. To obtain position in SAGs find these positions in patchwork construction from SAGs (Supplementary Table S5), for example coaBC matches scaffold 4 from cell L15.

[b] Genes without a gene name were not used for the sequence divergence test.

Supplementary Table S3

Number of SAGs for each of the 57 proteins used in the concatenated protein alignment.

aspS	6
ftsQ	0
gidA	9
glyS	7
hypA	6
hypB	8
hypC	6
hypD	6
hypE	9
hypF	6
hypG	5
hypH	6
ksgA	7
ligA	9
nusA	9
nusB	6
obgE	5
pcnB	9
pheT	6
pnp	9
pth	8
recR	6
rnc	4
rplA	4
rplI	6
rplJ	4
rplK	4
rplL	3
rplM	4
rplO	4
rplQ	7
rplR	4
rplS	7
rplU	5
rplY	8
rpmA	4
rpmE	6
rpmI	6

rpoA	7
rpoB	5
rpsB	8
rpsD	4
rpsE	4
rpsH	5
rpsI	4
rpsK	4
rpsN	5
rpsO	9
rpsP	7
rpsT	8
rrf	8
secY	6
serS	7
smpB	4
thdF	9
tig	5
trmD	7
tsf	8

Supplementary Table S4

Protein clusters putatively gained in gene flux analyses with penalties for gain ranging from 2 to 5.

Cluster	G 2	A 3	I 4	N 5	Gene	Product
LDA01479	1	0	0	0	NA	hypothetical:19, rubrerythrin:9, transmembrane:1
LDA01850	1	0	0	0	livG:3	ABC:16, branched-chain:5, ABC-type:2, amino:1, branched:1, high-affinity:1
LDA01890	1	0	0	0	livG:1	ABC:17, branched-chain:5, inner-membrane:1, branched:1, glutelin:1, high-affinity:1
LDA02108	1	0	0	0	NA	hypothetical:11, transporter,:2, inner:2, conserved:1, transmembrane:1
LDA02119	1	0	0	0	mntH:4	manganese:20, Mn2+/Fe2+:2, manganese/divalent:1
LDA02133	1	0	0	0	NA	ABC:8, nitrate/sulfonate/bicarbonate:4, NMT1/THI5-like:3, extracellular:2, sulfonate/nitrate:1, amino:1, putative:1, substrate-binding:1, taurine-binding:1
LDA02178	1	0	0	0	ansA:2	L-asparaginase:12, hypothetical:7
LDA02194	1	0	0	0	NA	UDP-glucose/GDP-mannose:12, UDP-glucose:2, udpglucosegdpmannose:1, putative:1, nucleotide:1, UDP-N-acetyl-D-mannosaminuronic:1, UDP-N-acetyl-D-mannosaminuronate:1, nucleotide-sugar:1, udpglucose:1
LDA02200	1	0	0	0	NA	ABC:18, amino:1, ABC-transport:1, taurine:1
LDA02201	1	0	0	0	NA	ABC:10, binding-protein-dependent:7, binding-protein:2, amino:1, taurine:1
LDA02298	1	0	0	0	dppA:3, dppAch1:1, dppA1:1	extracellular:7, periplasmic:4, dipeptide:3, peptide:3, 4-phytase:2, ABC:1, hypothetical:1, peptide/opine/nickel:1
LDA02381	1	0	0	0	gatA1:1, gatA:1	amidase:17, amidotransferase:1, glutamyl-tRNA(Gln):1
LDA02391	1	0	0	0	NA	hypothetical:10, membrane-associated:2, sulfatase:2, phosphatidylethanolamine:Kdo2-lipid:1
LDA02440	1	0	0	0	rffE:4	UDP-N-acetylglucosamine:18, lipopolysaccharide:1, putative:1, UDP-GlcNAc-2-epimerase.:1, udp-n-acetylglucosamine:1
LDA02514	1	0	0	0	dctQ:3	tripartite:9, TRAP:4, TRAP-type:1
LDA02517	1	0	0	0	NA	hypothetical:12, signal:1, MtN3:1
LDA02611	1	0	0	0	NA	branched-chain:4, extracellular:3, ABC:2, twin-arginine:2, ABC-type:2, amino:1, Leu/Ile/Val-binding:1, branched:1, leucine-specific:1
LDA02738	1	0	0	0	NA	hypothetical:12
LDA02740	1	0	0	0	hsdR:7	type:7, Type:6, HsdR:4, putative:2, DEAD/DEAH:1
LDA02798	1	0	0	0	NA	hypothetical:12, lipoprotein:2, signal:1
LDA02823	1	0	0	0	gltJ:4, gltJ1:1, gltJ2:1	amino:8, glutamate/aspartate:4, polar:3, ABC:2
LDA02925	1	0	0	0	NA	oxidoreductase:6, Gfo/Idh/MocA:4, oxidoreductase-like:3, NAD-binding:1, hypothetical:1, trans-1,2-dihydrobenzene-1,2-diol:1
LDA03005	1	0	0	0	NA	hypothetical:14, DMT:3
LDA03006	1	0	0	0	NA	hypothetical:5, branched-chain:3, branched:2, putative:1, leucine-,:1, Fis:1

LDA03008	1	0	0	0	NA	inner-membrane:12, branched:2, ABC:1, branched-chain:1, transmembrane:1
LDA03124	1	0	0	0	gltK:2, gltK2:1, gtpC:1, gltK1:1	polar:5, glutamate/aspartate:4, ABC:3, amino:3
LDA03154	1	0	0	0	NA	hypothetical:14, signal:1
LDA03268	1	0	0	0	NA	hypothetical:13, TRAP-T:1, transmembrane:1
LDA03313	1	0	0	0	NA	molybdopterin:6, sulfite:4, twin-arginine:2, oxidase:1, oxidoreductase,:1
LDA03330	1	0	0	0	NA	hypothetical:9, transmembrane:1
LDA03331	1	0	0	0	fer2:1	ferredoxin,:4, ferredoxin-like:2, (2Fe-2S):2, ferredoxin:2
LDA03501	1	0	0	0	NA	glycosyl:11, glycosyltransferase:3, dolichol-p-glucose:1
LDA03547	1	0	0	0	NA	hypothetical:12, integral:1
LDA03637	1	0	0	0	adh:1	alcohol:9, zinc-containing:1, zinc-type:1
LDA03718	1	1	0	0	pldA:1	phospholipase:5, hypothetical:4, cardiolipin:2, phosphatidylserine/phosphatidylglycerophosphate/:1, nuclease-related:1
LDA03823	1	0	0	0	rci:1	phage:5, integrase:2, DNA:2, Phage:1, integrase/recombinase:1, site-specific:1, shufflon-specific:1
LDA03889	1	0	0	0	NA	glycosyl:4, group:4, glycosyltransferase:2, putative:1
LDA03891	1	0	0	0	NA	spore:5, hypothetical:2, late-developmental:1, protein:1
LDA03906	1	1	0	0	bcpB:1	thiol-specific:2, BcpB:2, redoxin:1, thioredoxin:1
LDA04129	1	0	0	0	gltL:4, gltL1:1, gltL2:1	glutamate/aspartate:4, ABC:3, glutamate:2, amino:1
LDA04183	1	0	0	0	lpeA:1	methyltransferase:4, hypothetical:4, putative:2, lipolysaccharide:1, SAM-dependent:1
LDA04204	1	1	0	0	NA	hypothetical:11
LDA04259	1	0	0	0	NA	hypothetical:7, membrane:1
LDA04351	1	0	0	0	traF:4	hypothetical:6, F:2, conjugative:2, TraF-like:1
LDA04391	1	1	0	0	braC:1, braC1:1, livJ:1	extracellular:3, ABC:1, amino:1, solute-binding:1, putative:1, leucine-,:1, Leu/Ile/Val-binding:1, branched-chain:1
LDA04406	1	0	0	0	fimD:1	hypothetical:4, P:3, outer:1, fimbrial:1
LDA04536	1	0	0	0	chrR:1	hypothetical:3, transcription:2, anti-sigm:1, allophanate:1, anti-ECFsigma:1
LDA04774	1	0	0	0	NA	major:8
LDA04805	1	1	0	0	NA	hypothetical:3
LDA04976	1	1	0	0	NA	methyltransferase:4, hypothetical:3, putative:2
LDA05211	1	1	1	0	NA	hypothetical:8, acetamidase/formamidase:1
LDA05213	1	0	0	0	traH:2	pilus:3, F:2, hypothetical:2, TraH:2
LDA05222	1	0	0	0	NA	6-phosphofructokinase:5, pyrophosphate-dependent:1
LDA05230	1	0	0	0	NA	group:4, glycosyl:2, glycosyltransferase-like:1, hypothetical:1, glycosyltransferase:1
LDA05245	1	0	0	0	corAf:1	magnesium:2, metal:2, metal-transport:2, transmembrane:1, CorA-like:1, magnesium/cobalt:1
LDA05607	1	1	0	0	NA	hypothetical:2, putative:1, putataive:1, Pilus:1, chaperone:1
LDA05671	1	1	1	1	NA	NA
LDA05745	1	0	0	0	NA	hypothetical:6
LDA05815	1	0	0	0	lpsB1:1	sugar:3, putative:1, galactosyltransferase:1, hypothetical:1, glycosyltransferase:1

LDA06129	1	0	0	0	NA	hypothetical:4, TPR:2, serine/threonine:1
LDA06319	1	1	1	1	NA	NA
LDA06338	1	1	0	0	NA	glycosyltransferase:3, glycosyl:1, group:1, alpha-glycosyltransferase:1
LDA06377	1	1	1	1	NA	NA
LDA06381	1	1	1	1	NA	NA
LDA06386	1	1	1	1	NA	NA
LDA06387	1	1	1	0	NA	hypothetical:4
LDA06397	1	1	1	0	NA	epimerase:2, dTDP-glucose:1, NAD-dependent:1, hypothetical:1, epimerase/dehydratase:1, NDP-glucose:1
LDA06398	1	1	0	0	NA	hypothetical:1
LDA06704	1	0	0	0	NA	thioredoxin:4, thioredoxin-related:1, hypothetical:1, thioredoxin-like:1
LDA06830	1	0	0	0	NA	acetaldehyde:6
LDA06989	1	0	0	0	NA	cephalosporin:3, hypothetical:3
LDA07069	1	0	0	0	NA	hypothetical:6
LDA07073	1	0	0	0	NA	acid:3, nonspecific:1, Acid:1, PA-phosphatase-like:1
LDA07079	1	0	0	0	NA	hypothetical:5
LDA07083	1	1	1	1	NA	NA
LDA07085	1	1	0	0	NA	extracellular:3, ABC:2, twin-arginine:1
LDA07086	1	1	1	1	NA	NA
LDA07088	1	1	1	1	NA	NA
LDA07119	1	1	0	0	NA	group:4, hypothetical:1, glycosyltransferase:1
LDA07273	1	1	1	1	NA	NA
LDA07274	1	1	1	1	NA	NA
LDA07275	1	1	1	1	NA	NA
LDA07279	1	1	1	1	NA	NA
LDA07280	1	1	1	1	NA	NA
LDA07283	1	1	1	1	NA	NA
LDA07477	1	1	1	1	NA	NA
LDA07478	1	1	1	0	NA	hypothetical:6
LDA07527	1	1	0	0	NA	hypothetical:6
LDA07627	1	1	0	0	NA	hypothetical:3, dienelactone:1, dipeptidyl:1
LDA07689	1	0	0	0	NA	hypothetical:3, tetratricopeptide:2
LDA07691	1	0	0	0	NA	4-hydroxy-2-ketovalerate:4, pyruvate:1
LDA07958	1	0	0	0	NA	FkbM:3, methyltransferase:1
LDA08012	1	1	1	1	NA	NA
LDA08016	1	1	1	0	NA	hypothetical:2
LDA08020	1	1	1	1	NA	NA
LDA08107	1	1	0	0	NA	hypothetical:5
LDA08179	1	1	1	1	NA	NA
LDA08180	1	1	1	1	NA	NA
LDA08183	1	1	0	0	NA	S-adenosyl-L-methionine-dependent:1, hypothetical:1, 2-polyprenyl-3-methyl-5-hydroxy-6-metoxy-1,4-:1, Generic:1
LDA08184	1	1	1	0	NA	putative:2, signal:1
LDA08185	1	1	1	1	NA	NA
LDA08186	1	1	1	1	NA	NA
LDA08189	1	1	1	1	NA	NA
LDA08191	1	1	1	1	NA	NA
LDA08375	1	1	1	1	NA	hypothetical:5
LDA08421	1	0	0	0	NA	sialic:1, polysaccharide:1, N-acylneuraminate-9-phosphate:1
LDA08484	1	1	1	1	NA	NA
LDA08537	1	1	1	1	NA	hypothetical:5
LDA08588	1	1	0	0	NA	DNA:3, hypothetical:1, adenine-specific:1
LDA08738	1	1	1	1	NA	NA

LDA09008	1	1	1	0	NA	ABC:3, binding-protein-dependent:1
LDA09104	1	1	1	0	NA	TRAP:4
LDA09128	1	1	1	1	NA	NA
LDA09129	1	1	1	1	NA	NA
LDA09130	1	1	1	1	NA	NA
LDA09133	1	1	1	1	NA	NA
LDA09134	1	1	1	1	NA	NA
LDA09135	1	1	0	0	NA	YceI:3
LDA09270	1	1	1	1	NA	NA
LDA09273	1	1	1	1	NA	NA
LDA09274	1	1	1	1	NA	NA
LDA09275	1	1	1	1	NA	NA
LDA09276	1	1	1	1	NA	NA
LDA09277	1	1	1	1	NA	NA
LDA09278	1	1	1	1	NA	NA
LDA09280	1	1	1	1	NA	NA
LDA09318	1	0	0	0	NA	SAM-dependent:2, type:2
LDA09577	1	1	1	1	NA	NA
LDA09691	1	1	1	1	NA	NA
LDA09965	1	1	1	0	NA	hypothetical:3
LDA09996	1	0	0	0	NA	NAD-dependent:4
LDA10041	1	1	0	0	NA	hypothetical:2, O-methyltransferase:1
LDA10190	1	1	1	1	NA	hypothetical:2, capsule:1
LDA10552	1	1	1	1	NA	NA
LDA10557	1	1	1	1	NA	NA
LDA10562	1	1	1	1	NA	NA
LDA10583	1	1	1	1	NA	NA
LDA10610	1	0	0	0	NA	NAD-dependent:1
LDA10694	1	1	1	1	NA	NA
LDA10696	1	1	1	1	NA	NA
LDA10714	1	1	1	1	NA	hypothetical:2, FkbM:1
LDA11154	1	1	1	1	NA	NA
LDA11155	1	1	1	1	NA	NA
LDA11157	1	1	1	1	NA	hemolysin:1
LDA11159	1	1	1	1	NA	NA
LDA11483	1	1	1	0	NA	abortive:2
LDA11795	1	1	1	1	NA	NA
LDA12080	1	1	1	1	NA	NA
LDA12227	1	1	1	1	NA	hypothetical:1
LDA12497	1	1	0	0	NA	glycosyltransferase:1
LDA12538	1	1	1	1	NA	NA
LDA12541	1	1	1	1	NA	NA
LDA12542	1	1	1	0	NA	cytochrome:1
LDA12547	1	1	1	1	NA	NA
LDA12549	1	1	1	1	NA	MOSC:1, hypothetical:1
LDA12559	1	1	1	0	NA	formate:2
LDA12573	1	1	1	0	NA	lipopolysaccharide:1
LDA12594	1	1	1	1	NA	NA
LDA12695	1	1	1	1	NA	NA
LDA12696	1	1	1	1	NA	NA
LDA12697	1	1	1	1	NA	NA
LDA12698	1	1	1	1	NA	NA
LDA13213	1	1	0	0	NA	hypothetical:2

LDA13215	1	1	1	1	NA	NA
LDA13216	1	1	1	1	NA	NA
LDA13241	1	1	1	1	NA	regulatory:1, nitrogen:1
LDA13406	1	1	1	1	NA	short-chain:2
LDA13536	1	1	1	1	NA	NA
LDA13652	1	1	1	1	NA	NA
LDA13653	1	1	1	0	NA	TRAP:1
LDA13654	1	1	0	0	NA	hypothetical:1, glycosyl/methyltransferase:1
LDA13656	1	1	1	1	NA	NA
LDA13859	1	1	1	1	NA	hypothetical:1
LDA13860	1	1	1	1	NA	hypothetical:1
LDA13861	1	1	1	1	NA	hypothetical:1
LDA13862	1	1	1	1	NA	serine:1
LDA14385	1	1	1	1	NA	NA
LDA14386	1	1	1	1	NA	NA
LDA14387	1	1	1	1	NA	NA
LDA14388	1	1	1	1	NA	NA
LDA14389	1	1	1	1	NA	NA
LDA14390	1	1	1	1	NA	NA
LDA14391	1	1	0	0	NA	putative:1, secreted:1
LDA15221	1	1	1	1	NA	NA
LDA15349	1	1	1	1	NA	NA
LDA15785	1	1	1	1	NA	NA
LDA16072	1	1	1	1	NA	NA
LDA16073	1	1	1	1	NA	NA
LDA16074	1	1	1	1	NA	NA
LDA16076	1	1	1	1	NA	NA
LDA16078	1	1	1	1	NA	NA
LDA16080	1	1	1	1	NA	NA
LDA16082	1	1	1	1	NA	NA
LDA16085	1	1	1	1	NA	NA
LDA16089	1	1	1	1	NA	NA
LDA16090	1	1	1	1	NA	NA
LDA16202	1	1	1	1	NA	methyltransferase:1
LDA16205	1	1	1	1	NA	oxidoreductase:1
LDA16206	1	1	1	1	NA	NA
LDA16207	1	1	1	1	NA	NA
LDA16208	1	1	1	1	NA	NA
LDA16220	1	1	1	1	NA	hypothetical:1
LDA16337	1	1	1	0	NA	cyclase:1
LDA16612	1	1	1	1	NA	NA
LDA16613	1	1	1	1	NA	NA
LDA16614	1	1	1	1	NA	NA
LDA16615	1	1	1	1	NA	NA
LDA16616	1	1	1	1	NA	NA
LDA16617	1	1	1	1	NA	NA
LDA16618	1	1	1	1	NA	NA
LDA16619	1	1	1	1	NA	glycosyl:1
LDA16620	1	1	1	1	NA	NA
LDA16621	1	1	1	1	NA	NA
LDA16622	1	1	1	1	NA	NA
LDA16623	1	1	1	1	NA	NA
LDA16625	1	1	1	1	NA	NA

LDA16626	1	1	1	1	NA	RNA:1
LDA16627	1	1	1	1	NA	NA
LDA17123	1	1	1	0	NA	dTDP-4-keto-6-deoxy-L-hexose2,3-dehydratase:1
LDA17125	1	1	1	1	NA	NA
LDA17258	1	1	1	0	NA	GTP-binding:1
LDA17511	1	1	1	1	NA	NA
LDA17512	1	1	1	1	NA	NA
LDA17513	1	1	1	1	NA	L-aspartate:1
LDA17514	1	1	1	1	NA	NA
LDA17848	1	1	1	1	NA	hypothetical:1
LDA18477	1	1	1	1	NA	NA
LDA18478	1	1	1	1	NA	NA
LDA18479	1	1	1	1	NA	NA
LDA18480	1	1	1	1	NA	NA
LDA18481	1	1	1	1	NA	NA
LDA18709	1	1	1	1	NA	type:1
LDA18710	1	1	1	1	NA	NA
LDA19180	1	1	1	1	iolD:1	malonic:1
LDA20189	1	1	1	1	NA	NA
LDA20190	1	1	1	1	NA	NA
LDA20192	1	1	1	1	NA	NA
LDA20451	1	1	1	0	NA	hypothetical:1
**Total number of gains**	238	174	152	134		

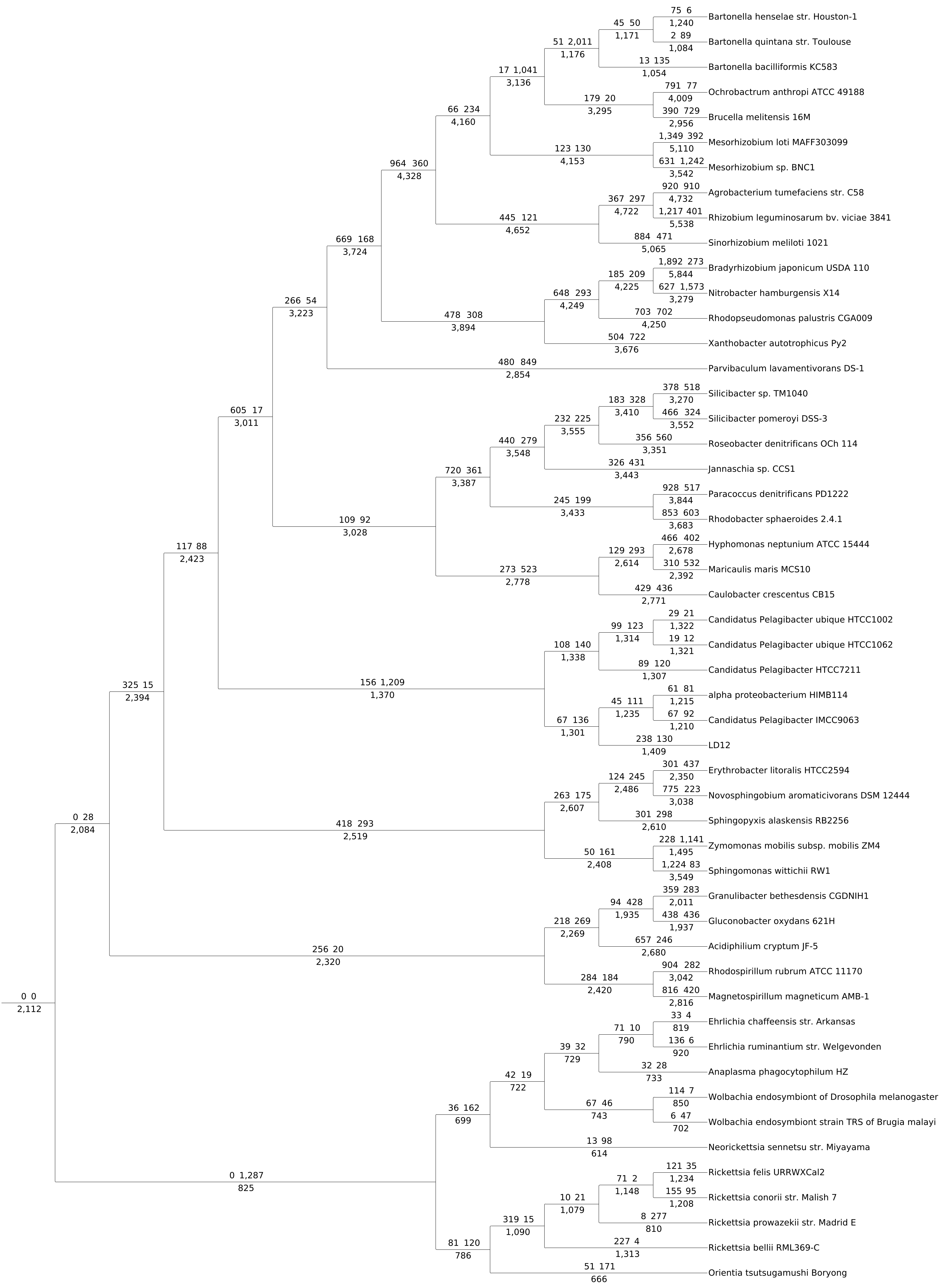
75 6
1,240
Bartonella henselae str. Houston-1
2 89
1,084
Bartonella quintana str. Toulouse
45 50
1,171
51 2,011
1,176
13 135
1,054
Bartonella bacilliformis KC583
17 1,041
3,136
791 77
4,009
Ochrobactrum anthropi ATCC 49188
179 20
3,295
390 729
2,956
Brucella melitensis 16M
66 234
4,160
1,349 392
5,110
Mesorhizobium loti MAFF303099
123 130
4,153
631 1,242
3,542
Mesorhizobium sp. BNC1
964 360
4,328
920 910
4,732
Agrobacterium tumefaciens str. C58
367 297
4,722
1,217 401
5,538
Rhizobium leguminosarum bv. viciae 3841
445 121
4,652
884 471
5,065
Sinorhizobium meliloti 1021
669 168
3,724
1,892 273
5,844
Bradyrhizobium japonicum USDA 110
185 209
4,225
627 1,573
3,279
Nitrobacter hamburgensis X14
648 293
4,249
703 702
4,250
Rhodopseudomonas palustris CGA009
266 54
3,223
478 308
3,894
504 722
3,676
Xanthobacter autotrophicus Py2
480 849
2,854
Parvibaculum lavamentivorans DS-1
378 518
3,270
Silicibacter sp. TM1040
183 328
3,410
466 324
3,552
Silicibacter pomeroyi DSS-3
605 17
3,011
232 225
3,555
356 560
3,351
Roseobacter denitrificans OCh 114
440 279
3,548
326 431
3,443
Jannaschia sp. CCS1
720 361
3,387
928 517
3,844
Paracoccus denitrificans PD1222
245 199
3,433
853 603
3,683
Rhodobacter sphaeroides 2.4.1
109 92
3,028
466 402
2,678
Hyphomonas neptunium ATCC 15444
129 293
2,614
310 532
2,392
Maricaulis maris MCS10
117 88
2,423
273 523
2,778
429 436
2,771
Caulobacter crescentus CB15
29 21
1,322
Candidatus Pelagibacter ubique HTCC1002
99 123
1,314
19 12
1,321
Candidatus Pelagibacter ubique HTCC1062
108 140
1,338
89 120
1,307
Candidatus Pelagibacter HTCC7211
156 1,209
1,370
61 81
1,215
alpha proteobacterium HIMB114
325 15
2,394
45 111
1,235
67 92
1,210
Candidatus Pelagibacter IMCC9063
67 136
1,301
238 130
1,409
LD12
301 437
2,350
Erythrobacter litoralis HTCC2594
124 245
2,486
775 223
3,038
Novosphingobium aromaticivorans DSM 12444
263 175
2,607
301 298
2,610
Sphingopyxis alaskensis RB2256
0 28
2,084
418 293
2,519
228 1,141
1,495
Zymomonas mobilis subsp. mobilis ZM4
50 161
2,408
1,224 83
3,549
Sphingomonas wittichii RW1
359 283
2,011
Granulibacter bethesdensis CGDNIH1
94 428
1,935
438 436
1,937
Gluconobacter oxydans 621H
218 269
2,269
657 246
2,680
Acidiphilium cryptum JF-5
256 20
2,320
904 282
3,042
Rhodospirillum rubrum ATCC 11170
284 184
2,420
816 420
2,816
Magnetospirillum magneticum AMB-1
0 0
2,112
33 4
819
Ehrlichia chaffeensis str. Arkansas
71 10
790
136 6
920
Ehrlichia ruminantium str. Welgevonden
39 32
729
32 28
733
Anaplasma phagocytophilum HZ
42 19
722
114 7
850
Wolbachia endosymbiont of Drosophila melanogaster
36 162
699
67 46
743
6 47
702
Wolbachia endosymbiont strain TRS of Brugia malayi
13 98
614
Neorickettsia sennetsu str. Miyayama
0 1,287
825
121 35
1,234
Rickettsia felis URRWXCal2
71 2
1,148
155 95
1,208
Rickettsia conorii str. Malish 7
10 21
1,079
8 277
810
Rickettsia prowazekii str. Madrid E
319 15
1,090
227 4
1,313
Rickettsia bellii RML369-C
81 120
786
51 171
666
Orientia tsutsugamushi Boryong

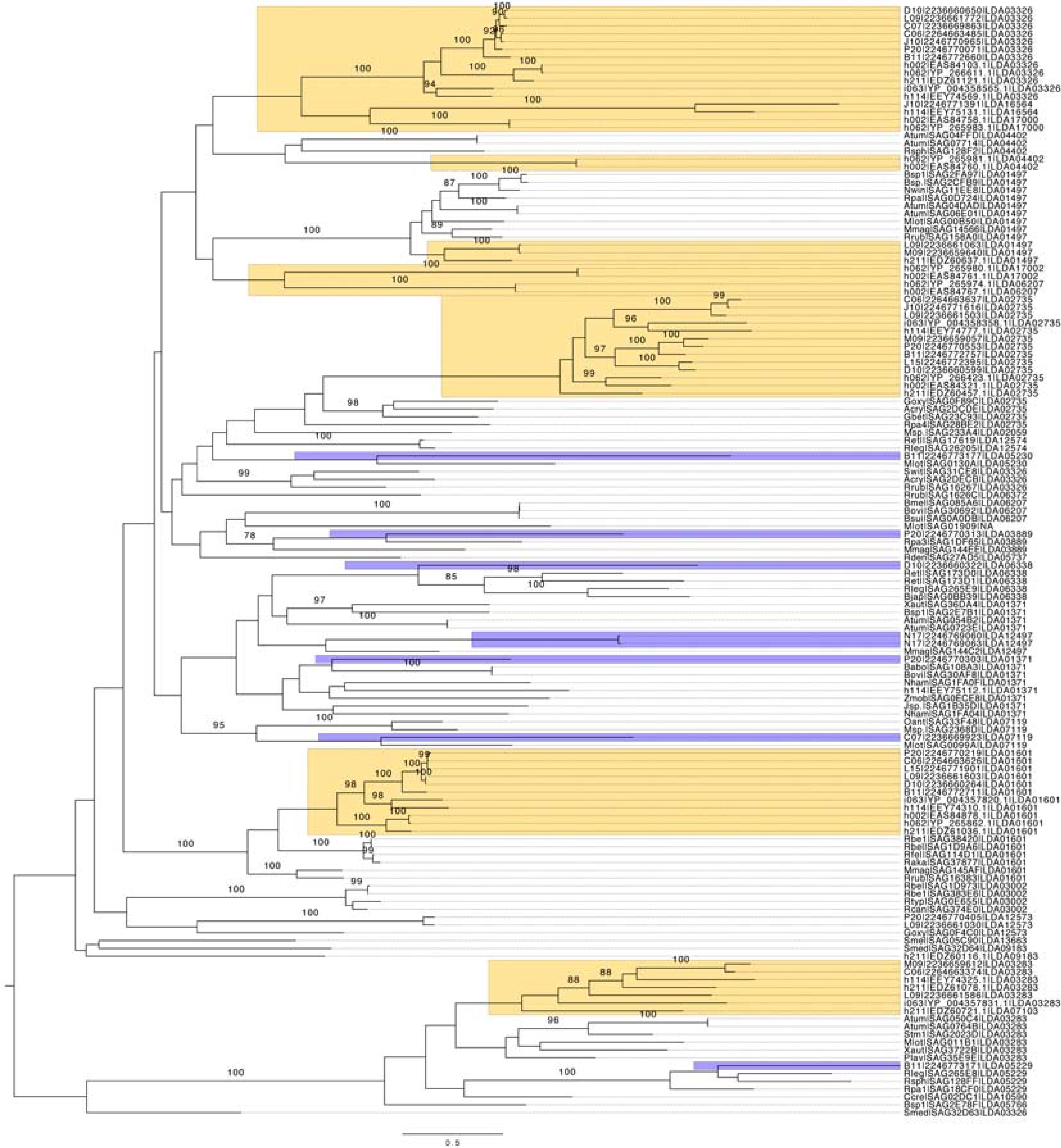
D10|2236660650|LDA03326
L09|2236661772|LDA03326
C07|2236669863|LDA03326
C06|2264663485|LDA03326
J10|2246770965|LDA03326
P20|2246770071|LDA03326
B11|2246772660|LDA03326
h002|EAS84103.1|LDA03326
h062|YP_266611.1|LDA03326
h211|EDZ61121.1|LDA03326
i063|YP_004358565.1|LDA03326
h114|EEY74569.1|LDA03326
J10|2246771391|LDA16564
h114|EEY75131.1|LDA16564
h002|EAS84758.1|LDA17000
h062|YP_265983.1|LDA17000
Atum|SAG04FFD|LDA04402
Atum|SAG07714|LDA04402
Rsph|SAG128F2|LDA04402
h062|YP_265981.1|LDA04402
h002|EAS84760.1|LDA04402
Bsp1|SAG2FA97|LDA01497
Bsp.|SAG2CFB9|LDA01497
Nwin|SAG11EE8|LDA01497
Rpal|SAG0D724|LDA01497
Atum|SAG04DAD|LDA01497
Atum|SAG06E01|LDA01497
Mlot|SAG00B50|LDA01497
Mmag|SAG14566|LDA01497
Rrub|SAG158A0|LDA01497
L09|2236661063|LDA01497
M09|2236659640|LDA01497
h211|EDZ60637.1|LDA01497
h062|YP_265980.1|LDA17002
h002|EAS84761.1|LDA17002
h062|YP_265974.1|LDA06207
h002|EAS84767.1|LDA06207
C06|2264663637|LDA02735
J10|2246771616|LDA02735
L09|2236661503|LDA02735
i063|YP_004358358.1|LDA02735
h114|EEY74777.1|LDA02735
M09|2236659057|LDA02735
P20|2246770553|LDA02735
B11|2246772757|LDA02735
L15|2246772395|LDA02735
D10|2236660599|LDA02735
h062|YP_266423.1|LDA02735
h002|EAS84321.1|LDA02735
h211|EDZ60457.1|LDA02735
Goxy|SAG0F89C|LDA02735
Acry|SAG2DC0E|LDA02735
Gbet|SAG23C93|LDA02735
Rpa4|SAG28BE2|LDA02735
Msp.|SAG233A4|LDA02059
Retl|SAG17619|LDA12574
Rleg|SAG26205|LDA12574
B11|2246773177|LDA05230
Mlot|SAG0130A|LDA05230
Swit|SAG31CE8|LDA03326
Acry|SAG2DECB|LDA03326
Rrub|SAG16267|LDA03326
Rrub|SAG1626C|LDA06372
Bmel|SAG085A6|LDA06207
Bovi|SAG30692|LDA06207
Bsui|SAG0A0D8|LDA06207
Mlot|SAG01909|NA
P20|2246770313|LDA03889
Rpa3|SAG1DF65|LDA03889
Mmag|SAG144EE|LDA03889
Rden|SAG27AD5|LDA05737
D10|2236660322|LDA06338
Retl|SAG17300|LDA06338
Retl|SAG17301|LDA06338
Rleg|SAG265E9|LDA06338
Bjap|SAG0BB39|LDA06338
Xaut|SAG36DA4|LDA01371
Bsp1|SAG2E7B1|LDA01371
Atum|SAG054B2|LDA01371
Atum|SAG0723F|LDA01371
N17|2246769060|LDA12497
N17|2246769063|LDA12497
Mmag|SAG144C2|LDA12497
P20|2246770303|LDA01371
Babo|SAG108A3|LDA01371
Bovi|SAG30AF8|LDA01371
Nham|SAG1FA0F|LDA01371
h114|EEY75112.1|LDA01371
Zmob|SAG0ECE8|LDA01371
Jsp.|SAG1B35D|LDA01371
Nham|SAG1FA04|LDA01371
Oant|SAG33F48|LDA07119
Msp.|SAG2368D|LDA07119
C07|2236669923|LDA07119
Mlot|SAG0099A|LDA07119
P20|2246770219|LDA01601
C06|2264663626|LDA01601
L15|2246771901|LDA01601
L09|2236661603|LDA01601
D10|2236660264|LDA01601
B11|2246772711|LDA01601
i063|YP_004357820.1|LDA01601
h114|EEY74310.1|LDA01601
h002|EAS84878.1|LDA01601
h062|YP_265862.1|LDA01601
h211|EDZ61036.1|LDA01601
Rbe1|SAG38420|LDA01601
Rbel|SAG1D9A6|LDA01601
Rfel|SAG114D1|LDA01601
Raka|SAG37877|LDA01601
Mmag|SAG145AF|LDA01601
Rrub|SAG16383|LDA01601
Rbel|SAG1D973|LDA03002
Rbe1|SAG383E6|LDA03002
Rtyp|SAG0E655|LDA03002
Rcan|SAG374E0|LDA03002
P20|2246770405|LDA12573
L09|2236661030|LDA12573
Goxy|SAG0F4C0|LDA12573
Smel|SAG05C90|LDA13663
Smed|SAG32D64|LDA09183
h211|EDZ60116.1|LDA09183
M09|2236659612|LDA03283
C06|2264663374|LDA03283
h114|EEY74325.1|LDA03283
h211|EDZ61078.1|LDA03283
L09|2236661586|LDA03283
i063|YP_004357831.1|LDA03283
h211|EDZ60721.1|LDA07103
Atum|SAG050C4|LDA03283
Atum|SAG0764B|LDA03283
Stm1|SAG2023D|LDA03283
Mlot|SAG011B1|LDA03283
Xaut|SAG3722B|LDA03283
Plav|SAG35E9E|LDA03283
B11|2246773171|LDA05229
Rleg|SAG265E8|LDA05229
Rsph|SAG128FF|LDA05229
Rpa1|SAG18CF0|LDA05229
Ccre|SAG02DC1|LDA10590
Bsp1|SAG2E78F|LDA05768
Smed|SAG32D63|LDA03326
0.5

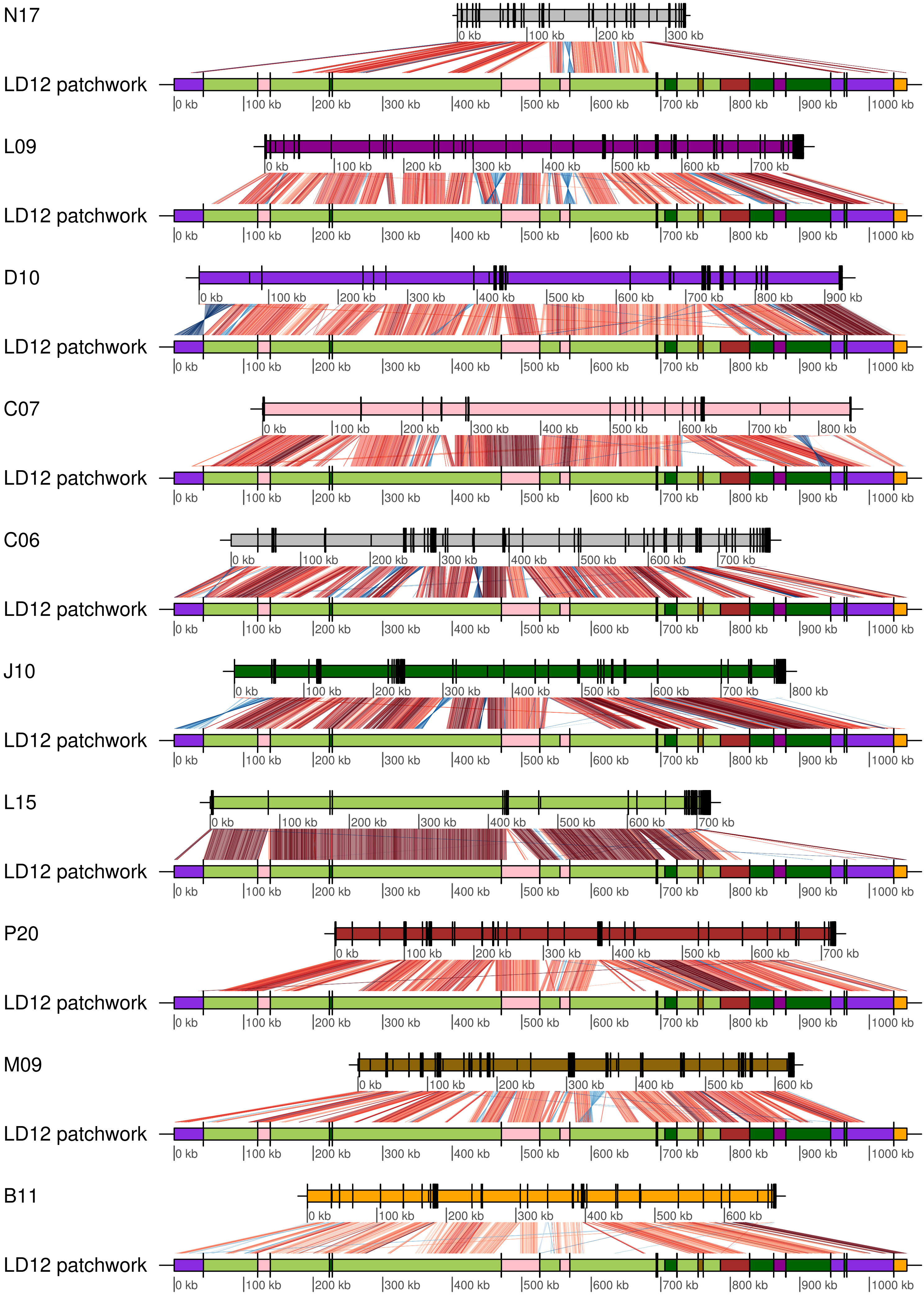

N17
0 kb 100 kb 200 kb 300 kb
LD12 patchwork
0 kb 100 kb 200 kb 300 kb 400 kb 500 kb 600 kb 700 kb 800 kb 900 kb 1000 kb
L09
0 kb 100 kb 200 kb 300 kb 400 kb 500 kb 600 kb 700 kb
LD12 patchwork
D10
0 kb 100 kb 200 kb 300 kb 400 kb 500 kb 600 kb 700 kb 800 kb 900 kb
LD12 patchwork
C07
0 kb 100 kb 200 kb 300 kb 400 kb 500 kb 600 kb 700 kb 800 kb
LD12 patchwork
C06
0 kb 100 kb 200 kb 300 kb 400 kb 500 kb 600 kb 700 kb
LD12 patchwork
J10
0 kb 100 kb 200 kb 300 kb 400 kb 500 kb 600 kb 700 kb 800 kb
LD12 patchwork
L15
0 kb 100 kb 200 kb 300 kb 400 kb 500 kb 600 kb 700 kb
LD12 patchwork
P20
0 kb 100 kb 200 kb 300 kb 400 kb 500 kb 600 kb 700 kb
LD12 patchwork
M09
0 kb 100 kb 200 kb 300 kb 400 kb 500 kb 600 kb
LD12 patchwork
B11
0 kb 100 kb 200 kb 300 kb 400 kb 500 kb 600 kb
LD12 patchwork

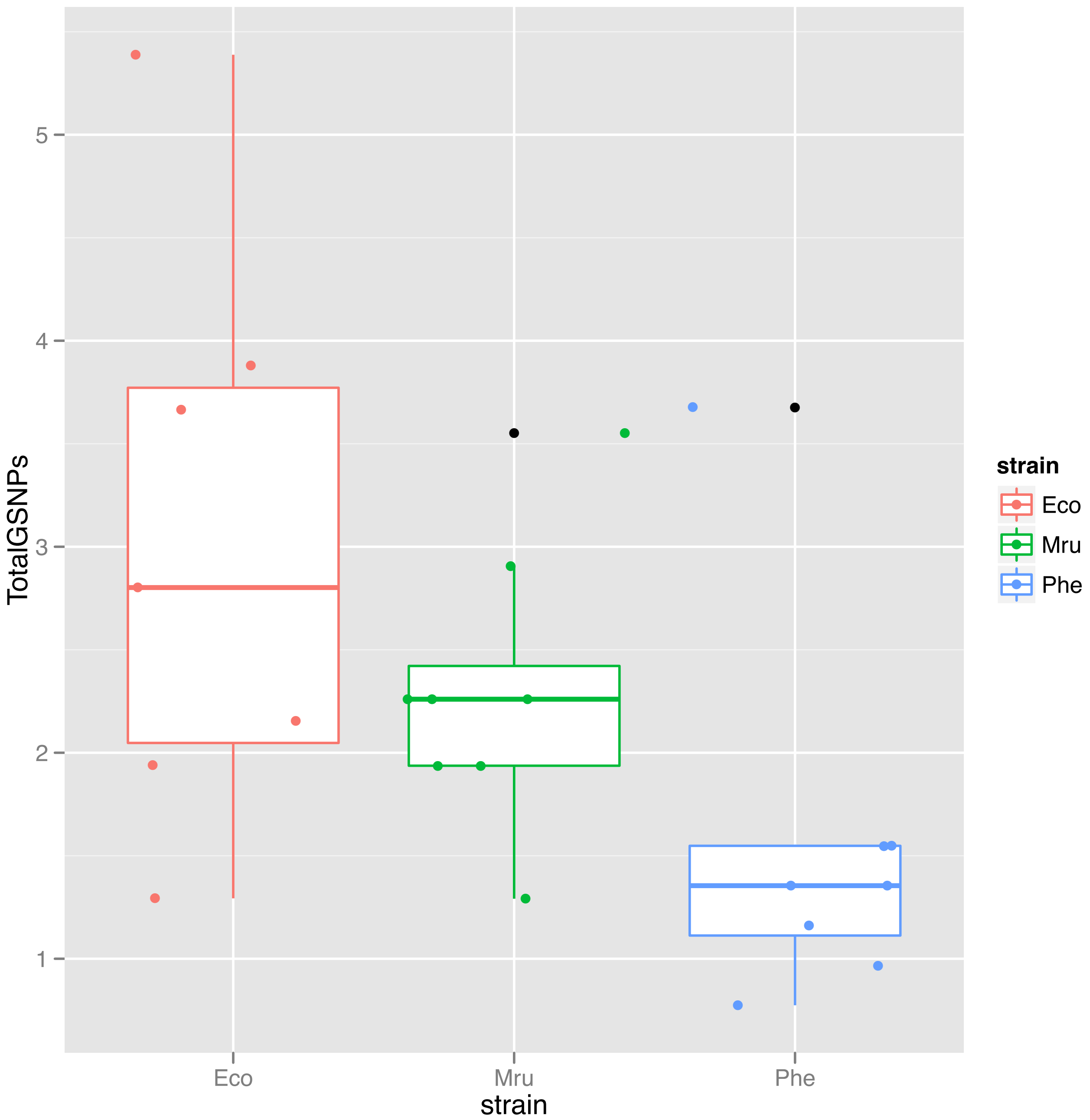

TotalGSNPs
5
4
3
2
1
Eco
Mru
Phe
strain
strain
Eco
Mru
Phe

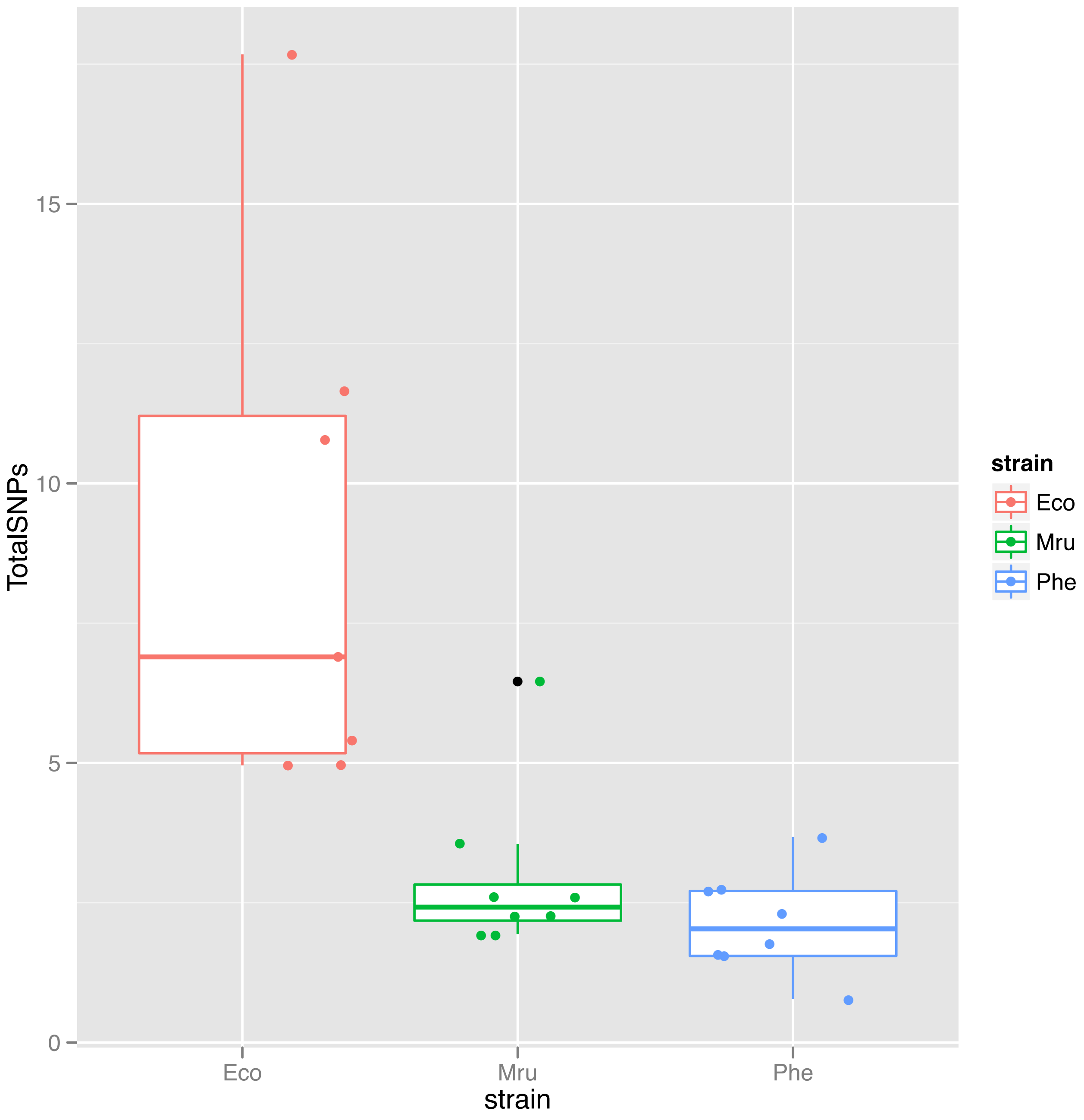

TotalSNPs
15
10
5
0
Eco
Mru
Phe
strain
strain
Eco
Mru
Phe

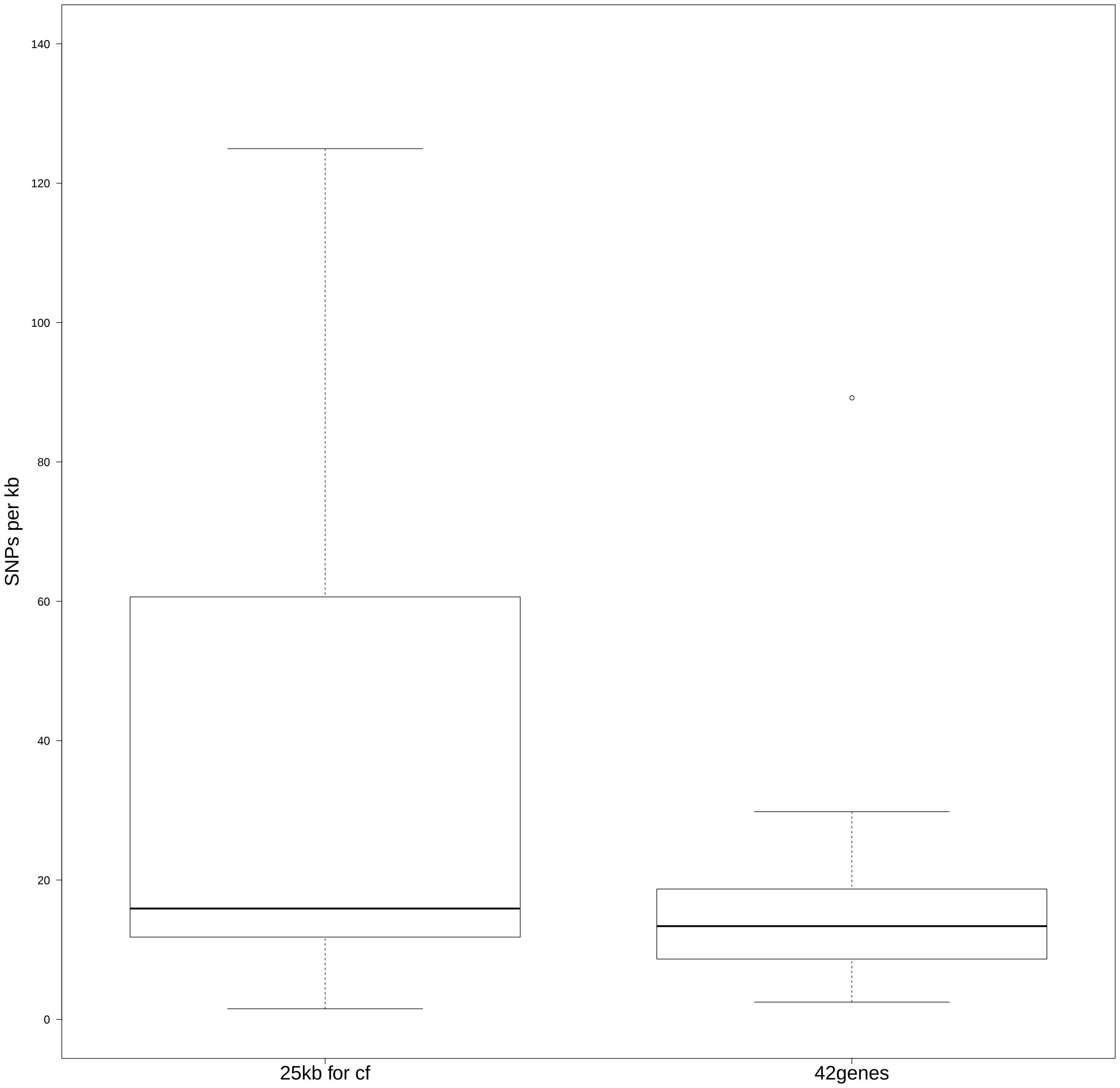

140
120
100
80
60
40
20
0
SNPs per kb
25kb for cf
42genes
