# Supplementary material for: Single-cell genomics reveal low recombination frequencies in freshwater bacteria of the SAR11 clade
Source: Genome Biol. 2013 Nov 28;14(11):R130. doi: 10.1186/gb-2013-14-11-r130 (PMC4053759; doi:10.1186/gb-2013-14-11-r130)

00003 – groEL

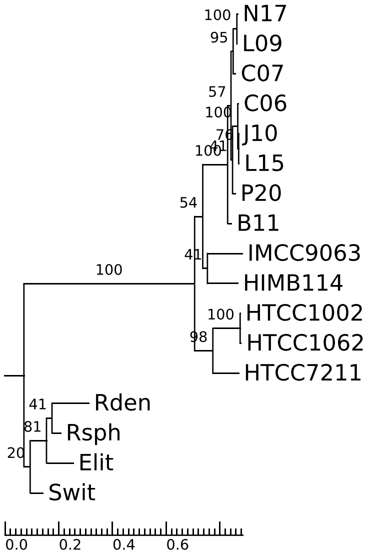

00013 – ftsK

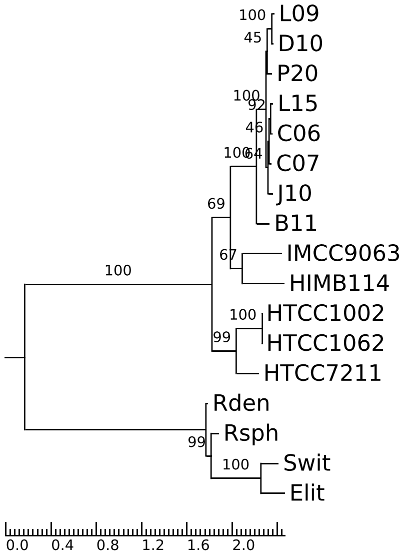

00033 – rho

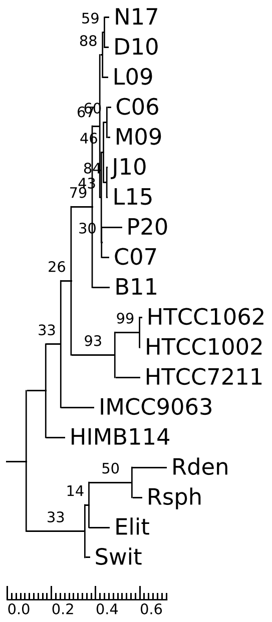

00045 – tolC

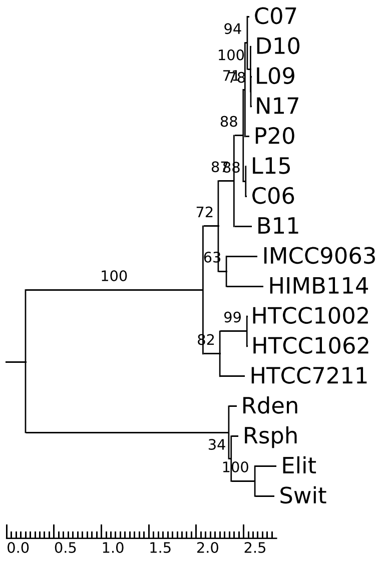

00071 – pnp

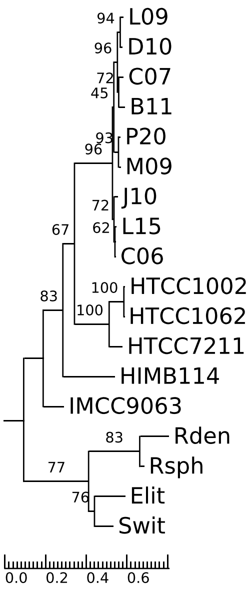

00073 – infB

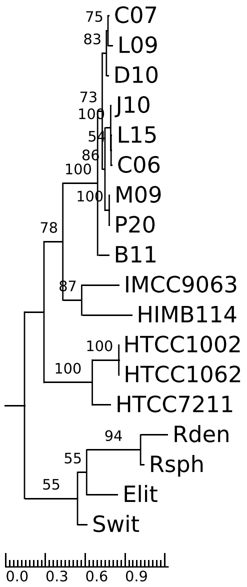

00074 – nusA

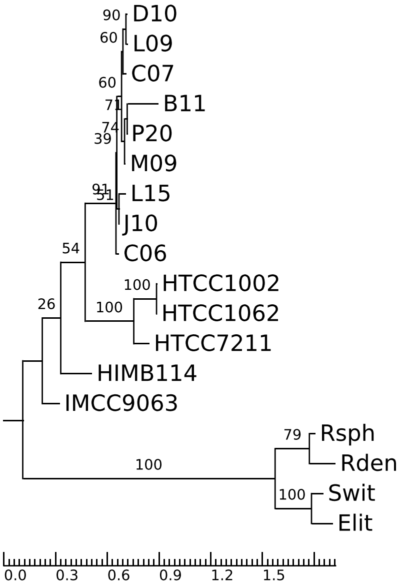

00081 – mgtE

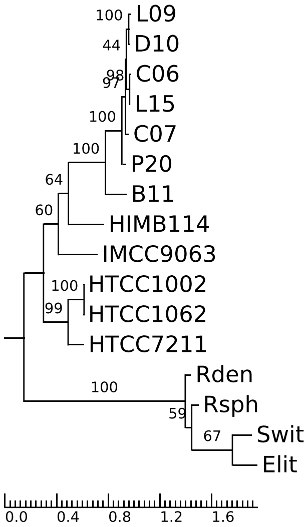

00085 – ligA

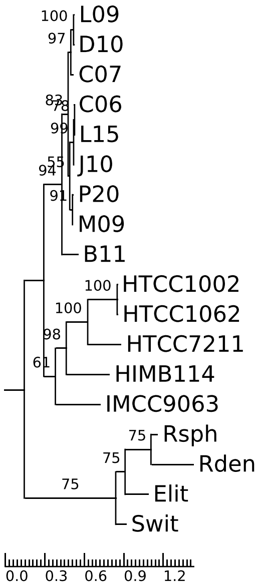

00086

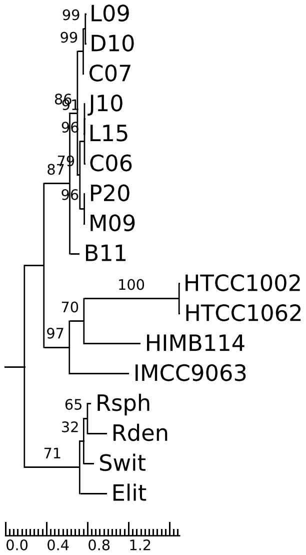

00092 – sucC

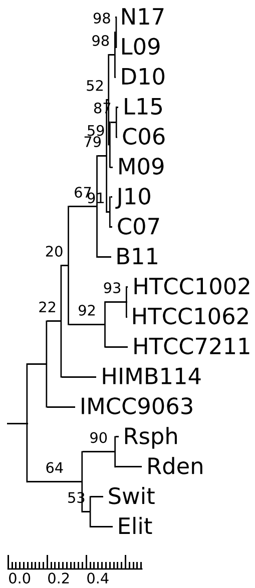

00093 – leuS

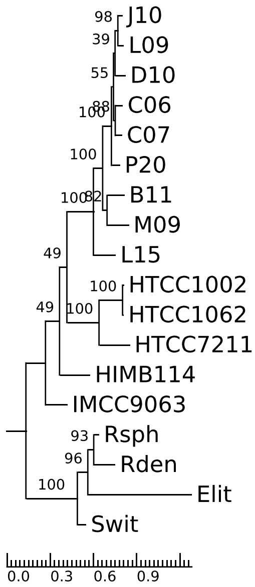

00094 – gidA

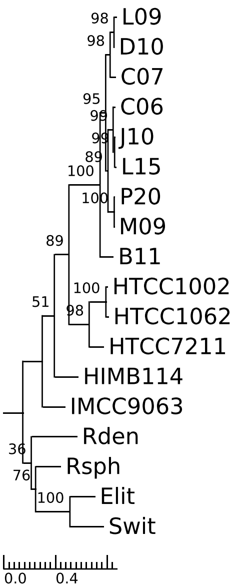

00095 – trmE

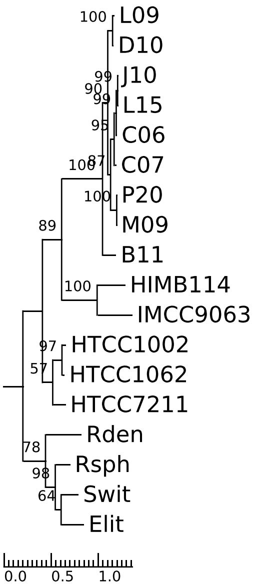

00102 – dnaN

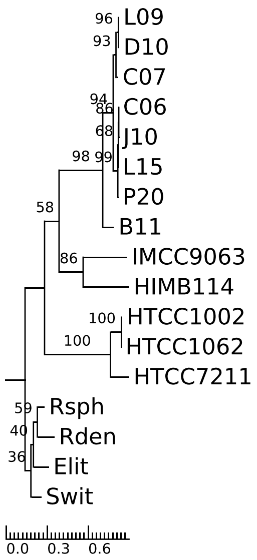

00104 – dnaA

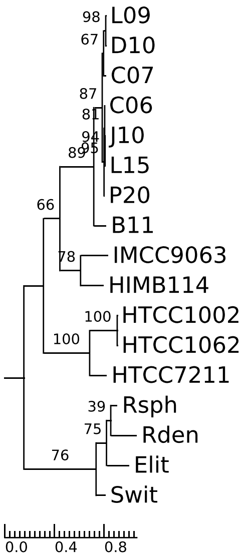

00122 – recA

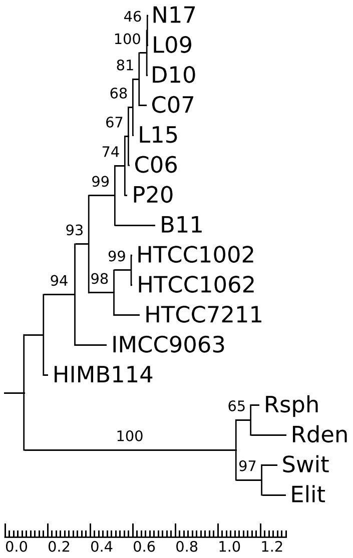

00127 – atpD

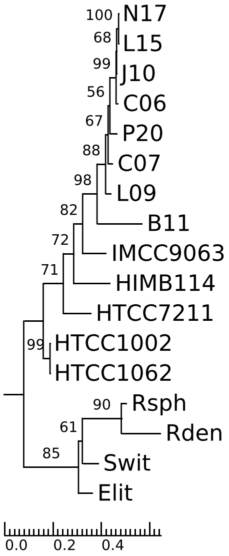

00130 – ubiH

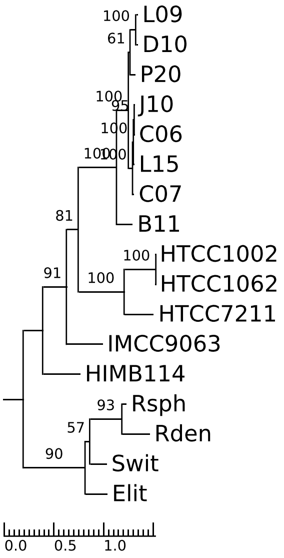

00143 – dapE

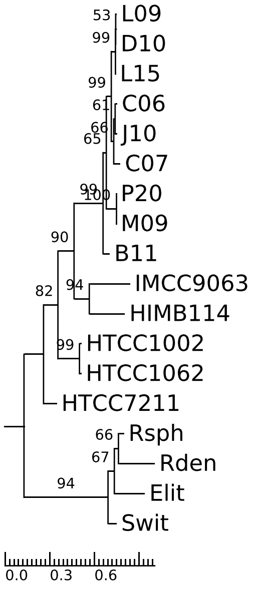

00162 – recJ

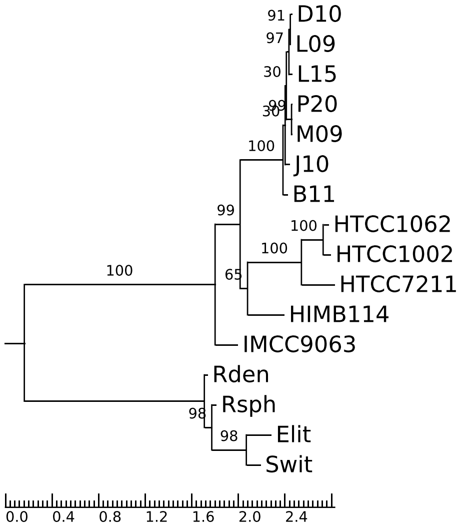

00168 – rpoA

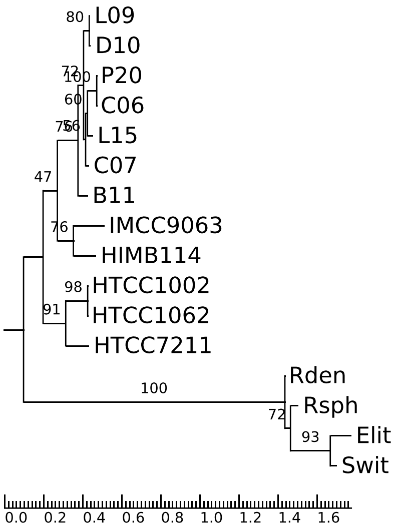

00177

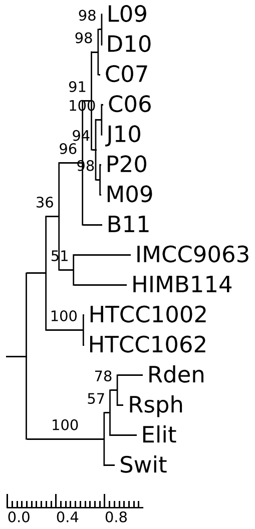

00183 – priA

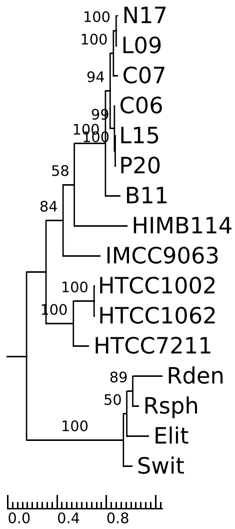

00186 – gcp

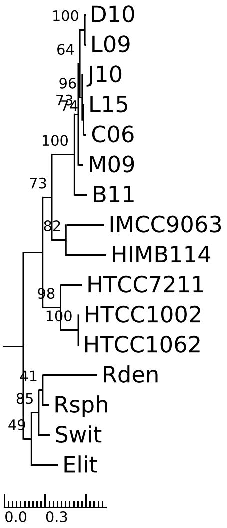

00205 – pheS

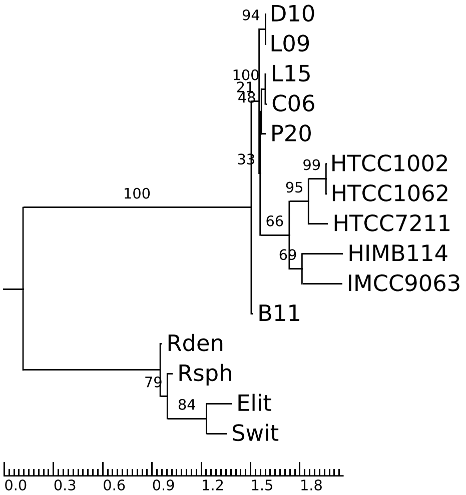

00206 – pheT

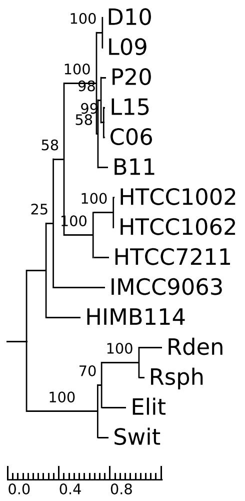

00227 – thrS

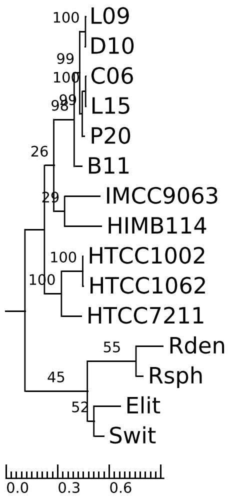

00247 – hemE

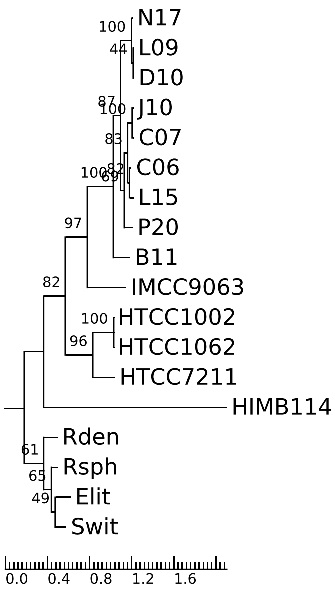

00257 – ubiB

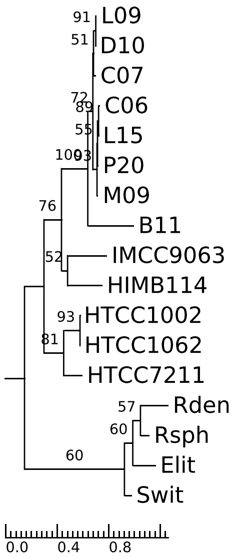

00294

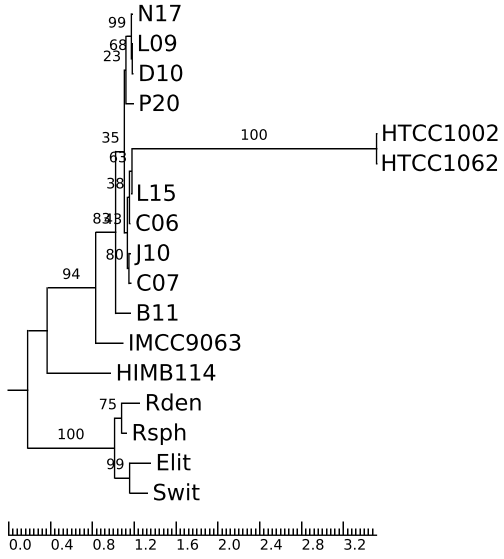

00398 – murC

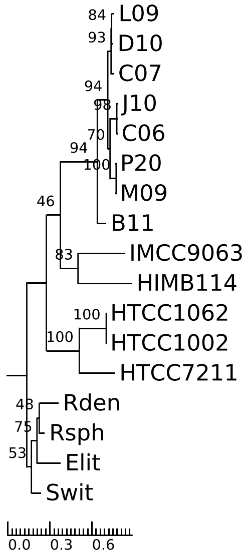

00399 – murG

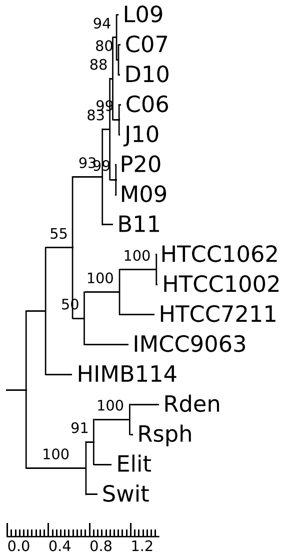

00409 – coaBC

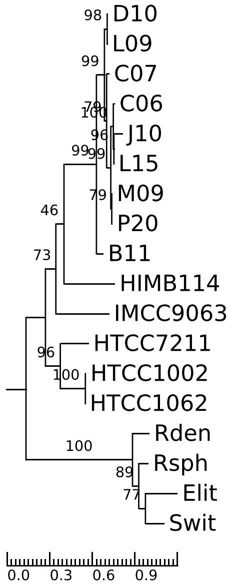

00442 – purH

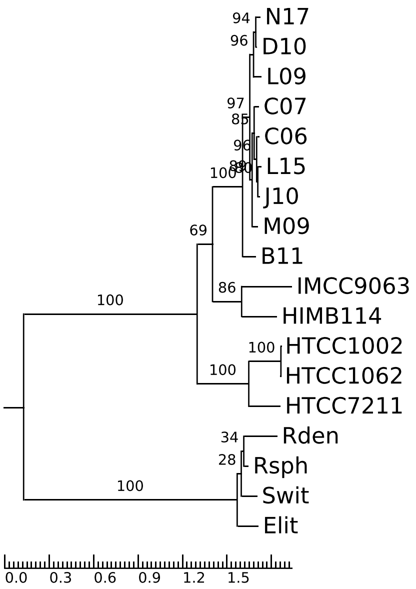

00468 – abcT2

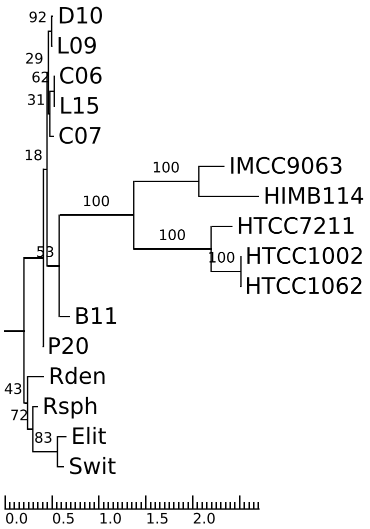

00485 – purA

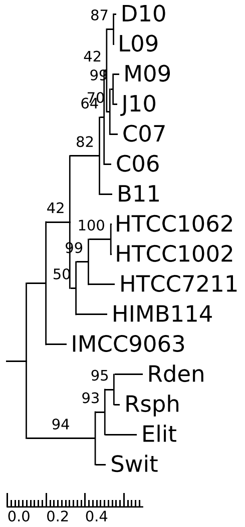

00509 – acsA

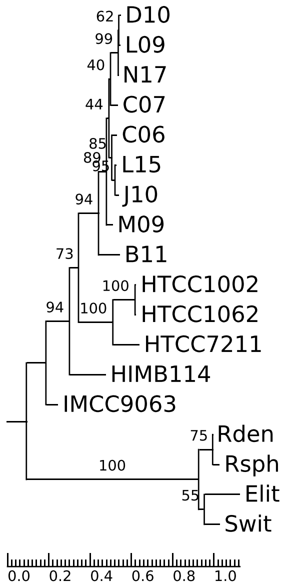

00515 – pmbA

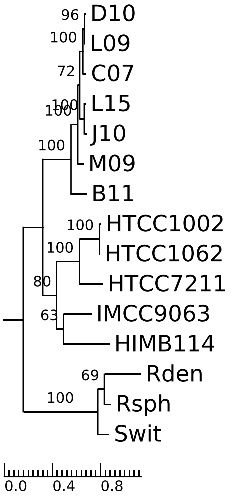

00556 – dacF

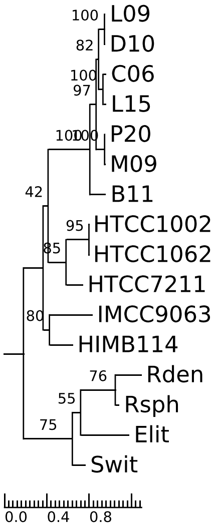

00588 – argG

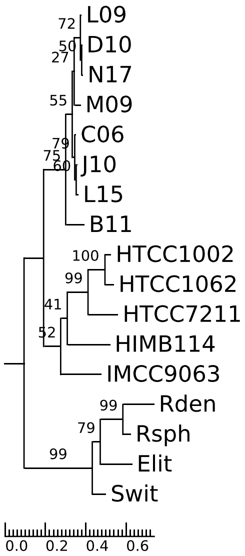

00694 – mutY

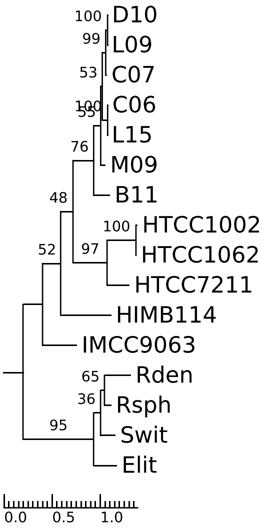

Supplement: Additional file 2 — Phylogenetic analysis of 42 genes from the LD12 SAGs. A phylogenetic inference of genes from the LD12 SAGs. Abbreviations of SAGs show microcluster and the name and cluster number from Integrated Microbial Genomes (IMG). [file gb-2013-14-11-r130-S2.pdf]
