# Supplementary material for: Single-cell genomics reveal low recombination frequencies in freshwater bacteria of the SAR11 clade
Source: Genome Biol. 2013 Nov 28;14(11):R130. doi: 10.1186/gb-2013-14-11-r130 (PMC4053759; doi:10.1186/gb-2013-14-11-r130)
Supplement: Additional file 3 — Phylogenetic analysis of 42 genes from the LD12 SAGs and recruited metagenome sequences from six lakes. A phylogenetic inference of genes from the LD12 SAGs and affiliated genes obtained from the metagenomic dataset. Abbreviations of SAGs show microcluster, and the name and cluster number from Integrated Microbial Genomes (IMG). Abbreviations of metagenomic sequences indicate the lake and the sampling season. SSu = Sparkling summer (green); DSu and DSp = Damariscotta summer and spring (red); MSu and MSp = Mendota summer and spring (blue); Erk = Erken; Eko = Ekoln; Vat = Vättern. The marine SAR11 strains were used as outgroups (not shown). [file gb-2013-14-11-r130-S3.pdf]

3 groEL

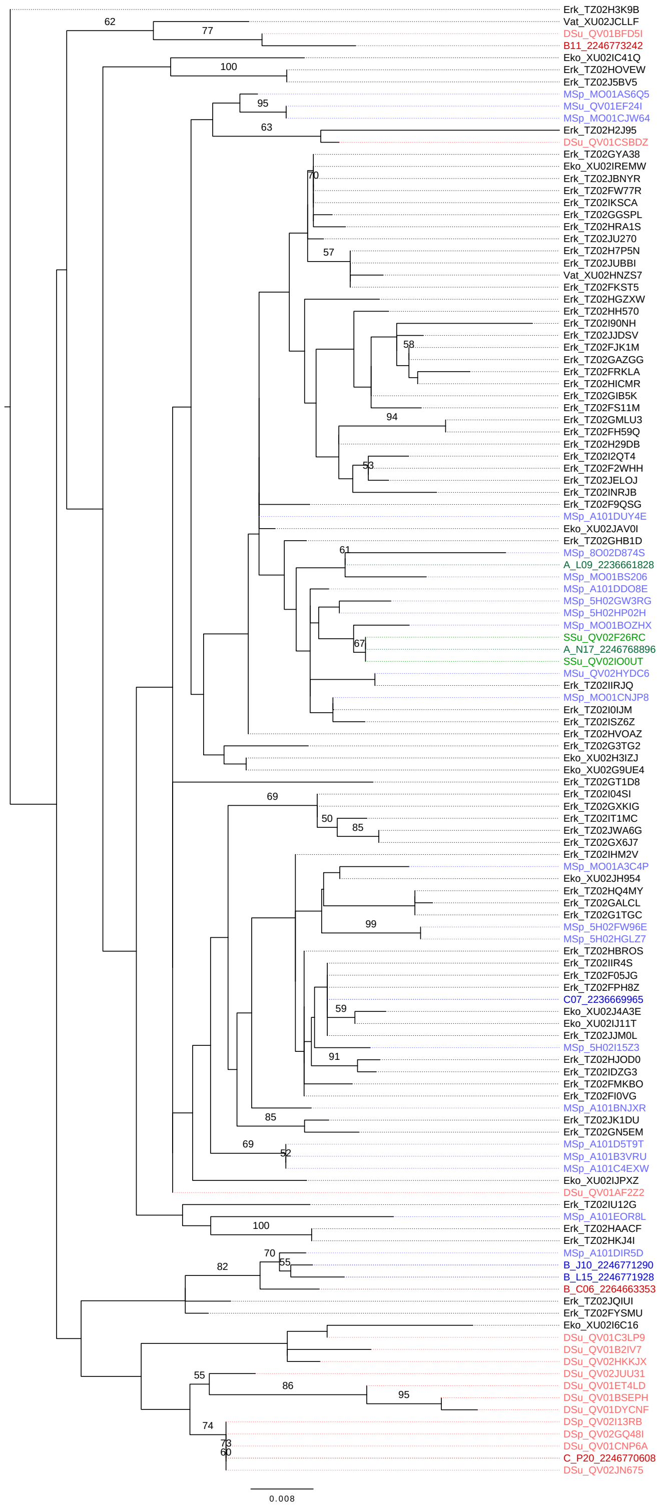
Erk_TZ02H3K9B
Vat_XU02JCLLF
DSu_QV01BFD5I
B11_2246773242
Eko_XU02IC41Q
Erk_TZ02HOVEW
Erk_TZ02J5BV5
MSp_MO01AS6Q5
MSu_QV01EF24I
MSp_MO01CJW64
Erk_TZ02H2J95
DSu_QV01CSBDZ
Erk_TZ02GYA38
Eko_XU02IREMW
Erk_TZ02JBNYR
Erk_TZ02FW77R
Erk_TZ02IKSCA
Erk_TZ02GGSPL
Erk_TZ02HRA1S
Erk_TZ02JU270
Erk_TZ02H7P5N
Erk_TZ02JUBBI
Vat_XU02HNZS7
Erk_TZ02FKST5
Erk_TZ02HGZXW
Erk_TZ02HH570
Erk_TZ02I90NH
Erk_TZ02JJDSV
Erk_TZ02FJK1M
Erk_TZ02GAZGG
Erk_TZ02FRKLA
Erk_TZ02HICMR
Erk_TZ02GIB5K
Erk_TZ02FS11M
Erk_TZ02GMLU3
Erk_TZ02FH59Q
Erk_TZ02H29DB
Erk_TZ02I2QT4
Erk_TZ02F2WHH
Erk_TZ02JELOJ
Erk_TZ02INRJB
Erk_TZ02F9QSG
MSp_A101DUY4E
Eko_XU02JAV0I
Erk_TZ02GHB1D
MSp_8O02D874S
A_L09_2236661828
MSp_MO01BS206
MSp_A101DDO8E
MSp_5H02GW3RG
MSp_5H02HP02H
MSp_MO01BOZHX
SSu_QV02F26RC
A_N17_2246768896
SSu_QV02IO0UT
MSu_QV02HYDC6
Erk_TZ02IIRJQ
MSp_MO01CNJP8
Erk_TZ02I0IJM
Erk_TZ02ISZ6Z
Erk_TZ02HVOAZ
Erk_TZ02G3TG2
Eko_XU02H3IZJ
Eko_XU02G9UE4
Erk_TZ02GT1D8
Erk_TZ02I04SI
Erk_TZ02GXKIG
Erk_TZ02IT1MC
Erk_TZ02JWA6G
Erk_TZ02GX6J7
Erk_TZ02IHM2V
MSp_MO01A3C4P
Eko_XU02JH954
Erk_TZ02HQ4MY
Erk_TZ02GALCL
Erk_TZ02G1TGC
MSp_5H02FW96E
MSp_5H02HGLZ7
Erk_TZ02HBROS
Erk_TZ02IIR4S
Erk_TZ02F05JG
Erk_TZ02FPH8Z
C07_2236669965
Eko_XU02J4A3E
Eko_XU02IJ11T
Erk_TZ02JJM0L
MSp_5H02I15Z3
Erk_TZ02HJOD0
Erk_TZ02IDZG3
Erk_TZ02FMKBO
Erk_TZ02FI0VG
MSp_A101BNJXR
Erk_TZ02JK1DU
Erk_TZ02GN5EM
MSp_A101D5T9T
MSp_A101B3VRU
MSp_A101C4EXW
Eko_XU02IJPXZ
DSu_QV01AF2Z2
Erk_TZ02IU12G
MSp_A101EOR8L
Erk_TZ02HAACF
Erk_TZ02HKJ4I
MSp_A101DIR5D
B_J10_2246771290
B_L15_2246771928
B_C06_2264663353
Erk_TZ02JQIUI
Erk_TZ02FYSMU
Eko_XU02I6C16
DSu_QV01C3LP9
DSu_QV01B2IV7
DSu_QV02HKKJX
DSu_QV02JUU31
DSu_QV01ET4LD
DSu_QV01BSEPH
DSu_QV01DYCNF
DSp_QV02I13RB
DSp_QV02GQ48I
DSu_QV01CNP6A
C_P20_2246770608
DSu_QV02JN675
0.008

143 dapE

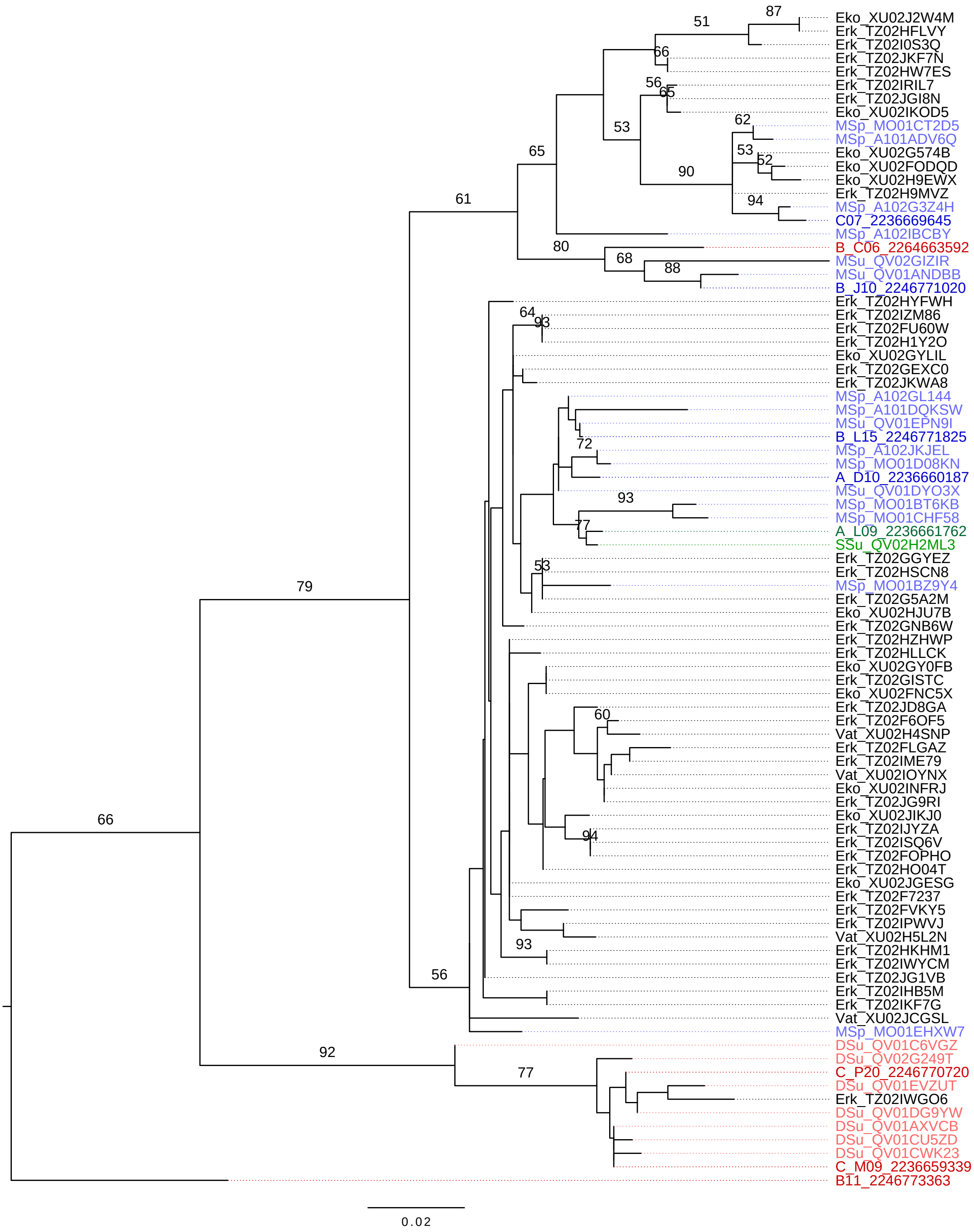
Eko_XU02J2W4M
Erk_TZ02HFLVY
Erk_TZ02I0S3Q
Erk_TZ02JKF7N
Erk_TZ02HW7ES
Erk_TZ02IRIL7
Erk_TZ02JGI8N
Eko_XU02IKOD5
MSp_MO01CT2D5
MSp_A101ADV6Q
Eko_XU02G574B
Eko_XU02FODQD
Eko_XU02H9EWX
Erk_TZ02H9MVZ
MSp_A102G3Z4H
C07_2236669645
MSp_A102IBCBY
B_C06_2264663592
MSu_QV02GIZIR
MSu_QV01ANDBB
B_J10_2246771020
Erk_TZ02HYFWH
Erk_TZ02IZM86
Erk_TZ02FU60W
Erk_TZ02H1Y2O
Eko_XU02GYLIL
Erk_TZ02GEXC0
Erk_TZ02JKWA8
MSp_A102GL144
MSp_A101DQKSW
MSu_QV01EPN9I
B_L15_2246771825
MSp_A102JKJEL
MSp_MO01D08KN
A_D10_2236660187
MSu_QV01DYO3X
MSp_MO01BT6KB
MSp_MO01CHF58
A_L09_2236661762
SSu_QV02H2ML3
Erk_TZ02GGYEZ
Erk_TZ02HSCN8
MSp_MO01BZ9Y4
Erk_TZ02G5A2M
Eko_XU02HJU7B
Erk_TZ02GNB6W
Erk_TZ02HZHWP
Erk_TZ02HLLCK
Eko_XU02GY0FB
Erk_TZ02GISTC
Eko_XU02FNC5X
Erk_TZ02JD8GA
Erk_TZ02F6OF5
Vat_XU02H4SNP
Erk_TZ02FLGAZ
Erk_TZ02IME79
Vat_XU02IOYNX
Eko_XU02INFRJ
Erk_TZ02JG9RI
Eko_XU02JIKJ0
Erk_TZ02IJYZA
Erk_TZ02ISQ6V
Erk_TZ02FOPHO
Erk_TZ02HO04T
Eko_XU02JGESG
Erk_TZ02F7237
Erk_TZ02FVKY5
Erk_TZ02IPWVJ
Vat_XU02H5L2N
Erk_TZ02HKHM1
Erk_TZ02IWYCM
Erk_TZ02JG1VB
Erk_TZ02IHB5M
Erk_TZ02IKF7G
Vat_XU02JCGSL
MSp_MO01EHXW7
DSu_QV01C6VGZ
DSu_QV02G249T
C_P20_2246770720
DSu_QV01EVZUT
Erk_TZ02IWGO6
DSu_QV01DG9YW
DSu_QV01AXVCB
DSu_QV01CU5ZD
DSu_QV01CWK23
C_M09_2236659339
B11_2246773363
0.02

183 priA

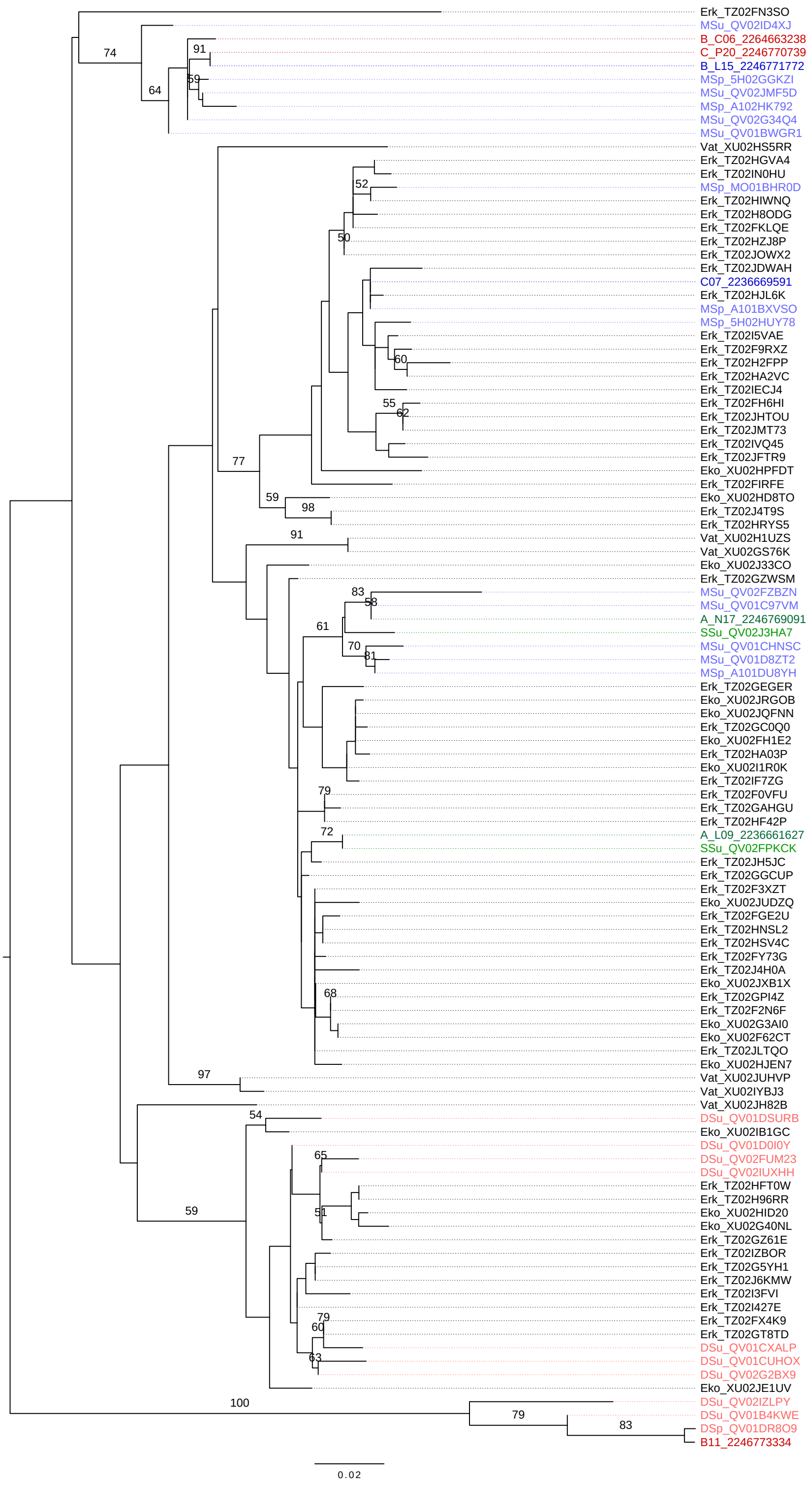

Erk_TZ02FN3SO
MSu_QV02ID4XJ
B_C06_2264663238
C_P20_2246770739
B_L15_2246771772
MSp_5H02GGKZI
MSu_QV02JMF5D
MSp_A102HK792
MSu_QV02G34Q4
MSu_QV01BWGR1
Vat_XU02HS5RR
Erk_TZ02HGVA4
Erk_TZ02IN0HU
MSp_MO01BHR0D
Erk_TZ02HIWNQ
Erk_TZ02H8ODG
Erk_TZ02FKLQE
Erk_TZ02HZJ8P
Erk_TZ02JOWX2
Erk_TZ02JDWAH
C07_2236669591
Erk_TZ02HJL6K
MSp_A101BXVSO
MSp_5H02HUY78
Erk_TZ02I5VAE
Erk_TZ02F9RXZ
Erk_TZ02H2FPP
Erk_TZ02HA2VC
Erk_TZ02IECJ4
Erk_TZ02FH6HI
Erk_TZ02JHTOU
Erk_TZ02JMT73
Erk_TZ02IVQ45
Erk_TZ02JFTR9
Eko_XU02HPFDT
Erk_TZ02FIRFE
Eko_XU02HD8TO
Erk_TZ02J4T9S
Erk_TZ02HRYS5
Vat_XU02H1UZS
Vat_XU02GS76K
Eko_XU02J33CO
Erk_TZ02GZWSM
MSu_QV02FZBZN
MSu_QV01C97VM
A_N17_2246769091
SSu_QV02J3HA7
MSu_QV01CHNSC
MSu_QV01D8ZT2
MSp_A101DU8YH
Erk_TZ02GEGER
Eko_XU02JRGOB
Eko_XU02JQFNN
Erk_TZ02GC0Q0
Eko_XU02FH1E2
Erk_TZ02HA03P
Eko_XU02I1R0K
Erk_TZ02IF7ZG
Erk_TZ02F0VFU
Erk_TZ02GAHGU
Erk_TZ02HF42P
A_L09_2236661627
SSu_QV02FPKCK
Erk_TZ02JH5JC
Erk_TZ02GGCUP
Erk_TZ02F3XZT
Eko_XU02JUDZQ
Erk_TZ02FGE2U
Erk_TZ02HNSL2
Erk_TZ02HSV4C
Erk_TZ02FY73G
Erk_TZ02J4H0A
Eko_XU02JXB1X
Erk_TZ02GPI4Z
Erk_TZ02F2N6F
Eko_XU02G3AI0
Eko_XU02F62CT
Erk_TZ02JLTQO
Eko_XU02HJEN7
Vat_XU02JUHVP
Vat_XU02IYBJ3
Vat_XU02JH82B
DSu_QV01DSURB
Eko_XU02IB1GC
DSu_QV01D0I0Y
DSu_QV02FUM23
DSu_QV02IUXHH
Erk_TZ02HFT0W
Erk_TZ02H96RR
Eko_XU02HID20
Eko_XU02G40NL
Erk_TZ02GZ61E
Erk_TZ02IZBOR
Erk_TZ02G5YH1
Erk_TZ02J6KMW
Erk_TZ02I3FVI
Erk_TZ02I427E
Erk_TZ02FX4K9
Erk_TZ02GT8TD
DSu_QV01CXALP
DSu_QV01CUHOX
DSu_QV02G2BX9
Eko_XU02JE1UV
DSu_QV02IZLPY
DSu_QV01B4KWE
DSp_QV01DR8O9
B11_2246773334
0.02

442 purH

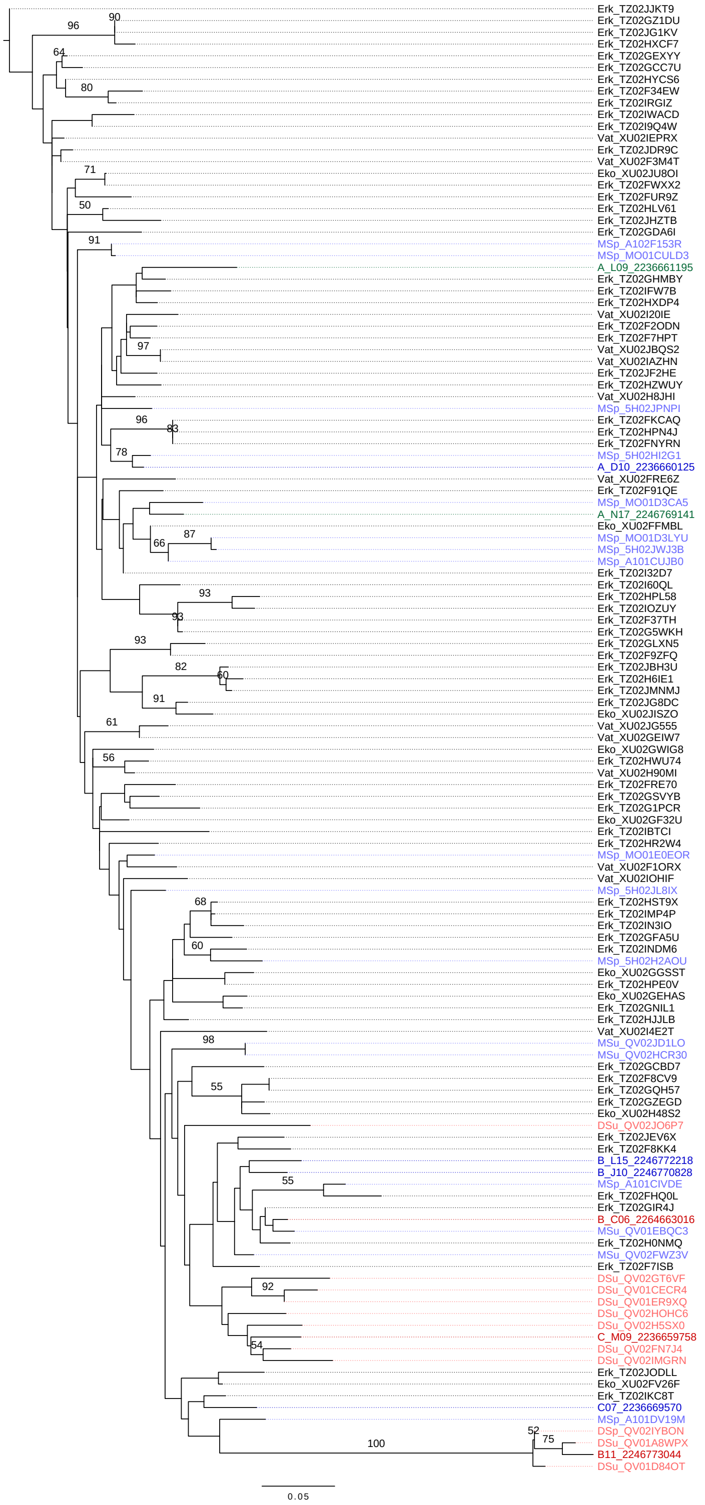
Erk_TZ02JJKT9
Erk_TZ02GZ1DU
Erk_TZ02JG1KV
Erk_TZ02HXCF7
Erk_TZ02GEXYY
Erk_TZ02GCC7U
Erk_TZ02HYCS6
Erk_TZ02F34EW
Erk_TZ02IRGIZ
Erk_TZ02IWACD
Erk_TZ02I9Q4W
Vat_XU02IEPRX
Erk_TZ02JDR9C
Vat_XU02F3M4T
Eko_XU02JU8OI
Erk_TZ02FWXX2
Erk_TZ02FUR9Z
Erk_TZ02HLV61
Erk_TZ02JHZTB
Erk_TZ02GDA6I
MSp_A102F153R
MSp_MO01CULD3
A_L09_2236661195
Erk_TZ02GHMBY
Erk_TZ02IFW7B
Erk_TZ02HXDP4
Vat_XU02I20IE
Erk_TZ02F2ODN
Erk_TZ02F7HPT
Vat_XU02JBQS2
Vat_XU02IAZHN
Erk_TZ02JF2HE
Erk_TZ02HZWUY
Vat_XU02H8JHI
MSp_5H02JPNPI
Erk_TZ02FKCAQ
Erk_TZ02HPN4J
Erk_TZ02FNYRN
MSp_5H02HI2G1
A_D10_2236660125
Vat_XU02FRE6Z
Erk_TZ02F91QE
MSp_MO01D3CA5
A_N17_2246769141
Eko_XU02FFMBL
MSp_MO01D3LYU
MSp_5H02JWJ3B
MSp_A101CUJB0
Erk_TZ02I32D7
Erk_TZ02I60QL
Erk_TZ02HPL58
Erk_TZ02IOZUY
Erk_TZ02F37TH
Erk_TZ02G5WKH
Erk_TZ02GLXN5
Erk_TZ02F9ZFQ
Erk_TZ02JBH3U
Erk_TZ02H6IE1
Erk_TZ02JMNMJ
Erk_TZ02JG8DC
Eko_XU02JISZO
Vat_XU02JG555
Vat_XU02GEIW7
Eko_XU02GWIG8
Erk_TZ02HWU74
Vat_XU02H90MI
Erk_TZ02FRE70
Erk_TZ02GSVYB
Erk_TZ02G1PCR
Eko_XU02GF32U
Erk_TZ02IBTCI
Erk_TZ02HR2W4
MSp_MO01E0EOR
Vat_XU02F1ORX
Vat_XU02IOHIF
MSp_5H02JL8IX
Erk_TZ02HST9X
Erk_TZ02IMP4P
Erk_TZ02IN3IO
Erk_TZ02GFA5U
Erk_TZ02INDM6
MSp_5H02H2AOU
Eko_XU02GGSST
Erk_TZ02HPE0V
Eko_XU02GEHAS
Erk_TZ02GNIL1
Erk_TZ02HJJLB
Vat_XU02I4E2T
MSu_QV02JD1LO
MSu_QV02HCR30
Erk_TZ02GCBD7
Erk_TZ02F8CV9
Erk_TZ02GQH57
Erk_TZ02GZEGD
Eko_XU02H48S2
DSu_QV02JO6P7
Erk_TZ02JEV6X
Erk_TZ02F8KK4
B_L15_2246772218
B_J10_2246770828
MSp_A101CIVDE
Erk_TZ02FHQ0L
Erk_TZ02GIR4J
B_C06_2264663016
MSu_QV01EBQC3
Erk_TZ02H0NMQ
MSu_QV02FWZ3V
Erk_TZ02F7ISB
DSu_QV02GT6VF
DSu_QV01CECR4
DSu_QV01ER9XQ
DSu_QV02HOHC6
DSu_QV02H5SX0
C_M09_2236659758
DSu_QV02FN7J4
DSu_QV02IMGRN
Erk_TZ02JODLL
Eko_XU02FV26F
Erk_TZ02IKC8T
C07_2236669570
MSp_A101DV19M
DSp_QV02IYBON
DSu_QV01A8WPX
B11_2246773044
DSu_QV01D84OT
96
90
64
80
71
50
91
97
96
83
78
87
66
93
93
93
82
60
91
61
56
68
60
98
55
55
92
54
52
75
100
0.05

74 nusA

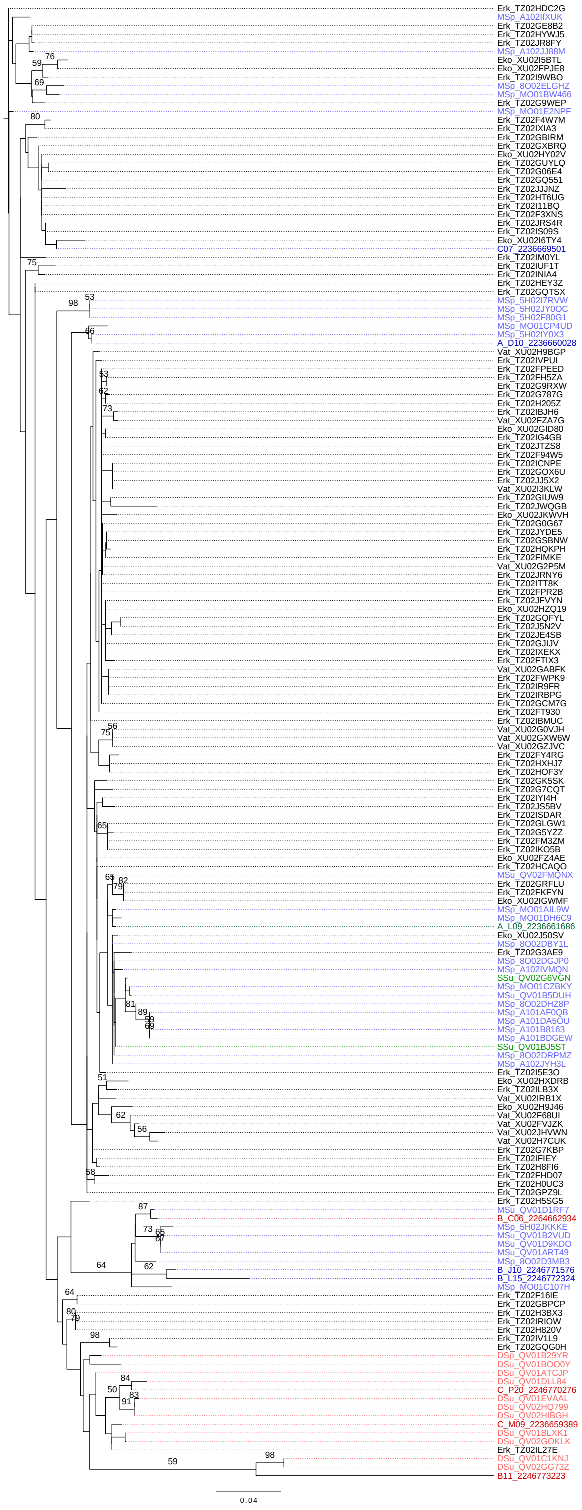

Erk_TZ02HDC2G
MSp_A102IIXUK
Erk_TZ02GE8B2
Erk_TZ02HYWJ5
Erk_TZ02JR8FY
MSp_A102JJ88M
Eko_XU02I5BTL
Eko_XU02FPJE8
Erk_TZ02I9WBO
MSp_8O02ELGHZ
MSp_MO01BW466
Erk_TZ02G9WEP
MSp_MO01E2NPF
Erk_TZ02F4W7M
Erk_TZ02IXIA3
Erk_TZ02GBIRM
Erk_TZ02GXBRQ
Eko_XU02HY02V
Erk_TZ02GUYLQ
Erk_TZ02G06E4
Erk_TZ02GQ551
Erk_TZ02JJJNZ
Erk_TZ02HT6UG
Erk_TZ02I11BQ
Erk_TZ02F3XNS
Erk_TZ02JRS4R
Erk_TZ02IS09S
Eko_XU02I6TY4
C07_2236669501
Erk_TZ02IM0YL
Erk_TZ02IUF1T
Erk_TZ02INIA4
Erk_TZ02HEY3Z
Erk_TZ02GQTSX
MSp_5H02I7RVW
MSp_5H02JY0OC
MSp_5H02F80G1
MSp_MO01CP4UD
MSp_5H02IY0X3
A_D10_2236660028
Vat_XU02H9BGP
Erk_TZ02IVPUI
Erk_TZ02FPEED
Erk_TZ02FH5ZA
Erk_TZ02G9RXW
Erk_TZ02G787G
Erk_TZ02H205Z
Erk_TZ02IBJH6
Vat_XU02FZA7G
Eko_XU02GID80
Erk_TZ02IG4GB
Erk_TZ02JTZS8
Erk_TZ02F94W5
Erk_TZ02ICNPE
Erk_TZ02GOX6U
Erk_TZ02JJ5X2
Vat_XU02I3KLW
Erk_TZ02GIUW9
Erk_TZ02JWQGB
Eko_XU02JKWVH
Erk_TZ02G0G67
Erk_TZ02JYDE5
Erk_TZ02GSBNW
Erk_TZ02HQKPH
Erk_TZ02FIMKE
Vat_XU02G2P5M
Erk_TZ02JRNY6
Erk_TZ02ITT8K
Erk_TZ02FPR2B
Erk_TZ02JFVYN
Eko_XU02HZQ19
Erk_TZ02GQFYL
Erk_TZ02J5N2V
Erk_TZ02JE4SB
Erk_TZ02GJIJV
Erk_TZ02IXEKX
Erk_TZ02FTIX3
Vat_XU02GABFK
Erk_TZ02FWPK9
Erk_TZ02IR9FR
Erk_TZ02IRBPG
Erk_TZ02GCM7G
Erk_TZ02FT930
Erk_TZ02IBMUC
Vat_XU02G0VJH
Vat_XU02GXW6W
Vat_XU02GZJVC
Erk_TZ02FY4RG
Erk_TZ02HXHJ7
Erk_TZ02HOF3Y
Erk_TZ02GK5SK
Erk_TZ02G7CQT
Erk_TZ02IYI4H
Erk_TZ02JS5BV
Erk_TZ02ISDAR
Erk_TZ02GLGW1
Erk_TZ02G5YZZ
Erk_TZ02FM3ZM
Erk_TZ02IKO5B
Eko_XU02FZ4AE
Erk_TZ02HCAQO
MSu_QV02FMQNX
Erk_TZ02GRFLU
Erk_TZ02FKFYN
Eko_XU02IGWMF
MSp_MO01AIL9W
MSp_MO01DH6C9
A_L09_2236661686
Eko_XU02J50SV
MSp_8O02DBY1L
Erk_TZ02G3AE9
MSp_8O02DGJP0
MSp_A102IVMQN
SSu_QV02G6VGN
MSp_MO01CZBKY
MSu_QV01B5DUH
MSp_8O02DHZ8P
MSp_A101AF0QB
MSp_A101DA5OU
MSp_A101B8163
MSp_A101BDGEW
SSu_QV01BJ5ST
MSp_8O02DRPMZ
MSp_A102JYH3L
Erk_TZ02I5E3O
Eko_XU02HXDRB
Erk_TZ02ILB3X
Vat_XU02IRB1X
Eko_XU02H9J46
Vat_XU02F68UI
Vat_XU02FVJZK
Vat_XU02JHVWN
Vat_XU02H7CUK
Erk_TZ02G7KBP
Erk_TZ02IFIEY
Erk_TZ02H8FI6
Erk_TZ02FHD07
Erk_TZ02H0UC3
Erk_TZ02GPZ9L
Erk_TZ02H5SG5
MSu_QV01D1RF7
B_C06_2264662934
MSp_5H02JKKKE
MSu_QV01B2VUD
MSu_QV01D9KDO
MSu_QV01ART49
MSp_8O02D3MB3
B_J10_2246771576
B_L15_2246772324
MSp_MO01C107H
Erk_TZ02F16IE
Erk_TZ02GBPCP
Erk_TZ02H3BX3
Erk_TZ02IRIOW
Erk_TZ02H820V
Erk_TZ02IV1L9
Erk_TZ02GQG0H
DSp_QV01B29YR
DSu_QV01BOO0Y
DSu_QV01ATCJP
DSu_QV01DLL84
C_P20_2246770276
DSu_QV01EVAAL
DSu_QV02HQ799
DSu_QV02HIBGH
C_M09_2236659389
DSu_QV01BLXK1
DSu_QV02GOKLK
Erk_TZ02IL27E
DSu_QV01C1KNJ
DSu_QV02GG73Z
B11_2246773223
0.04

13 ftsK

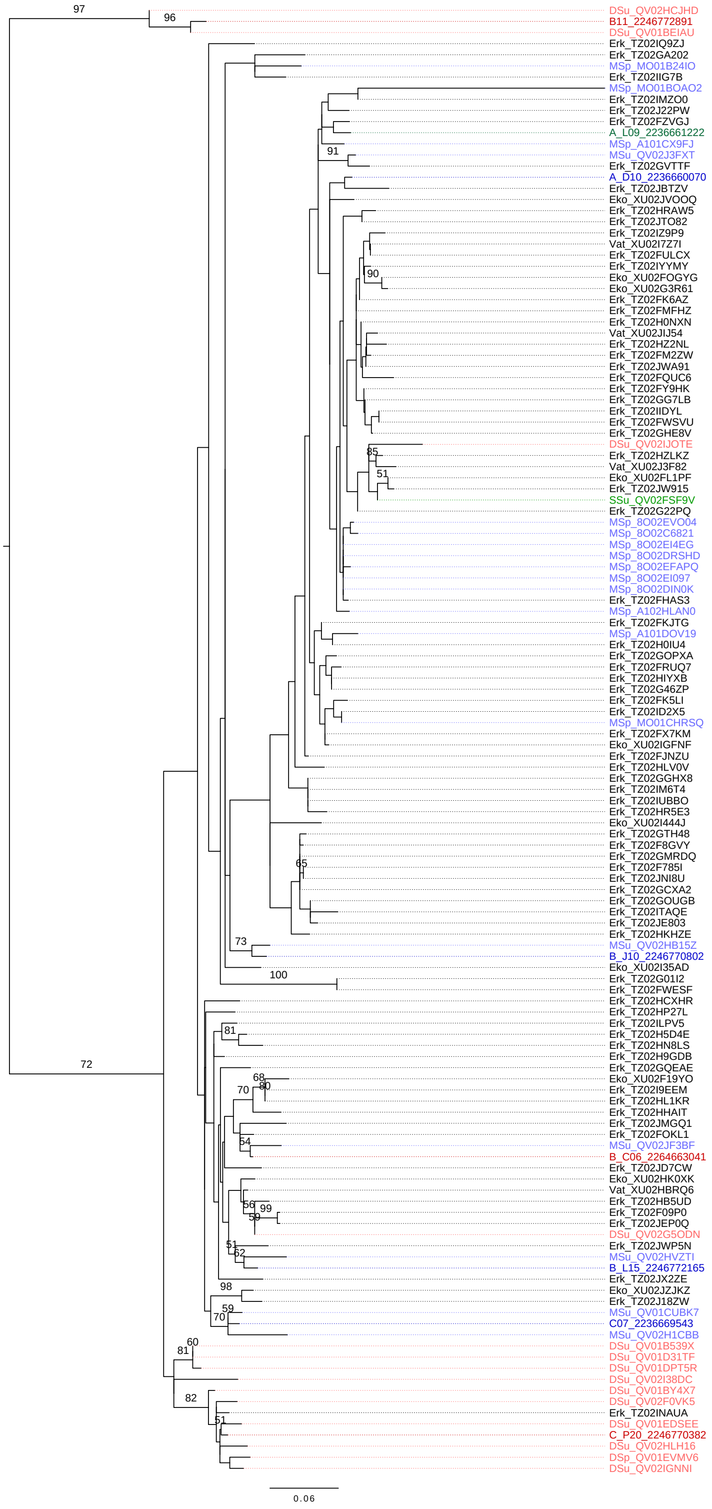

DSu_QV02HCJHD
B11_2246772891
DSu_QV01BEIAU
Erk_TZ02IQ9ZJ
Erk_TZ02GA202
MSp_MO01B24IO
Erk_TZ02IIG7B
MSp_MO01BOAO2
Erk_TZ02IMZO0
Erk_TZ02J22PW
Erk_TZ02FZVGJ
A_L09_2236661222
MSp_A101CX9FJ
MSu_QV02J3FXT
Erk_TZ02GVTTF
A_D10_2236660070
Erk_TZ02JBTZV
Eko_XU02JVOOQ
Erk_TZ02HRAW5
Erk_TZ02JTO82
Erk_TZ02IZ9P9
Vat_XU02I7Z7I
Erk_TZ02FULCX
Erk_TZ02IYYMY
Eko_XU02FOGYG
Eko_XU02G3R61
Erk_TZ02FK6AZ
Erk_TZ02FMFHZ
Erk_TZ02H0NXN
Vat_XU02JIJ54
Erk_TZ02HZ2NL
Erk_TZ02FM2ZW
Erk_TZ02JWA91
Erk_TZ02FQUC6
Erk_TZ02FY9HK
Erk_TZ02GG7LB
Erk_TZ02IIDYL
Erk_TZ02FWSVU
Erk_TZ02GHE8V
DSu_QV02IJOTE
Erk_TZ02HZLKZ
Vat_XU02J3F82
Eko_XU02FL1PF
Erk_TZ02JW915
SSu_QV02FSF9V
Erk_TZ02G22PQ
MSp_8O02EVO04
MSp_8O02C6821
MSp_8O02EI4EG
MSp_8O02DRSHD
MSp_8O02EFAPQ
MSp_8O02EI097
MSp_8O02DIN0K
Erk_TZ02FHAS3
MSp_A102HLAN0
Erk_TZ02FKJTG
MSp_A101DOV19
Erk_TZ02H0IU4
Erk_TZ02GOPXA
Erk_TZ02FRUQ7
Erk_TZ02HIYXB
Erk_TZ02G46ZP
Erk_TZ02FK5LI
Erk_TZ02ID2X5
MSp_MO01CHRSQ
Erk_TZ02FX7KM
Eko_XU02IGFNF
Erk_TZ02FJNZU
Erk_TZ02HLV0V
Erk_TZ02GGHX8
Erk_TZ02IM6T4
Erk_TZ02IUBBO
Erk_TZ02HR5E3
Eko_XU02I444J
Erk_TZ02GTH48
Erk_TZ02F8GVY
Erk_TZ02GMRDQ
Erk_TZ02F785I
Erk_TZ02JNI8U
Erk_TZ02GCXA2
Erk_TZ02GOUGB
Erk_TZ02ITAQE
Erk_TZ02JE803
Erk_TZ02HKHZE
MSu_QV02HB15Z
B_J10_2246770802
Eko_XU02I35AD
Erk_TZ02G01I2
Erk_TZ02FWESF
Erk_TZ02HCXHR
Erk_TZ02HP27L
Erk_TZ02ILPV5
Erk_TZ02H5D4E
Erk_TZ02HN8LS
Erk_TZ02H9GDB
Erk_TZ02GQEAE
Eko_XU02F19YO
Erk_TZ02I9EEM
Erk_TZ02HL1KR
Erk_TZ02HHAIT
Erk_TZ02JMGQ1
Erk_TZ02FOKL1
MSu_QV02JF3BF
B_C06_2264663041
Erk_TZ02JD7CW
Eko_XU02HK0XK
Vat_XU02HBRQ6
Erk_TZ02HB5UD
Erk_TZ02F09P0
Erk_TZ02JEP0Q
DSu_QV02G5ODN
Erk_TZ02JWP5N
MSu_QV02HVZTI
B_L15_2246772165
Erk_TZ02JX2ZE
Eko_XU02JZJKZ
Erk_TZ02J18ZW
MSu_QV01CUBK7
C07_2236669543
MSu_QV02H1CBB
DSu_QV01B539X
DSu_QV01D31TF
DSu_QV01DPT5R
DSu_QV02I38DC
DSu_QV01BY4X7
DSu_QV02F0VK5
Erk_TZ02INAUA
DSu_QV01EDSEE
C_P20_2246770382
DSu_QV02HLH16
DSp_QV01EVMV6
DSu_QV02IGNNI
0.06

33 rho

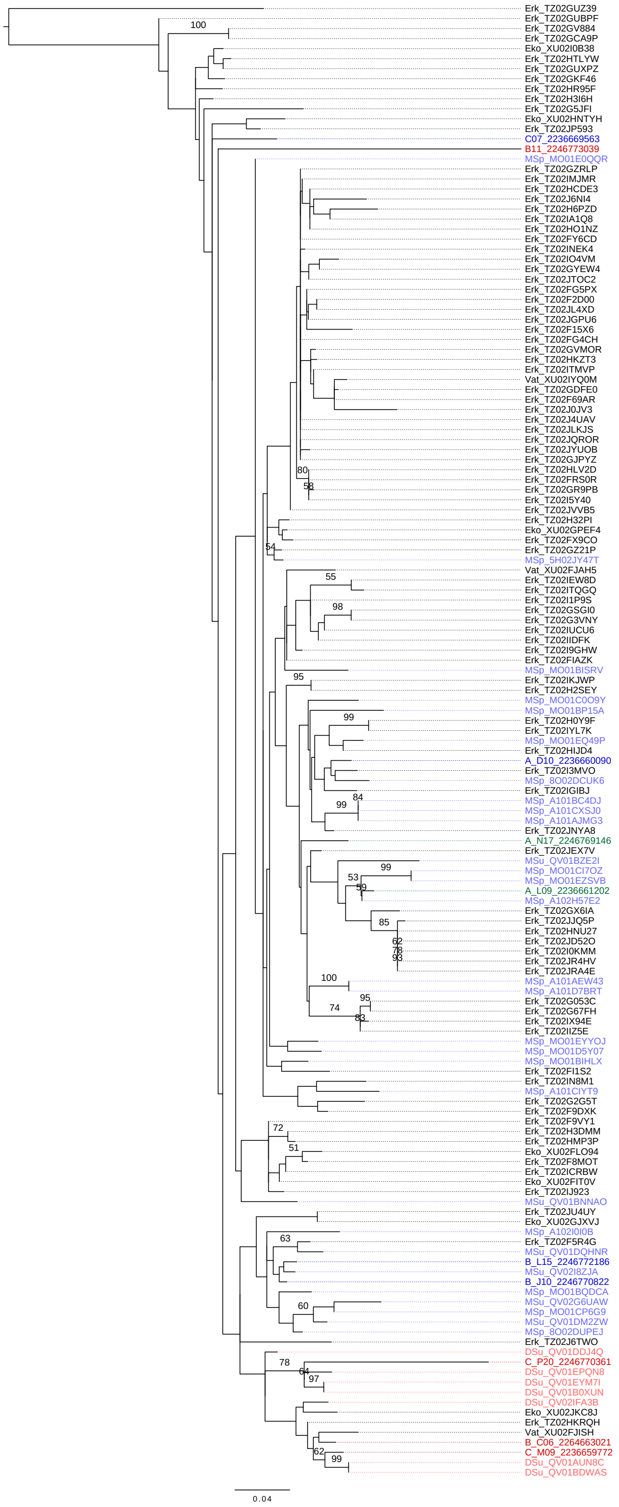

Erk_TZ02GUZ39
Erk_TZ02GUBPF
Erk_TZ02GV884
Erk_TZ02GCA9P
Eko_XU02I0B38
Erk_TZ02HTLYW
Erk_TZ02GUXPZ
Erk_TZ02GKF46
Erk_TZ02HR95F
Erk_TZ02H3I6H
Erk_TZ02G5JFI
Eko_XU02HNTYH
Erk_TZ02JP593
C07_2236669563
B11_2246773039
MSp_MO01E0QQR
Erk_TZ02GZRLP
Erk_TZ02IMJMR
Erk_TZ02HCDE3
Erk_TZ02J6NI4
Erk_TZ02H6PZD
Erk_TZ02IA1Q8
Erk_TZ02HO1NZ
Erk_TZ02FY6CD
Erk_TZ02INEK4
Erk_TZ02IO4VM
Erk_TZ02GYEW4
Erk_TZ02JTOC2
Erk_TZ02FG5PX
Erk_TZ02F2D00
Erk_TZ02JL4XD
Erk_TZ02JGPU6
Erk_TZ02F15X6
Erk_TZ02FG4CH
Erk_TZ02GVMOR
Erk_TZ02HKZT3
Erk_TZ02ITMVP
Vat_XU02IYQ0M
Erk_TZ02GDFE0
Erk_TZ02F69AR
Erk_TZ02J0JV3
Erk_TZ02J4UAV
Erk_TZ02JLKJS
Erk_TZ02JQROR
Erk_TZ02JYUOB
Erk_TZ02GJPYZ
Erk_TZ02HLV2D
Erk_TZ02FRS0R
Erk_TZ02GR9PB
Erk_TZ02I5Y40
Erk_TZ02JVVB5
Erk_TZ02H32PI
Eko_XU02GPEF4
Erk_TZ02FX9CO
Erk_TZ02GZ21P
MSp_5H02JY47T
Vat_XU02FJAH5
Erk_TZ02IEW8D
Erk_TZ02ITQGQ
Erk_TZ02I1P9S
Erk_TZ02GSGI0
Erk_TZ02G3VNY
Erk_TZ02IUCU6
Erk_TZ02IIDFK
Erk_TZ02I9GHW
Erk_TZ02FIAZK
MSp_MO01BISRV
Erk_TZ02IKJWP
Erk_TZ02H2SEY
MSp_MO01C0O9Y
MSp_MO01BP15A
Erk_TZ02H0Y9F
Erk_TZ02IYL7K
MSp_MO01EQ49P
Erk_TZ02HIJD4
A_D10_2236660090
Erk_TZ02I3MVO
MSp_8O02DCUK6
Erk_TZ02IGIBJ
MSp_A101BC4DJ
MSp_A101CXSJ0
MSp_A101AJMG3
Erk_TZ02JNYA8
A_N17_2246769146
Erk_TZ02JEX7V
MSu_QV01BZE2I
MSp_MO01CI7OZ
MSp_MO01EZSVB
A_L09_2236661202
MSp_A102H57E2
Erk_TZ02GX6IA
Erk_TZ02JJQ5P
Erk_TZ02HNU27
Erk_TZ02JD52O
Erk_TZ02I0KMM
Erk_TZ02JR4HV
Erk_TZ02JRA4E
MSp_A101AEW43
MSp_A101D7BRT
Erk_TZ02G053C
Erk_TZ02G67FH
Erk_TZ02IX94E
Erk_TZ02IIZ5E
MSp_MO01EYYOJ
MSp_MO01D5Y07
MSp_MO01BIHLX
Erk_TZ02FI1S2
Erk_TZ02IN8M1
MSp_A101CIYT9
Erk_TZ02G2G5T
Erk_TZ02F9DXK
Erk_TZ02F9VY1
Erk_TZ02H3DMM
Erk_TZ02HMP3P
Eko_XU02FLO94
Erk_TZ02F8MOT
Erk_TZ02ICRBW
Eko_XU02FIT0V
Erk_TZ02IJ923
MSu_QV01BNNAO
Erk_TZ02JU4UY
Eko_XU02GJXVJ
MSp_A102I0I0B
Erk_TZ02F5R4G
MSu_QV01DQHNR
B_L15_2246772186
MSu_QV02I8ZJA
B_J10_2246770822
MSp_MO01BQDCA
MSu_QV02G6UAW
MSp_MO01CP6G9
MSu_QV01DM2ZW
MSp_8O02DUPEJ
Erk_TZ02J6TWO
DSu_QV01DDJ4Q
C_P20_2246770361
DSu_QV01EPQN8
DSu_QV01EYM7I
DSu_QV01B0XUN
DSu_QV02IFA3B
Eko_XU02JKC8J
Erk_TZ02HKRQH
Vat_XU02FJISH
B_C06_2264663021
C_M09_2236659772
DSu_QV01AUN8C
DSu_QV01BDWAS
0.04

45 tolC

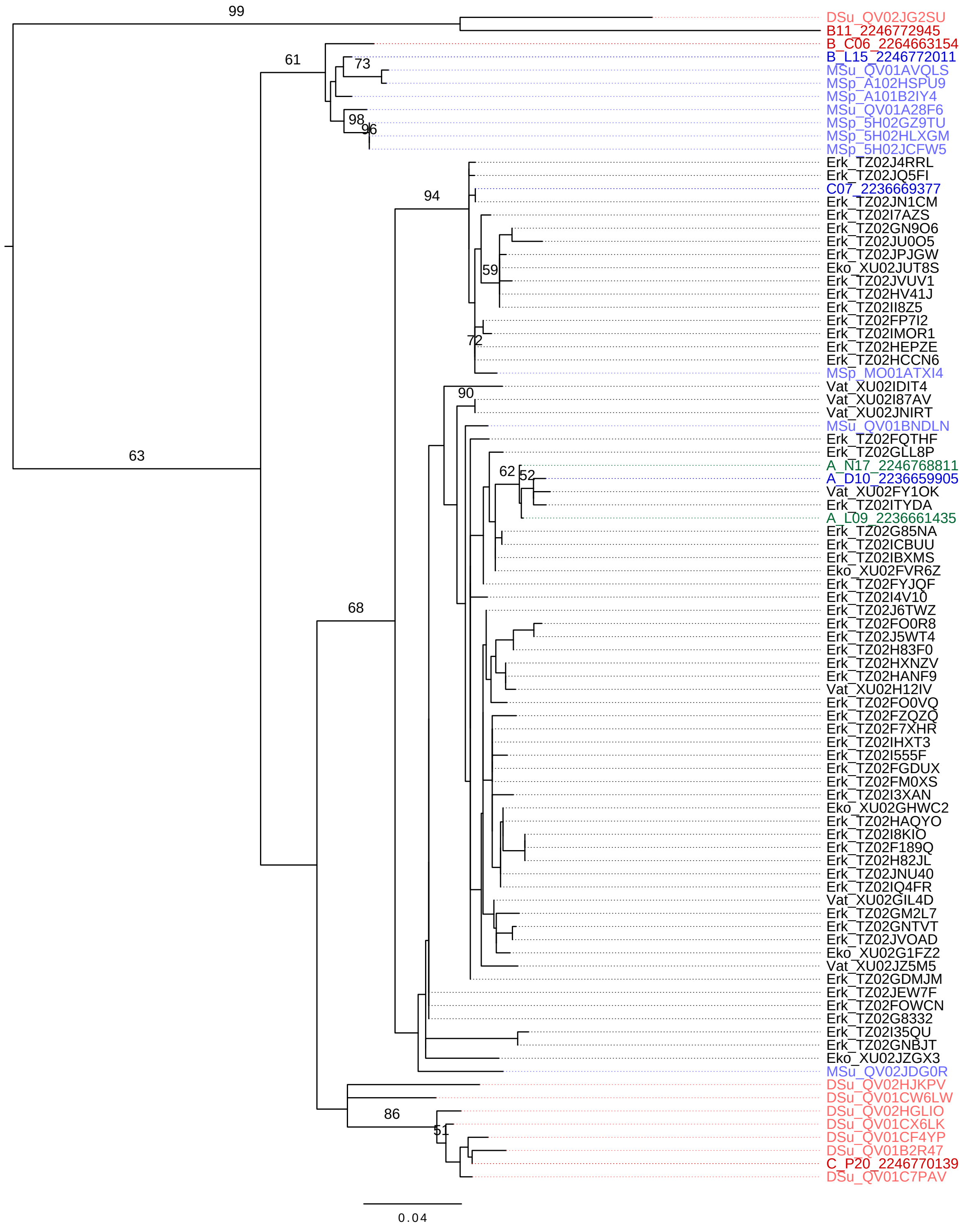

99
61
73
98
96
94
59
72
90
62
52
68
63
86
51
DSu_QV02JG2SU
B11_2246772945
B_C06_2264663154
B_L15_2246772011
MSu_QV01AVQLS
MSp_A102HSPU9
MSp_A101B2IY4
MSu_QV01A28F6
MSp_5H02GZ9TU
MSp_5H02HLXGM
MSp_5H02JCFW5
Erk_TZ02J4RRL
Erk_TZ02JQ5FI
C07_2236669377
Erk_TZ02JN1CM
Erk_TZ02I7AZS
Erk_TZ02GN9O6
Erk_TZ02JU0O5
Erk_TZ02JPJGW
Eko_XU02JUT8S
Erk_TZ02JVUV1
Erk_TZ02HV41J
Erk_TZ02II8Z5
Erk_TZ02FP7I2
Erk_TZ02IMOR1
Erk_TZ02HEPZE
Erk_TZ02HCCN6
MSp_MO01ATXI4
Vat_XU02IDIT4
Vat_XU02I87AV
Vat_XU02JNIRT
MSu_QV01BNDLN
Erk_TZ02FQTHF
Erk_TZ02GLL8P
A_N17_2246768811
A_D10_2236659905
Vat_XU02FY1OK
Erk_TZ02ITYDA
A_L09_2236661435
Erk_TZ02G85NA
Erk_TZ02ICBUU
Erk_TZ02IBXMS
Eko_XU02FVR6Z
Erk_TZ02FYJQF
Erk_TZ02I4V10
Erk_TZ02J6TWZ
Erk_TZ02FO0R8
Erk_TZ02J5WT4
Erk_TZ02H83F0
Erk_TZ02HXNZV
Erk_TZ02HANF9
Vat_XU02H12IV
Erk_TZ02FO0VQ
Erk_TZ02FZQZQ
Erk_TZ02F7XHR
Erk_TZ02IHXT3
Erk_TZ02I555F
Erk_TZ02FGDUX
Erk_TZ02FM0XS
Erk_TZ02I3XAN
Eko_XU02GHWC2
Erk_TZ02HAQYO
Erk_TZ02I8KIO
Erk_TZ02F189Q
Erk_TZ02H82JL
Erk_TZ02JNU40
Erk_TZ02IQ4FR
Vat_XU02GIL4D
Erk_TZ02GM2L7
Erk_TZ02GNTVT
Erk_TZ02JVOAD
Eko_XU02G1FZ2
Vat_XU02JZ5M5
Erk_TZ02GDMJM
Erk_TZ02JEW7F
Erk_TZ02FOWCN
Erk_TZ02G8332
Erk_TZ02I35QU
Erk_TZ02GNBJT
Eko_XU02JZGX3
MSu_QV02JDG0R
DSu_QV02HJKPV
DSu_QV01CW6LW
DSu_QV02HGLIO
DSu_QV01CX6LK
DSu_QV01CF4YP
DSu_QV01B2R47
C_P20_2246770139
DSu_QV01C7PAV
0.04

71 pnp

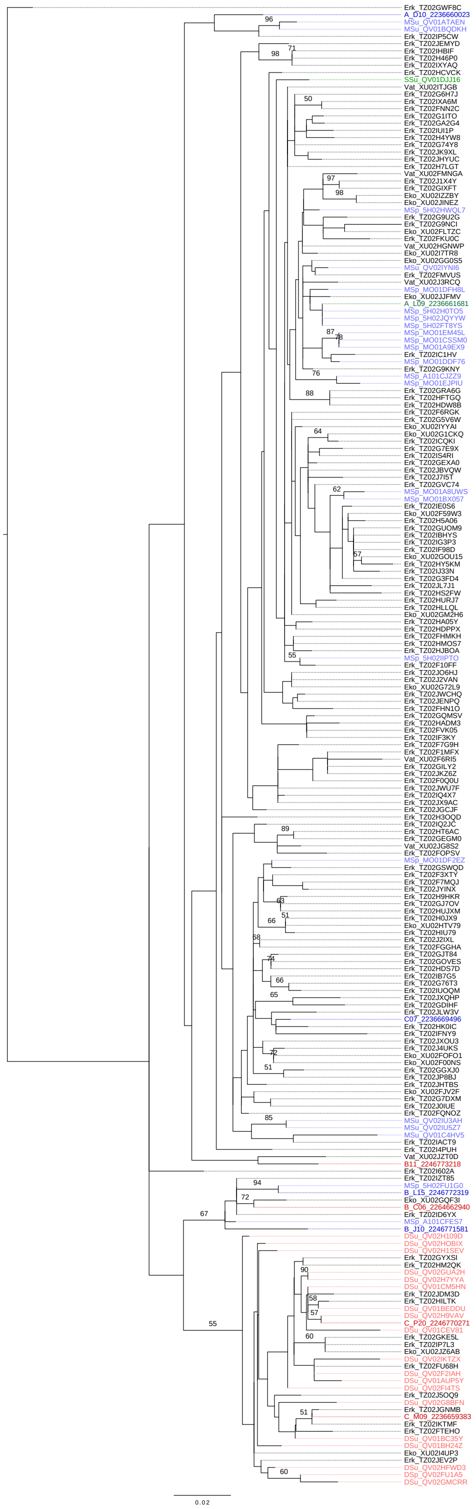

73 infB

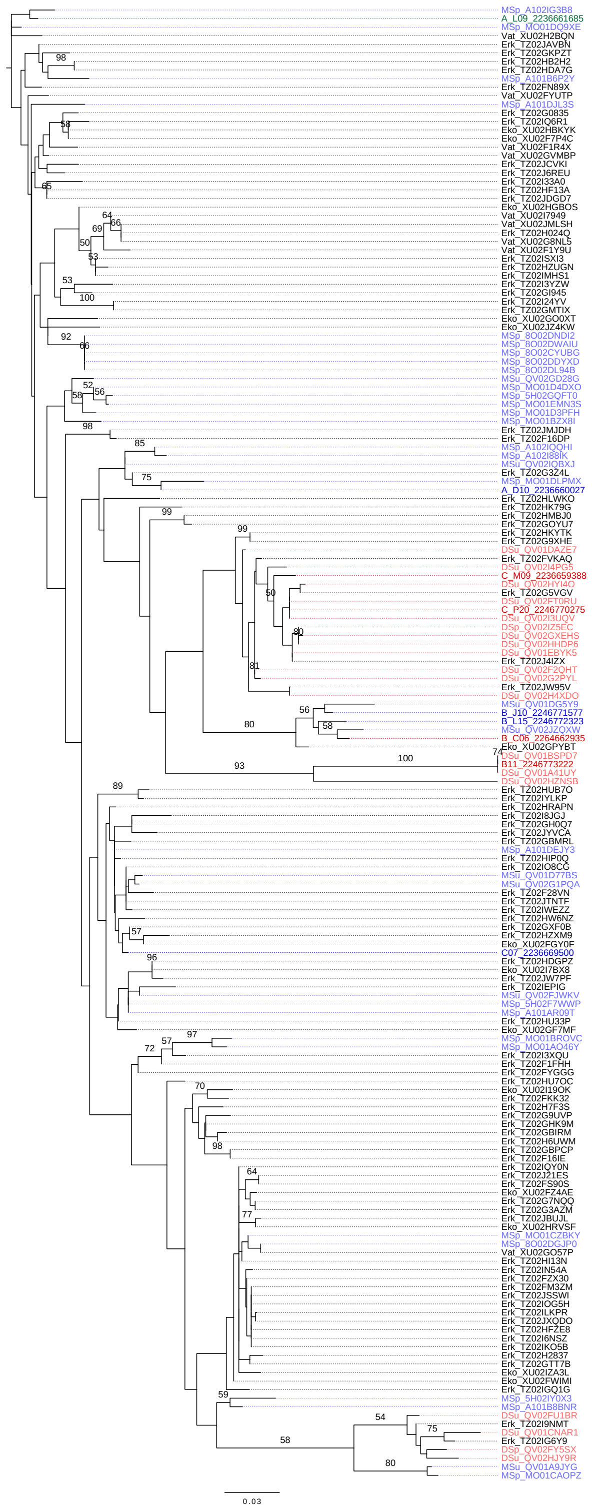

MSp_A102IG3B8
A_L09_2236661685
MSp_MO01DQ9XE
Vat_XU02H2BQN
Erk_TZ02JAVBN
Erk_TZ02GKPZT
Erk_TZ02HB2H2
Erk_TZ02HDA7G
MSp_A101B6P2Y
Erk_TZ02FN89X
Vat_XU02FYUTP
MSp_A101DJL3S
Erk_TZ02G0835
Erk_TZ02IQ6R1
Eko_XU02HBKYK
Eko_XU02F7P4C
Vat_XU02F1R4X
Vat_XU02GVMBP
Erk_TZ02JCVKI
Erk_TZ02J6REU
Erk_TZ02I33A0
Erk_TZ02HF13A
Erk_TZ02JDGD7
Eko_XU02HGBOS
Vat_XU02I7949
Vat_XU02JMLSH
Erk_TZ02H024Q
Vat_XU02G8NL5
Vat_XU02F1Y9U
Erk_TZ02ISXI3
Erk_TZ02HZUGN
Erk_TZ02IMHS1
Erk_TZ02I3YZW
Erk_TZ02GI945
Erk_TZ02I24YV
Erk_TZ02GMTIX
Eko_XU02GO0XT
Eko_XU02JZ4KW
MSp_8O02DNDI2
MSp_8O02DWAIU
MSp_8O02CYUBG
MSp_8O02DDYXD
MSp_8O02DL94B
MSu_QV02GD28G
MSp_MO01D4DXO
MSp_5H02GQFT0
MSp_MO01EMN3S
MSp_MO01D3PFH
MSp_MO01BZX8I
Erk_TZ02JMJDH
Erk_TZ02F16DP
MSp_A102IQQHI
MSp_A102I88IK
MSu_QV02IQBXJ
Erk_TZ02G3Z4L
MSp_MO01DLPMX
A_D10_2236660027
Erk_TZ02HLWKO
Erk_TZ02HK79G
Erk_TZ02HMBJ0
Erk_TZ02GOYU7
Erk_TZ02HKYTK
Erk_TZ02G9XHE
DSu_QV01DAZE7
Erk_TZ02FVKAQ
DSu_QV02I4PG5
C_M09_2236659388
DSu_QV02HYI4O
Erk_TZ02G5VGV
DSu_QV02FT0RU
C_P20_2246770275
DSu_QV02I3UQV
DSp_QV02IZ5EC
DSu_QV02GXEHS
DSu_QV02HHDP6
DSu_QV01EBYK5
Erk_TZ02J4IZX
DSu_QV02F2QHT
DSu_QV02G2PYL
Erk_TZ02JW95V
DSu_QV02H4XDO
MSu_QV01DG5Y9
B_J10_2246771577
B_L15_2246772323
MSu_QV02JZQXW
B_C06_2264662935
Eko_XU02GPYBT
DSu_QV01BSPD7
B11_2246773222
DSu_QV01A41UY
DSu_QV02HZNSB
Erk_TZ02HUB7O
Erk_TZ02IYLKP
Erk_TZ02HRAPN
Erk_TZ02I8JGJ
Erk_TZ02GH0Q7
Erk_TZ02JYVCA
Erk_TZ02GBMRL
MSp_A101DEJY3
Erk_TZ02HIP0Q
Erk_TZ02IO8CG
MSu_QV01D77BS
MSu_QV02G1PQA
Erk_TZ02F28VN
Erk_TZ02JTNTF
Erk_TZ02IWEZZ
Erk_TZ02HW6NZ
Erk_TZ02GXF0B
Erk_TZ02HZXM9
Eko_XU02FGY0F
C07_2236669500
Erk_TZ02HDGPZ
Eko_XU02I7BX8
Erk_TZ02JW7PF
Erk_TZ02IEPIG
MSu_QV02FJWKV
MSp_5H02F7WWP
MSp_A101AR09T
Erk_TZ02HU33P
Eko_XU02GF7MF
MSp_MO01BROVC
MSp_MO01AO46Y
Erk_TZ02I3XQU
Erk_TZ02F1FHH
Erk_TZ02FYGGG
Erk_TZ02HU7OC
Eko_XU02I19OK
Erk_TZ02FKK32
Erk_TZ02H7F3S
Erk_TZ02G9UVP
Erk_TZ02GHK9M
Erk_TZ02GBIRM
Erk_TZ02H6UWM
Erk_TZ02GBPCP
Erk_TZ02F16IE
Erk_TZ02IQY0N
Erk_TZ02J21ES
Erk_TZ02FS90S
Eko_XU02FZ4AE
Erk_TZ02G7NQQ
Erk_TZ02G3AZM
Erk_TZ02JBUJL
Eko_XU02HRVSF
MSp_MO01CZBKY
MSp_8O02DGJP0
Vat_XU02GO57P
Erk_TZ02HI13N
Erk_TZ02IN54A
Erk_TZ02FZX30
Erk_TZ02FM3ZM
Erk_TZ02JSSWI
Erk_TZ02IOG5H
Erk_TZ02ILKPR
Erk_TZ02JXQDO
Erk_TZ02HFZE8
Erk_TZ02I6NSZ
Erk_TZ02IKO5B
Erk_TZ02H2837
Erk_TZ02GTT7B
Eko_XU02IZA3L
Eko_XU02FWIMI
Erk_TZ02IGQ1G
MSp_5H02IY0X3
MSp_A101B8BNR
DSu_QV02FU1BR
Erk_TZ02I9NMT
DSu_QV01CNAR1
Erk_TZ02IG6Y9
DSp_QV02FY5SX
DSu_QV02HJY9R
MSu_QV01A9JYG
MSp_MO01CAOPZ
0.03

81 mgtE

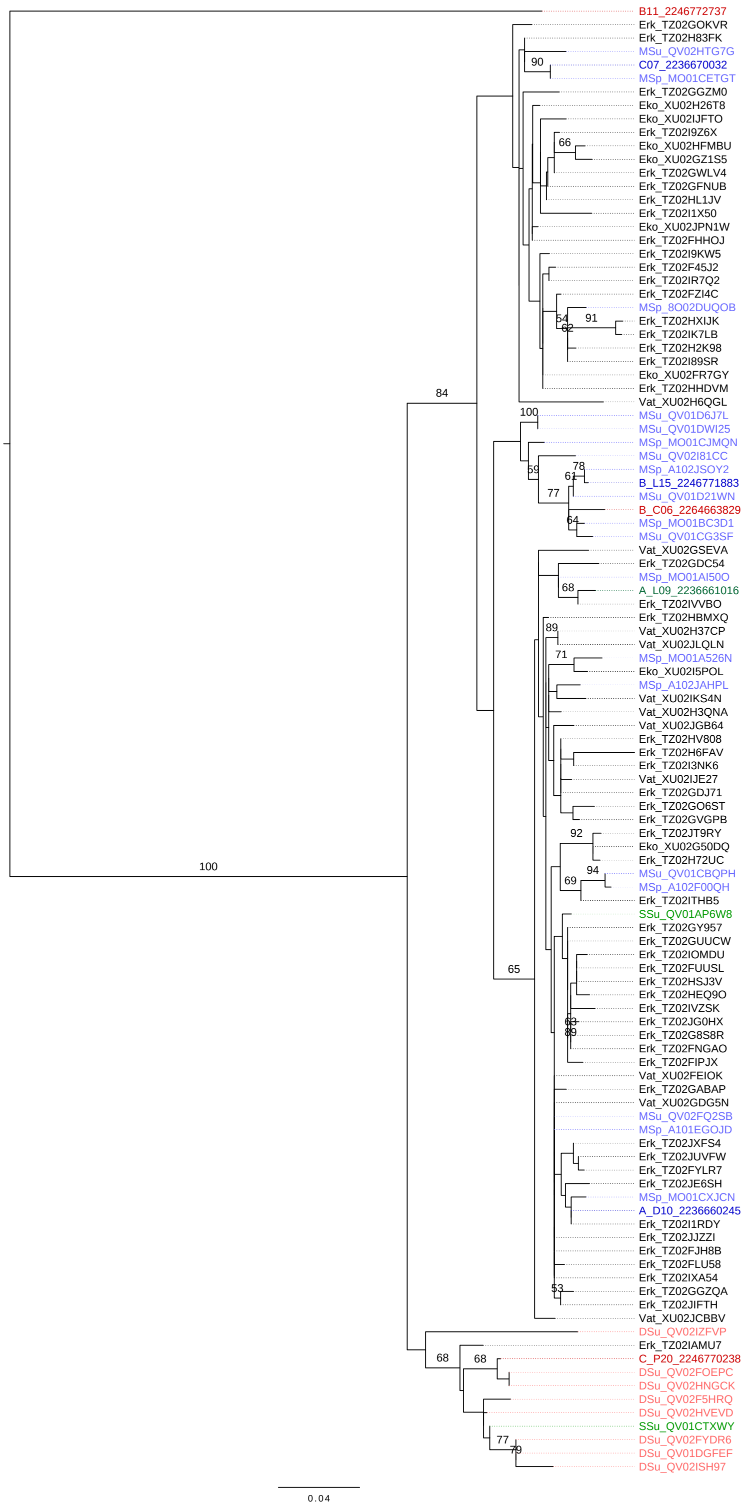
B11_2246772737
Erk_TZ02GOKVR
Erk_TZ02H83FK
MSu_QV02HTG7G
C07_2236670032
MSp_MO01CETGT
Erk_TZ02GGZM0
Eko_XU02H26T8
Eko_XU02IJFTO
Erk_TZ02I9Z6X
Eko_XU02HFMBU
Eko_XU02GZ1S5
Erk_TZ02GWLV4
Erk_TZ02GFNUB
Erk_TZ02HL1JV
Erk_TZ02I1X50
Eko_XU02JPN1W
Erk_TZ02FHHOJ
Erk_TZ02I9KW5
Erk_TZ02F45J2
Erk_TZ02IR7Q2
Erk_TZ02FZI4C
MSp_8O02DUQOB
Erk_TZ02HXIJK
Erk_TZ02IK7LB
Erk_TZ02H2K98
Erk_TZ02I89SR
Eko_XU02FR7GY
Erk_TZ02HHDVM
Vat_XU02H6QGL
MSu_QV01D6J7L
MSu_QV01DWI25
MSp_MO01CJMQN
MSu_QV02I81CC
MSp_A102JSOY2
B_L15_2246771883
MSu_QV01D21WN
B_C06_2264663829
MSp_MO01BC3D1
MSu_QV01CG3SF
Vat_XU02GSEVA
Erk_TZ02GDC54
MSp_MO01AI50O
A_L09_2236661016
Erk_TZ02IVVBO
Erk_TZ02HBMXQ
Vat_XU02H37CP
Vat_XU02JLQLN
MSp_MO01A526N
Eko_XU02I5POL
MSp_A102JAHPL
Vat_XU02IKS4N
Vat_XU02H3QNA
Vat_XU02JGB64
Erk_TZ02HV808
Erk_TZ02H6FAV
Erk_TZ02I3NK6
Vat_XU02IJE27
Erk_TZ02GDJ71
Erk_TZ02GO6ST
Erk_TZ02GVGPB
Erk_TZ02JT9RY
Eko_XU02G50DQ
Erk_TZ02H72UC
MSu_QV01CBQPH
MSp_A102F00QH
Erk_TZ02ITHB5
SSu_QV01AP6W8
Erk_TZ02GY957
Erk_TZ02GUUCW
Erk_TZ02IOMDU
Erk_TZ02FUUSL
Erk_TZ02HSJ3V
Erk_TZ02HEQ9O
Erk_TZ02IVZSK
Erk_TZ02JG0HX
Erk_TZ02G8S8R
Erk_TZ02FNGAO
Erk_TZ02FIPJX
Vat_XU02FEIOK
Erk_TZ02GABAP
Vat_XU02GDG5N
MSu_QV02FQ2SB
MSp_A101EGOJD
Erk_TZ02JXFS4
Erk_TZ02JUVFW
Erk_TZ02FYLR7
Erk_TZ02JE6SH
MSp_MO01CXJCN
A_D10_2236660245
Erk_TZ02I1RDY
Erk_TZ02JJZZI
Erk_TZ02FJH8B
Erk_TZ02FLU58
Erk_TZ02IXA54
Erk_TZ02GGZQA
Erk_TZ02JIFTH
Vat_XU02JCBBV
DSu_QV02IZFVP
Erk_TZ02IAMU7
C_P20_2246770238
DSu_QV02FOEPC
DSu_QV02HNGCK
DSu_QV02F5HRQ
DSu_QV02HVEVD
SSu_QV01CTXWY
DSu_QV02FYDR6
DSu_QV01DGFEF
DSu_QV02ISH97
90
66
54
62
91
84
100
59
78
61
77
64
68
89
71
92
94
69
63
89
53
65
100
68
68
77
79
0.04

85 ligA

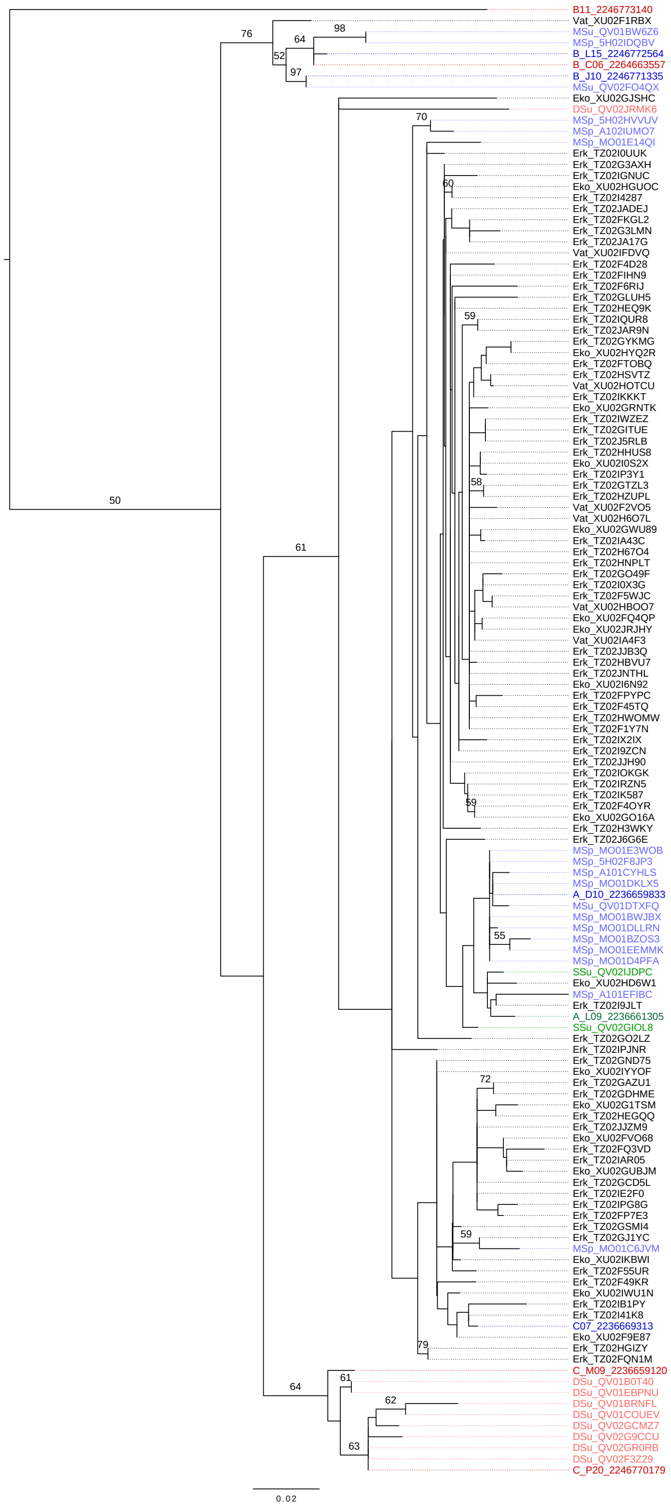
B11_2246773140
Vat_XU02F1RBX
MSu_QV01BW6Z6
MSp_5H02IDQBV
B_L15_2246772564
B_C06_2264663557
B_J10_2246771335
MSu_QV02FO4QX
Eko_XU02GJSHC
DSu_QV02JRMK6
MSp_5H02HVVUV
MSp_A102IUMO7
MSp_MO01E14QI
Erk_TZ02I0UUK
Erk_TZ02G3AXH
Erk_TZ02IGNUC
Eko_XU02HGUOC
Erk_TZ02I4287
Erk_TZ02JADEJ
Erk_TZ02FKGL2
Erk_TZ02G3LMN
Erk_TZ02JA17G
Vat_XU02IFDVQ
Erk_TZ02F4D28
Erk_TZ02FIHN9
Erk_TZ02F6RIJ
Erk_TZ02GLUH5
Erk_TZ02HEQ9K
Erk_TZ02IQUR8
Erk_TZ02JAR9N
Erk_TZ02GYKMG
Eko_XU02HYQ2R
Erk_TZ02FTOBQ
Erk_TZ02HSVTZ
Vat_XU02HOTCU
Erk_TZ02IKKKT
Eko_XU02GRNTK
Erk_TZ02IWZEZ
Erk_TZ02GITUE
Erk_TZ02J5RLB
Erk_TZ02HHUS8
Eko_XU02I0S2X
Erk_TZ02IP3Y1
Erk_TZ02GTZL3
Erk_TZ02HZUPL
Vat_XU02F2VO5
Vat_XU02H6O7L
Eko_XU02GWU89
Erk_TZ02IA43C
Erk_TZ02H67O4
Erk_TZ02HNPLT
Erk_TZ02GO49F
Erk_TZ02I0X3G
Erk_TZ02F5WJC
Vat_XU02HBOO7
Eko_XU02FQ4QP
Eko_XU02JRJHY
Vat_XU02IA4F3
Erk_TZ02JJB3Q
Erk_TZ02HBVU7
Erk_TZ02JNTHL
Eko_XU02I6N92
Erk_TZ02FPYPC
Erk_TZ02F45TQ
Erk_TZ02HWOMW
Erk_TZ02F1Y7N
Erk_TZ02IX2IX
Erk_TZ02I9ZCN
Erk_TZ02JJH90
Erk_TZ02IOKGK
Erk_TZ02IRZN5
Erk_TZ02IK587
Erk_TZ02F4OYR
Eko_XU02GO16A
Erk_TZ02H3WKY
Erk_TZ02J6G6E
MSp_MO01E3WOB
MSp_5H02F8JP3
MSp_A101CYHLS
MSp_MO01DKLX5
A_D10_2236659833
MSu_QV01DTXFQ
MSp_MO01BWJBX
MSp_MO01DLLRN
MSp_MO01BZOS3
MSp_MO01EEMMK
MSp_MO01D4PFA
SSu_QV02IJDPC
Eko_XU02HD6W1
MSp_A101EFIBC
Erk_TZ02I9JLT
A_L09_2236661305
SSu_QV02GIOL8
Erk_TZ02GO2LZ
Erk_TZ02IPJNR
Erk_TZ02GND75
Eko_XU02IYYOF
Erk_TZ02GAZU1
Erk_TZ02GDHME
Eko_XU02G1TSM
Erk_TZ02HEGQQ
Erk_TZ02JJZM9
Eko_XU02FVO68
Erk_TZ02FQ3VD
Erk_TZ02IAR05
Eko_XU02GUBJM
Erk_TZ02GCD5L
Erk_TZ02IE2F0
Erk_TZ02IPG8G
Erk_TZ02FP7E3
Erk_TZ02GSMI4
Erk_TZ02GJ1YC
MSp_MO01C6JVM
Eko_XU02IKBWI
Erk_TZ02F55UR
Erk_TZ02F49KR
Eko_XU02IWU1N
Erk_TZ02IB1PY
Erk_TZ02I41K8
C07_2236669313
Eko_XU02F9E87
Erk_TZ02HGIZY
Erk_TZ02FQN1M
C_M09_2236659120
DSu_QV01B0T40
DSu_QV01EBPNU
DSu_QV01BRNFL
DSu_QV01COUEV
DSu_QV02GCMZ7
DSu_QV02G9CCU
DSu_QV02GR0RB
DSu_QV02F3Z29
C_P20_2246770179
98
76
64
52
97
70
60
59
58
50
61
59
55
72
59
79
64
61
62
63
0.02

86 ftsA

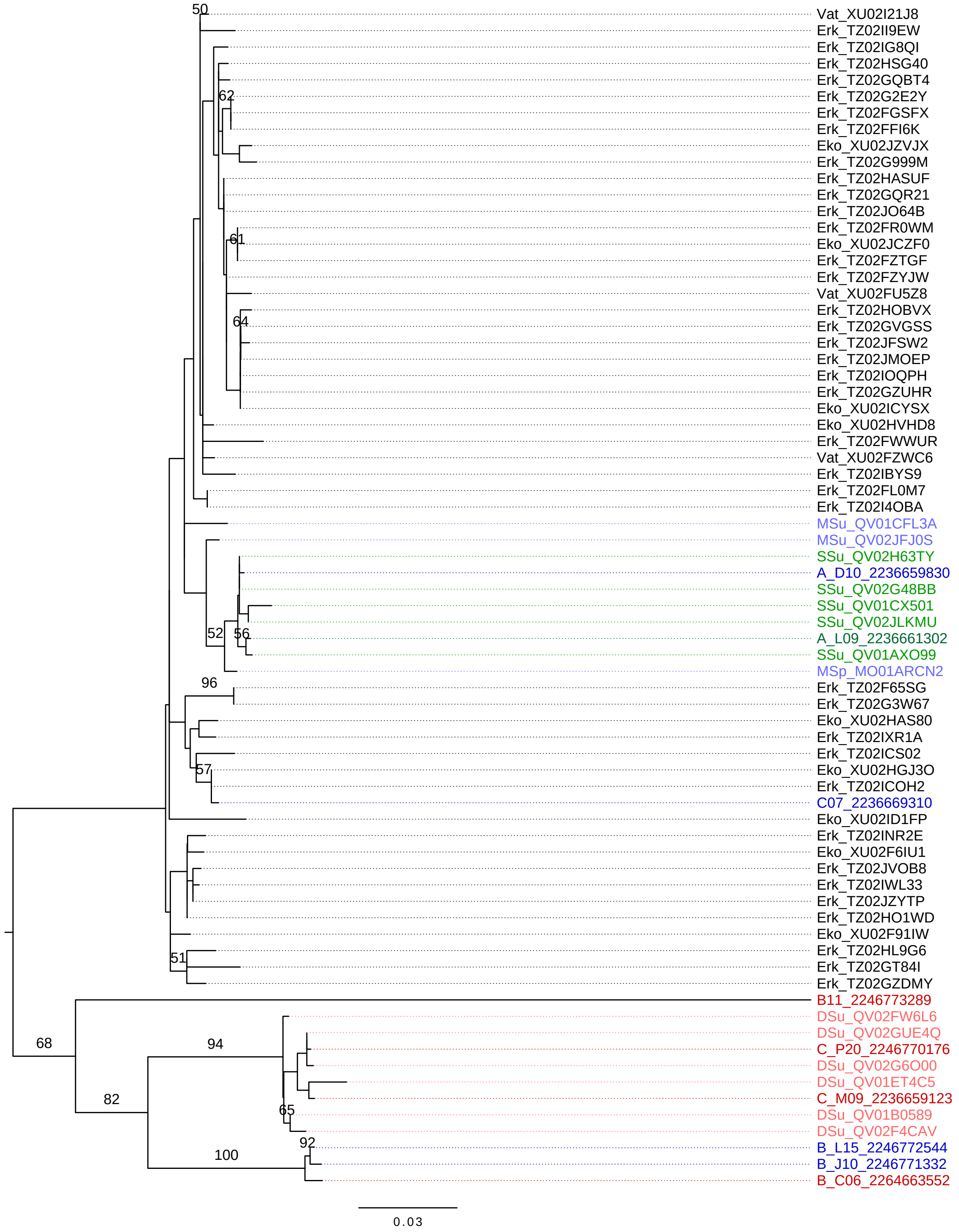

50
Vat_XU02I21J8
Erk_TZ02II9EW
Erk_TZ02IG8QI
Erk_TZ02HSG40
Erk_TZ02GQBT4
62
Erk_TZ02G2E2Y
Erk_TZ02FGSFX
Erk_TZ02FFI6K
Eko_XU02JZVJX
Erk_TZ02G999M
Erk_TZ02HASUF
Erk_TZ02GQR21
Erk_TZ02JO64B
Erk_TZ02FR0WM
61
Eko_XU02JCZF0
Erk_TZ02FZTGF
Erk_TZ02FZYJW
Vat_XU02FU5Z8
Erk_TZ02HOBVX
64
Erk_TZ02GVGSS
Erk_TZ02JFSW2
Erk_TZ02JMOEP
Erk_TZ02IOQPH
Erk_TZ02GZUHR
Eko_XU02ICYSX
Eko_XU02HVHD8
Erk_TZ02FWWUR
Vat_XU02FZWC6
Erk_TZ02IBYS9
Erk_TZ02FL0M7
Erk_TZ02I4OBA
MSu_QV01CFL3A
MSu_QV02JFJ0S
SSu_QV02H63TY
A_D10_2236659830
SSu_QV02G48BB
SSu_QV01CX501
SSu_QV02JLKMU
52
56
A_L09_2236661302
SSu_QV01AXO99
MSp_MO01ARCN2
96
Erk_TZ02F65SG
Erk_TZ02G3W67
Eko_XU02HAS80
Erk_TZ02IXR1A
Erk_TZ02ICS02
57
Eko_XU02HGJ3O
Erk_TZ02ICOH2
C07_2236669310
Eko_XU02ID1FP
Erk_TZ02INR2E
Eko_XU02F6IU1
Erk_TZ02JVOB8
Erk_TZ02IWL33
Erk_TZ02JZYTP
Erk_TZ02HO1WD
Eko_XU02F91IW
51
Erk_TZ02HL9G6
Erk_TZ02GT84I
Erk_TZ02GZDMY
B11_2246773289
DSu_QV02FW6L6
68
94
DSu_QV02GUE4Q
C_P20_2246770176
DSu_QV02G6O00
DSu_QV01ET4C5
82
C_M09_2236659123
65
DSu_QV01B0589
DSu_QV02F4CAV
92
B_L15_2246772544
100
B_J10_2246771332
B_C06_2264663552
0.03

92 sucC

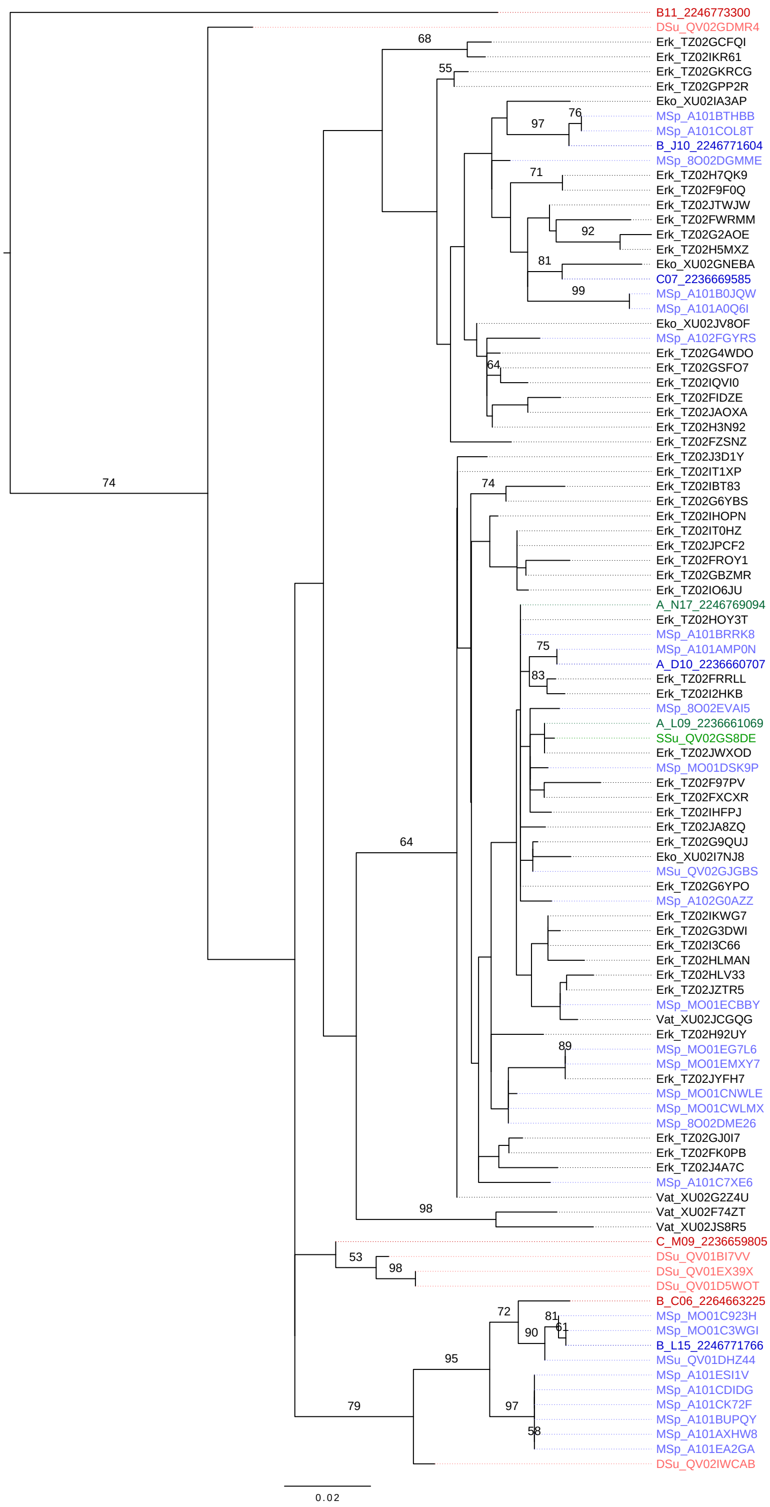
B11_2246773300
DSu_QV02GDMR4
Erk_TZ02GCFQI
Erk_TZ02IKR61
Erk_TZ02GKRCG
Erk_TZ02GPP2R
Eko_XU02IA3AP
MSp_A101BTHBB
MSp_A101COL8T
B_J10_2246771604
MSp_8O02DGMME
Erk_TZ02H7QK9
Erk_TZ02F9F0Q
Erk_TZ02JTWJW
Erk_TZ02FWRMM
Erk_TZ02G2AOE
Erk_TZ02H5MXZ
Eko_XU02GNEBA
C07_2236669585
MSp_A101B0JQW
MSp_A101A0Q6I
Eko_XU02JV8OF
MSp_A102FGYRS
Erk_TZ02G4WDO
Erk_TZ02GSFO7
Erk_TZ02IQVI0
Erk_TZ02FIDZE
Erk_TZ02JAOXA
Erk_TZ02H3N92
Erk_TZ02FZSNZ
Erk_TZ02J3D1Y
Erk_TZ02IT1XP
Erk_TZ02IBT83
Erk_TZ02G6YBS
Erk_TZ02IHOPN
Erk_TZ02IT0HZ
Erk_TZ02JPCF2
Erk_TZ02FROY1
Erk_TZ02GBZMR
Erk_TZ02IO6JU
A_N17_2246769094
Erk_TZ02HOY3T
MSp_A101BRRK8
MSp_A101AMP0N
A_D10_2236660707
Erk_TZ02FRRLL
Erk_TZ02I2HKB
MSp_8O02EVAI5
A_L09_2236661069
SSu_QV02GS8DE
Erk_TZ02JWXOD
MSp_MO01DSK9P
Erk_TZ02F97PV
Erk_TZ02FXCXR
Erk_TZ02IHFPJ
Erk_TZ02JA8ZQ
Erk_TZ02G9QUJ
Eko_XU02I7NJ8
MSu_QV02GJGBS
Erk_TZ02G6YPO
MSp_A102G0AZZ
Erk_TZ02IKWG7
Erk_TZ02G3DWI
Erk_TZ02I3C66
Erk_TZ02HLMAN
Erk_TZ02HLV33
Erk_TZ02JZTR5
MSp_MO01ECBBY
Vat_XU02JCGQG
Erk_TZ02H92UY
MSp_MO01EG7L6
MSp_MO01EMXY7
Erk_TZ02JYFH7
MSp_MO01CNWLE
MSp_MO01CWLMX
MSp_8O02DME26
Erk_TZ02GJ0I7
Erk_TZ02FK0PB
Erk_TZ02J4A7C
MSp_A101C7XE6
Vat_XU02G2Z4U
Vat_XU02F74ZT
Vat_XU02JS8R5
C_M09_2236659805
DSu_QV01BI7VV
DSu_QV01EX39X
DSu_QV01D5WOT
B_C06_2264663225
MSp_MO01C923H
MSp_MO01C3WGI
B_L15_2246771766
MSu_QV01DHZ44
MSp_A101ESI1V
MSp_A101CDIDG
MSp_A101CK72F
MSp_A101BUPQY
MSp_A101AXHW8
MSp_A101EA2GA
DSu_QV02IWCAB
0.02

93 leuS

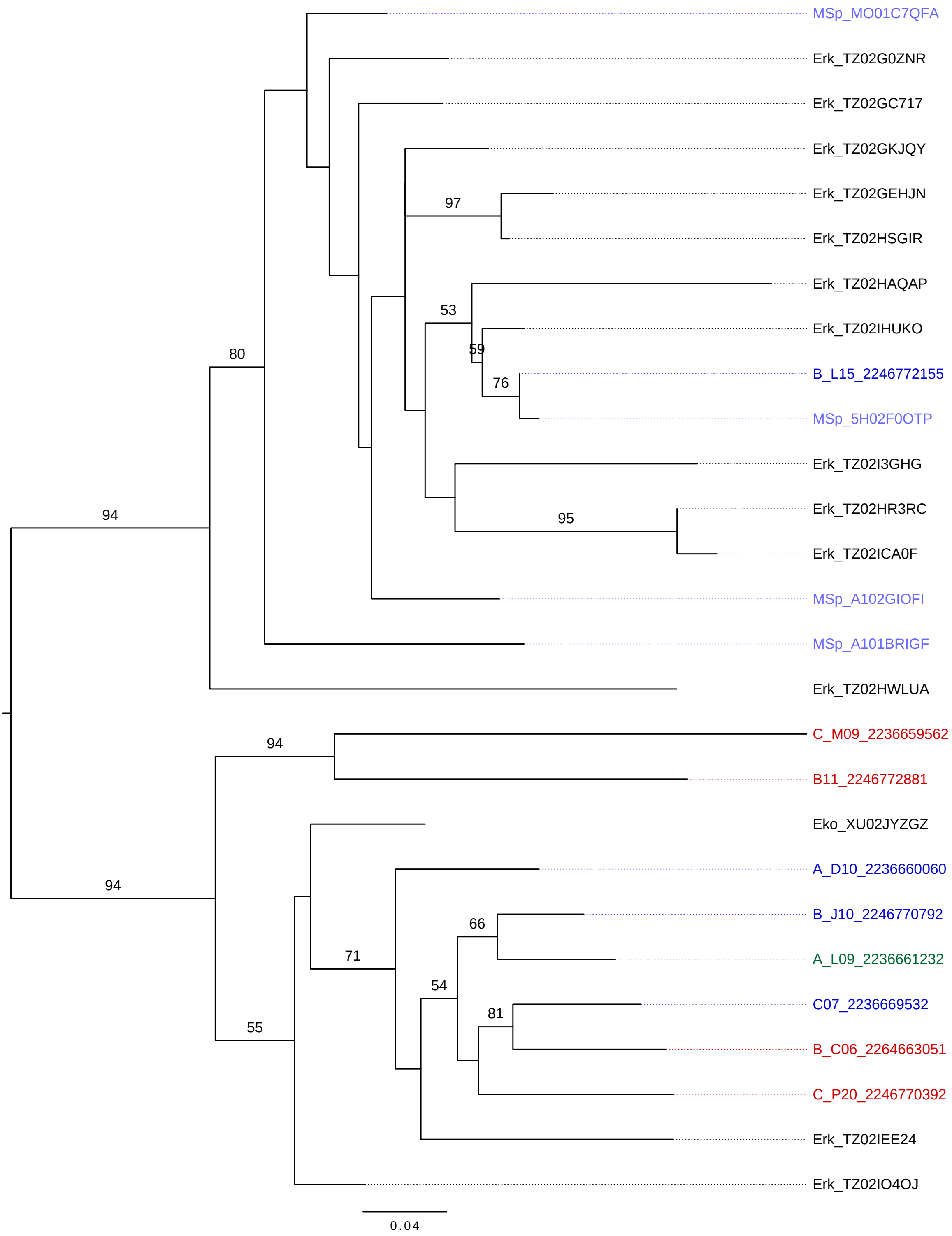

MSp_MO01C7QFA
Erk_TZ02G0ZNR
Erk_TZ02GC717
Erk_TZ02GKJQY
Erk_TZ02GEHJN
Erk_TZ02HSGIR
Erk_TZ02HAQAP
Erk_TZ02IHUKO
B_L15_2246772155
MSp_5H02F0OTP
Erk_TZ02I3GHG
Erk_TZ02HR3RC
Erk_TZ02ICA0F
MSp_A102GIOFI
MSp_A101BRIGF
Erk_TZ02HWLUA
C_M09_2236659562
B11_2246772881
Eko_XU02JYZGZ
A_D10_2236660060
B_J10_2246770792
A_L09_2236661232
C07_2236669532
B_C06_2264663051
C_P20_2246770392
Erk_TZ02IEE24
Erk_TZ02IO4OJ
97
53
59
76
95
80
94
94
94
66
71
54
81
55
0.04

94 gidA

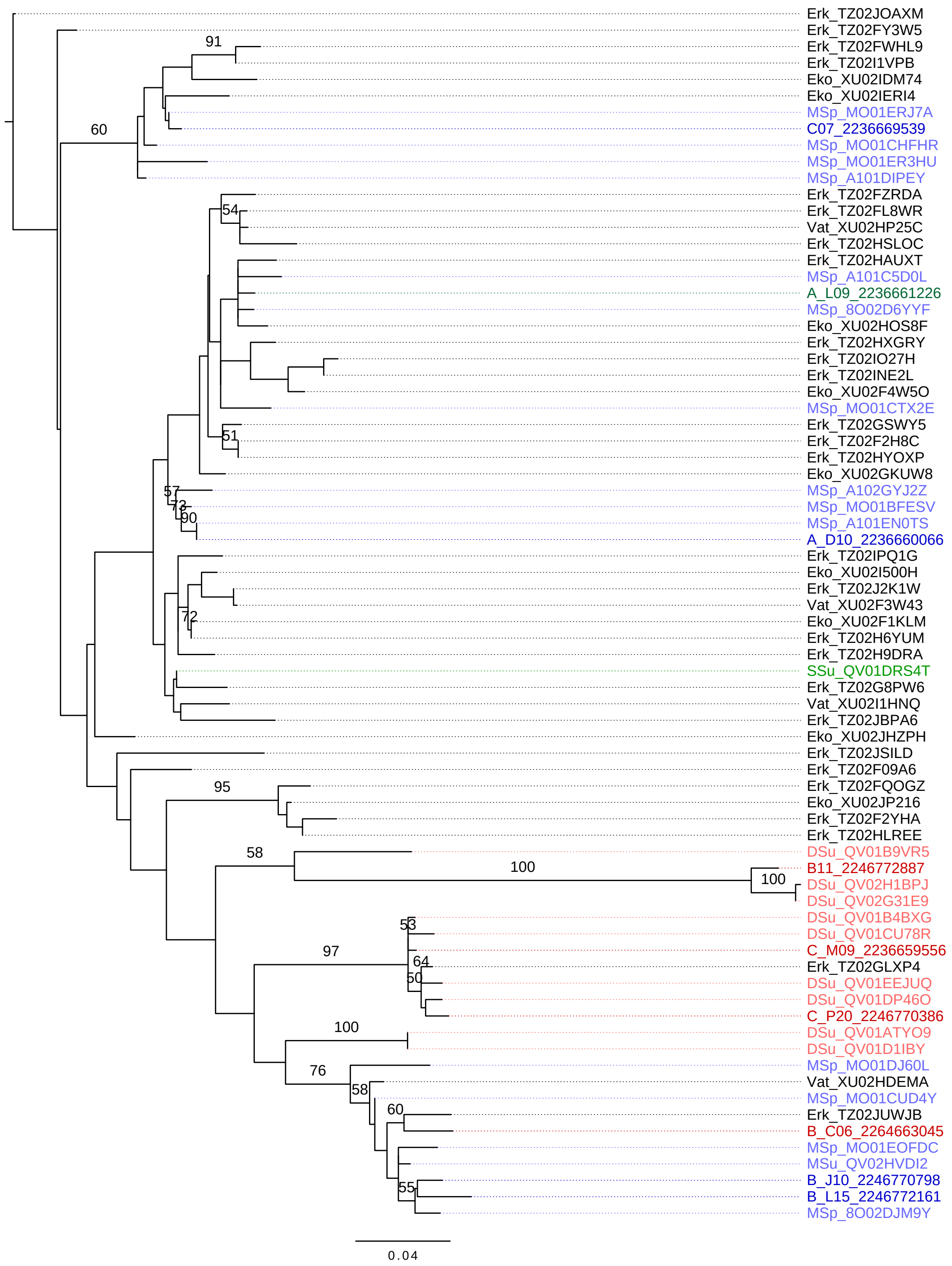
Erk_TZ02JOAXM
Erk_TZ02FY3W5
Erk_TZ02FWHL9
Erk_TZ02I1VPB
Eko_XU02IDM74
Eko_XU02IERI4
MSp_MO01ERJ7A
C07_2236669539
MSp_MO01CHFHR
MSp_MO01ER3HU
MSp_A101DIPEY
Erk_TZ02FZRDA
Erk_TZ02FL8WR
Vat_XU02HP25C
Erk_TZ02HSLOC
Erk_TZ02HAUXT
MSp_A101C5D0L
A_L09_2236661226
MSp_8O02D6YYF
Eko_XU02HOS8F
Erk_TZ02HXGRY
Erk_TZ02IO27H
Erk_TZ02INE2L
Eko_XU02F4W5O
MSp_MO01CTX2E
Erk_TZ02GSWY5
Erk_TZ02F2H8C
Erk_TZ02HYOXP
Eko_XU02GKUW8
MSp_A102GYJ2Z
MSp_MO01BFESV
MSp_A101EN0TS
A_D10_2236660066
Erk_TZ02IPQ1G
Eko_XU02I500H
Erk_TZ02J2K1W
Vat_XU02F3W43
Eko_XU02F1KLM
Erk_TZ02H6YUM
Erk_TZ02H9DRA
SSu_QV01DRS4T
Erk_TZ02G8PW6
Vat_XU02I1HNQ
Erk_TZ02JBPA6
Eko_XU02JHZPH
Erk_TZ02JSILD
Erk_TZ02F09A6
Erk_TZ02FQOGZ
Eko_XU02JP216
Erk_TZ02F2YHA
Erk_TZ02HLREE
DSu_QV01B9VR5
B11_2246772887
DSu_QV02H1BPJ
DSu_QV02G31E9
DSu_QV01B4BXG
DSu_QV01CU78R
C_M09_2236659556
Erk_TZ02GLXP4
DSu_QV01EEJUQ
DSu_QV01DP46O
C_P20_2246770386
DSu_QV01ATYO9
DSu_QV01D1IBY
MSp_MO01DJ60L
Vat_XU02HDEMA
MSp_MO01CUD4Y
Erk_TZ02JUWJB
B_C06_2264663045
MSp_MO01EOFDC
MSu_QV02HVDI2
B_J10_2246770798
B_L15_2246772161
MSp_8O02DJM9Y
91
60
54
51
57
73
90
72
95
58
100
100
53
97
64
50
100
76
58
60
55
0.04

95 trmE

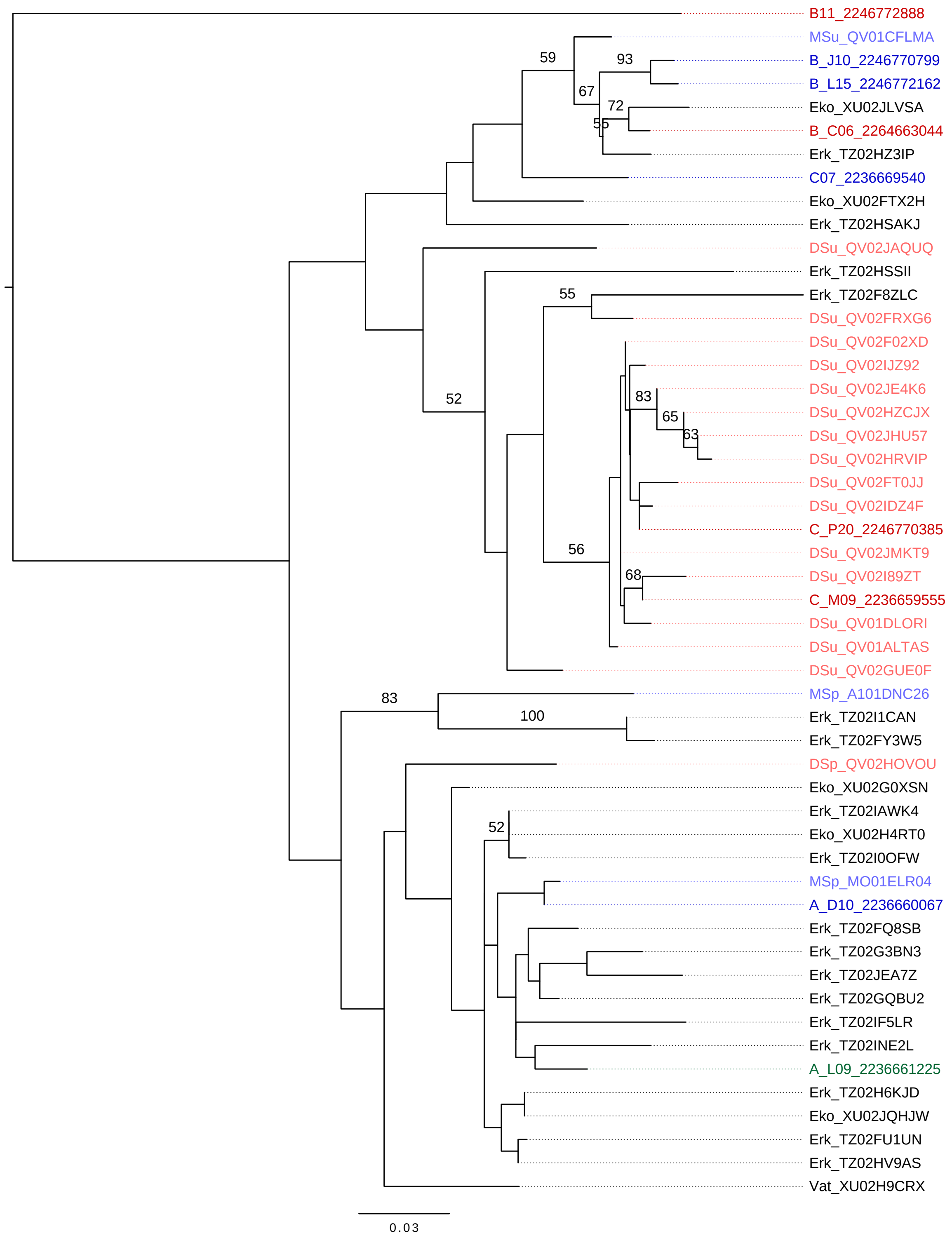
B11_2246772888
MSu_QV01CFLMA
B_J10_2246770799
B_L15_2246772162
Eko_XU02JLVSA
B_C06_2264663044
Erk_TZ02HZ3IP
C07_2236669540
Eko_XU02FTX2H
Erk_TZ02HSAKJ
DSu_QV02JAQUQ
Erk_TZ02HSSII
Erk_TZ02F8ZLC
DSu_QV02FRXG6
DSu_QV02F02XD
DSu_QV02IJZ92
DSu_QV02JE4K6
DSu_QV02HZCJX
DSu_QV02JHU57
DSu_QV02HRVIP
DSu_QV02FT0JJ
DSu_QV02IDZ4F
C_P20_2246770385
DSu_QV02JMKT9
DSu_QV02I89ZT
C_M09_2236659555
DSu_QV01DLORI
DSu_QV01ALTAS
DSu_QV02GUE0F
MSp_A101DNC26
Erk_TZ02I1CAN
Erk_TZ02FY3W5
DSp_QV02HOVOU
Eko_XU02G0XSN
Erk_TZ02IAWK4
Eko_XU02H4RT0
Erk_TZ02I0OFW
MSp_MO01ELR04
A_D10_2236660067
Erk_TZ02FQ8SB
Erk_TZ02G3BN3
Erk_TZ02JEA7Z
Erk_TZ02GQBU2
Erk_TZ02IF5LR
Erk_TZ02INE2L
A_L09_2236661225
Erk_TZ02H6KJD
Eko_XU02JQHJW
Erk_TZ02FU1UN
Erk_TZ02HV9AS
Vat_XU02H9CRX
59
93
67
72
55
55
52
83
65
63
56
68
83
100
52
0.03

102 dnaN

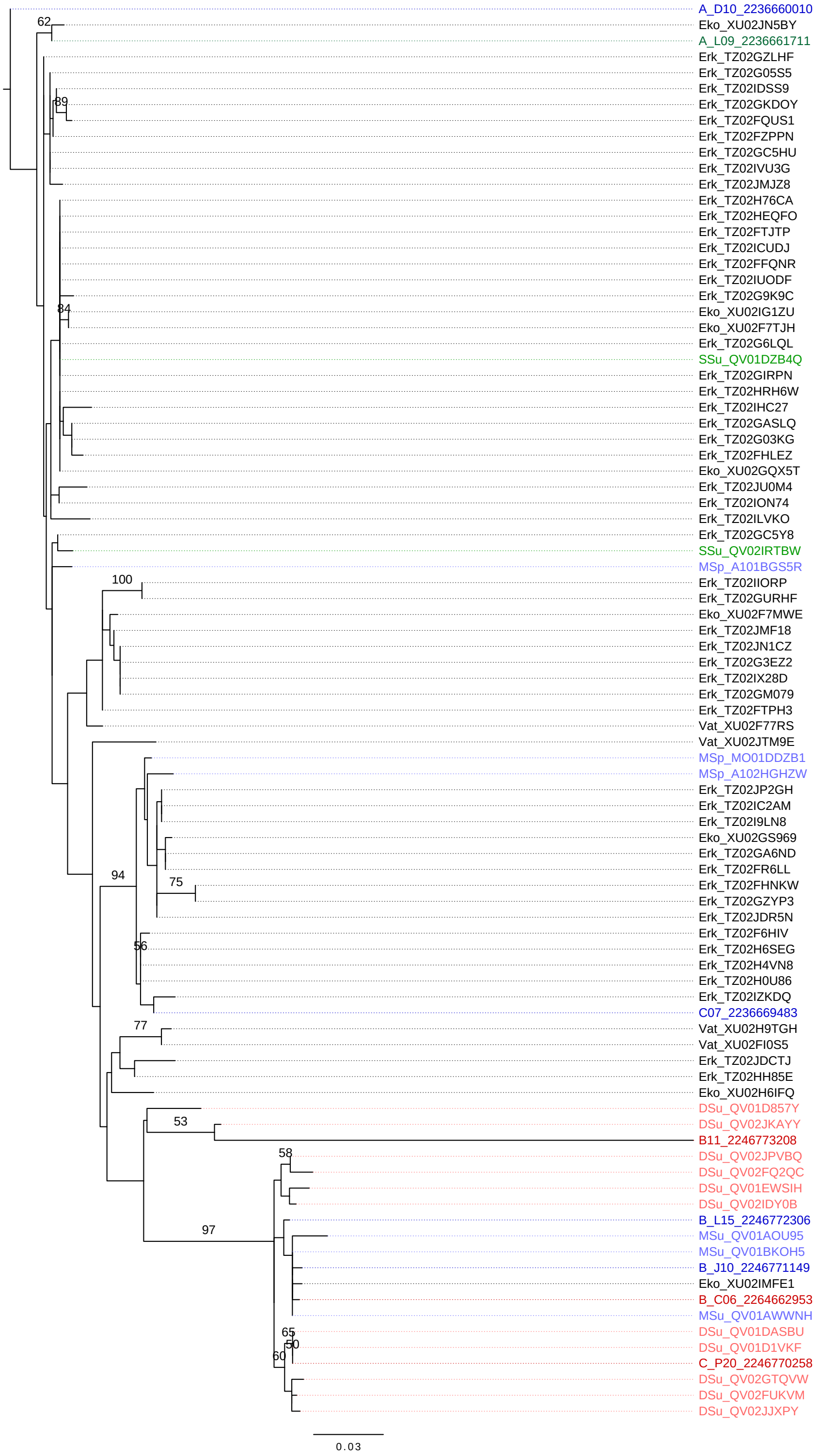
A_D10_2236660010
Eko_XU02JN5BY
A_L09_2236661711
Erk_TZ02GZLHF
Erk_TZ02G05S5
Erk_TZ02IDSS9
Erk_TZ02GKDOY
Erk_TZ02FQUS1
Erk_TZ02FZPPN
Erk_TZ02GC5HU
Erk_TZ02IVU3G
Erk_TZ02JMJZ8
Erk_TZ02H76CA
Erk_TZ02HEQFO
Erk_TZ02FTJTP
Erk_TZ02ICUDJ
Erk_TZ02FFQNR
Erk_TZ02IUODF
Erk_TZ02G9K9C
Eko_XU02IG1ZU
Eko_XU02F7TJH
Erk_TZ02G6LQL
SSu_QV01DZB4Q
Erk_TZ02GIRPN
Erk_TZ02HRH6W
Erk_TZ02IHC27
Erk_TZ02GASLQ
Erk_TZ02G03KG
Erk_TZ02FHLEZ
Eko_XU02GQX5T
Erk_TZ02JU0M4
Erk_TZ02ION74
Erk_TZ02ILVKO
Erk_TZ02GC5Y8
SSu_QV02IRTBW
MSp_A101BGS5R
Erk_TZ02IIORP
Erk_TZ02GURHF
Eko_XU02F7MWE
Erk_TZ02JMF18
Erk_TZ02JN1CZ
Erk_TZ02G3EZ2
Erk_TZ02IX28D
Erk_TZ02GM079
Erk_TZ02FTPH3
Vat_XU02F77RS
Vat_XU02JTM9E
MSp_MO01DDZB1
MSp_A102HGHZW
Erk_TZ02JP2GH
Erk_TZ02IC2AM
Erk_TZ02I9LN8
Eko_XU02GS969
Erk_TZ02GA6ND
Erk_TZ02FR6LL
Erk_TZ02FHNKW
Erk_TZ02GZYP3
Erk_TZ02JDR5N
Erk_TZ02F6HIV
Erk_TZ02H6SEG
Erk_TZ02H4VN8
Erk_TZ02H0U86
Erk_TZ02IZKDQ
C07_2236669483
Vat_XU02H9TGH
Vat_XU02FI0S5
Erk_TZ02JDCTJ
Erk_TZ02HH85E
Eko_XU02H6IFQ
DSu_QV01D857Y
DSu_QV02JKAYY
B11_2246773208
DSu_QV02JPVBQ
DSu_QV02FQ2QC
DSu_QV01EWSIH
DSu_QV02IDY0B
B_L15_2246772306
MSu_QV01AOU95
MSu_QV01BKOH5
B_J10_2246771149
Eko_XU02IMFE1
B_C06_2264662953
MSu_QV01AWWNH
DSu_QV01DASBU
DSu_QV01D1VKF
C_P20_2246770258
DSu_QV02GTQVW
DSu_QV02FUKVM
DSu_QV02JJXPY
62
89
84
100
94
75
56
77
53
58
97
65
50
60
0.03

104 dnaA

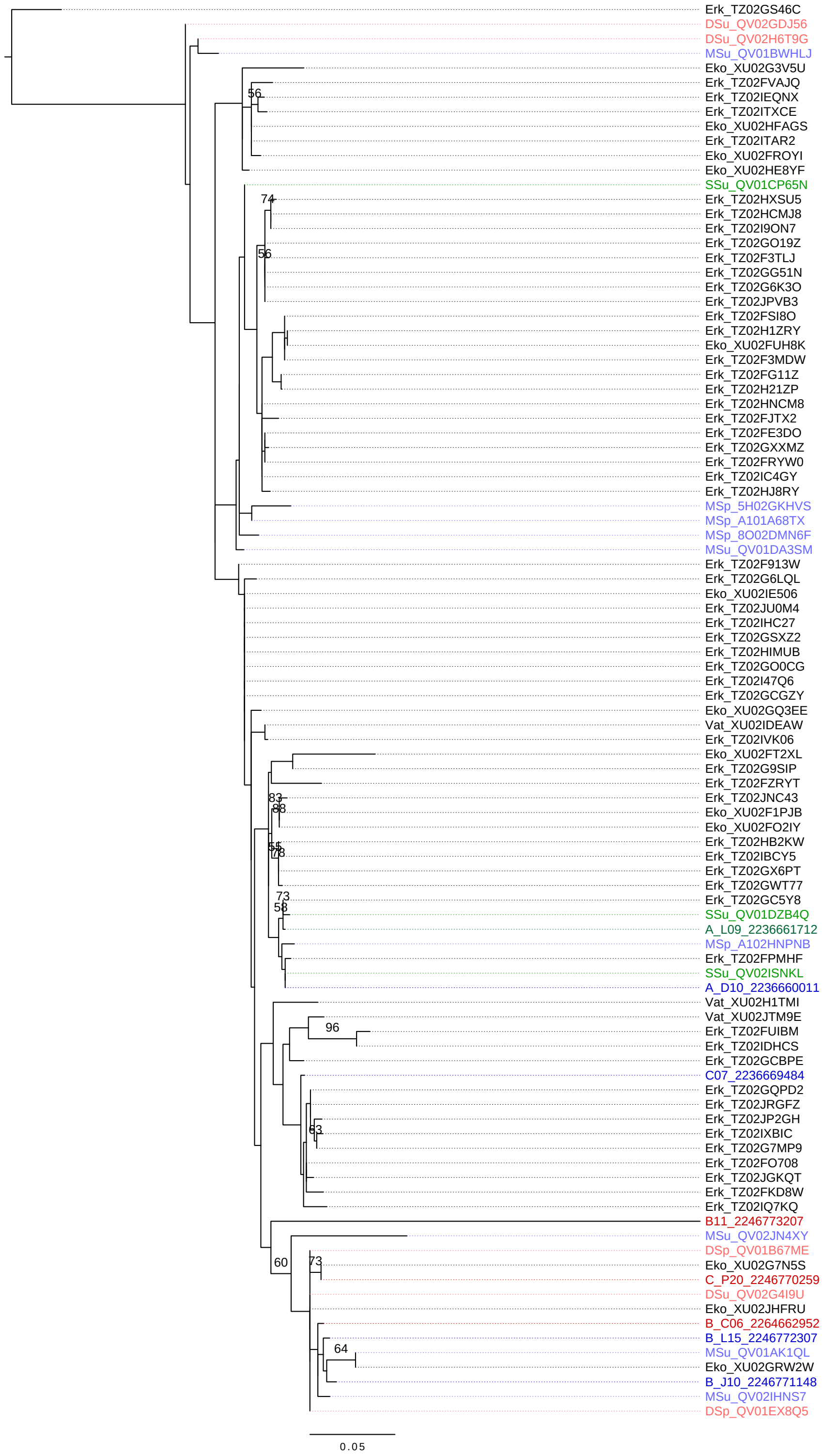
Erk_TZ02GS46C
DSu_QV02GDJ56
DSu_QV02H6T9G
MSu_QV01BWHLJ
Eko_XU02G3V5U
Erk_TZ02FVAJQ
Erk_TZ02IEQNX
Erk_TZ02ITXCE
Eko_XU02HFAGS
Erk_TZ02ITAR2
Eko_XU02FROYI
Eko_XU02HE8YF
SSu_QV01CP65N
Erk_TZ02HXSU5
Erk_TZ02HCMJ8
Erk_TZ02I9ON7
Erk_TZ02GO19Z
Erk_TZ02F3TLJ
Erk_TZ02GG51N
Erk_TZ02G6K3O
Erk_TZ02JPVB3
Erk_TZ02FSI8O
Erk_TZ02H1ZRY
Eko_XU02FUH8K
Erk_TZ02F3MDW
Erk_TZ02FG11Z
Erk_TZ02H21ZP
Erk_TZ02HNCM8
Erk_TZ02FJTX2
Erk_TZ02FE3DO
Erk_TZ02GXXMZ
Erk_TZ02FRYW0
Erk_TZ02IC4GY
Erk_TZ02HJ8RY
MSp_5H02GKHVS
MSp_A101A68TX
MSp_8O02DMN6F
MSu_QV01DA3SM
Erk_TZ02F913W
Erk_TZ02G6LQL
Eko_XU02IE506
Erk_TZ02JU0M4
Erk_TZ02IHC27
Erk_TZ02GSXZ2
Erk_TZ02HIMUB
Erk_TZ02GO0CG
Erk_TZ02I47Q6
Erk_TZ02GCGZY
Eko_XU02GQ3EE
Vat_XU02IDEAW
Erk_TZ02IVK06
Eko_XU02FT2XL
Erk_TZ02G9SIP
Erk_TZ02FZRYT
Erk_TZ02JNC43
Eko_XU02F1PJB
Eko_XU02FO2IY
Erk_TZ02HB2KW
Erk_TZ02IBCY5
Erk_TZ02GX6PT
Erk_TZ02GWT77
Erk_TZ02GC5Y8
SSu_QV01DZB4Q
A_L09_2236661712
MSp_A102HNPNB
Erk_TZ02FPMHF
SSu_QV02ISNKL
A_D10_2236660011
Vat_XU02H1TMI
Vat_XU02JTM9E
Erk_TZ02FUIBM
Erk_TZ02IDHCS
Erk_TZ02GCBPE
C07_2236669484
Erk_TZ02GQPD2
Erk_TZ02JRGFZ
Erk_TZ02JP2GH
Erk_TZ02IXBIC
Erk_TZ02G7MP9
Erk_TZ02FO708
Erk_TZ02JGKQT
Erk_TZ02FKD8W
Erk_TZ02IQ7KQ
B11_2246773207
MSu_QV02JN4XY
DSp_QV01B67ME
Eko_XU02G7N5S
C_P20_2246770259
DSu_QV02G4I9U
Eko_XU02JHFRU
B_C06_2264662952
B_L15_2246772307
MSu_QV01AK1QL
Eko_XU02GRW2W
B_J10_2246771148
MSu_QV02IHNS7
DSp_QV01EX8Q5
56
74
56
83
88
55
78
73
58
96
63
60
73
64
0.05

122 recA

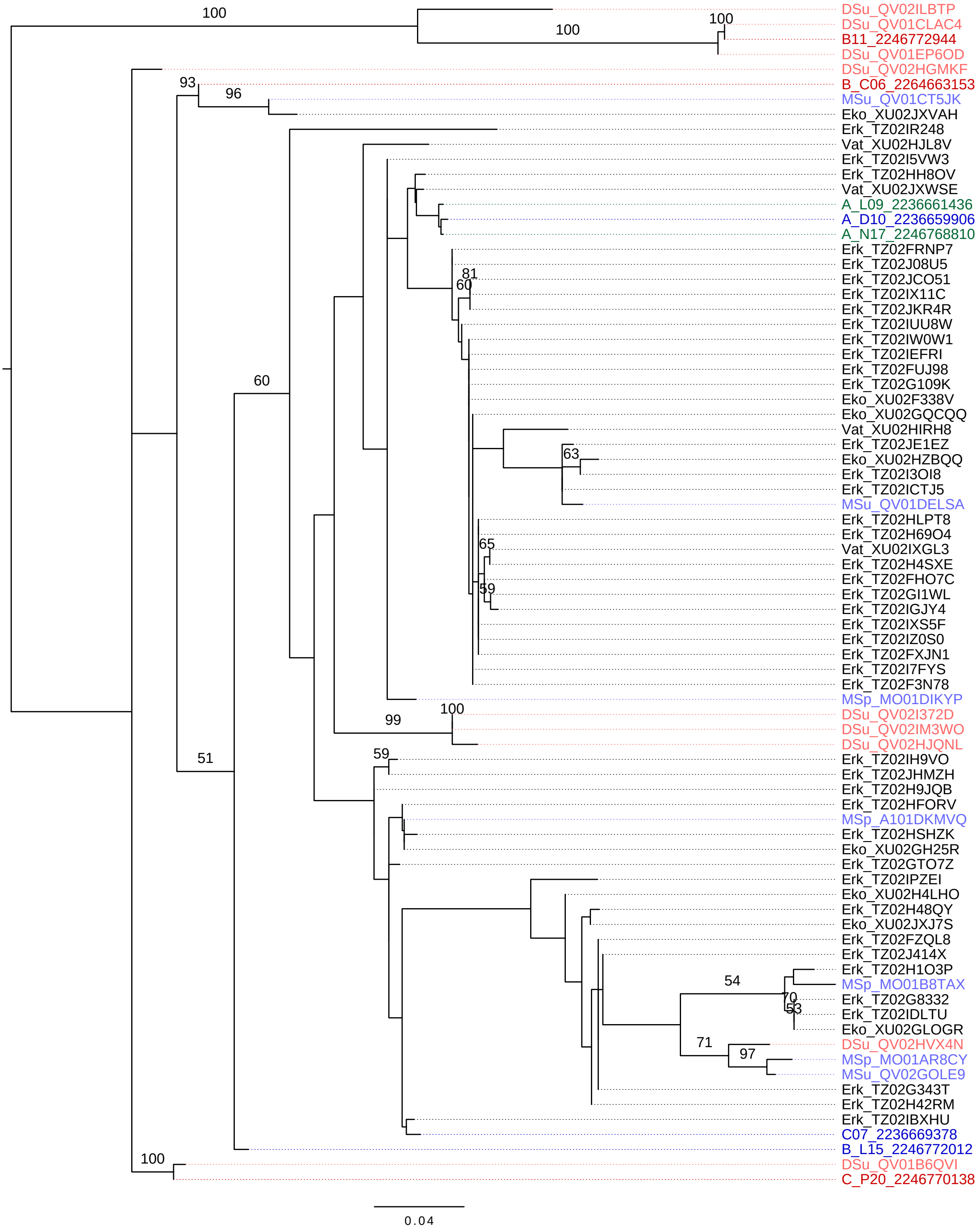

DSu_QV02ILBTP
DSu_QV01CLAC4
B11_2246772944
DSu_QV01EP6OD
DSu_QV02HGMKF
B_C06_2264663153
MSu_QV01CT5JK
Eko_XU02JXVAH
Erk_TZ02IR248
Vat_XU02HJL8V
Erk_TZ02I5VW3
Erk_TZ02HH8OV
Vat_XU02JXWSE
A_L09_2236661436
A_D10_2236659906
A_N17_2246768810
Erk_TZ02FRNP7
Erk_TZ02J08U5
Erk_TZ02JCO51
Erk_TZ02IX11C
Erk_TZ02JKR4R
Erk_TZ02IUU8W
Erk_TZ02IW0W1
Erk_TZ02IEFRI
Erk_TZ02FUJ98
Erk_TZ02G109K
Eko_XU02F338V
Eko_XU02GQCQQ
Vat_XU02HIRH8
Erk_TZ02JE1EZ
Eko_XU02HZBQQ
Erk_TZ02I3OI8
Erk_TZ02ICTJ5
MSu_QV01DELSA
Erk_TZ02HLPT8
Erk_TZ02H69O4
Vat_XU02IXGL3
Erk_TZ02H4SXE
Erk_TZ02FHO7C
Erk_TZ02GI1WL
Erk_TZ02IGJY4
Erk_TZ02IXS5F
Erk_TZ02IZ0S0
Erk_TZ02FXJN1
Erk_TZ02I7FYS
Erk_TZ02F3N78
MSp_MO01DIKYP
DSu_QV02I372D
DSu_QV02IM3WO
DSu_QV02HJQNL
Erk_TZ02IH9VO
Erk_TZ02JHMZH
Erk_TZ02H9JQB
Erk_TZ02HFORV
MSp_A101DKMVQ
Erk_TZ02HSHZK
Eko_XU02GH25R
Erk_TZ02GTO7Z
Erk_TZ02IPZEI
Eko_XU02H4LHO
Erk_TZ02H48QY
Eko_XU02JXJ7S
Erk_TZ02FZQL8
Erk_TZ02J414X
Erk_TZ02H1O3P
MSp_MO01B8TAX
Erk_TZ02G8332
Erk_TZ02IDLTU
Eko_XU02GLOGR
DSu_QV02HVX4N
MSp_MO01AR8CY
MSu_QV02GOLE9
Erk_TZ02G343T
Erk_TZ02H42RM
Erk_TZ02IBXHU
C07_2236669378
B_L15_2246772012
DSu_QV01B6QVI
C_P20_2246770138
100
100
100
93
96
81
60
60
63
65
59
100
99
59
51
54
70
53
71
97
100
0.04

127 atpD

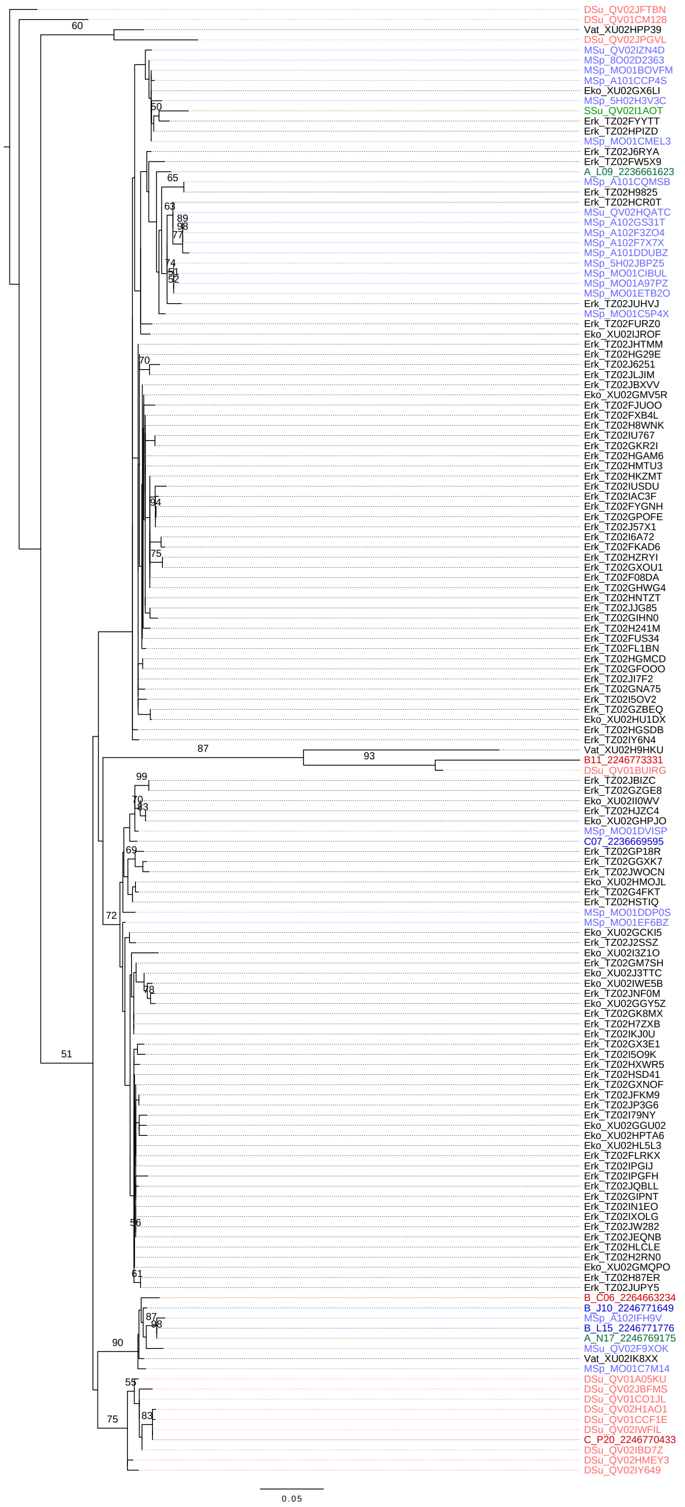

DSu_QV02JFTBN
DSu_QV01CM128
Vat_XU02HPP39
DSu_QV02JPGVL
MSu_QV02IZN4D
MSp_8O02D2363
MSp_MO01BOVFM
MSp_A101CCP4S
Eko_XU02GX6LI
MSp_5H02H3V3C
SSu_QV02I1AOT
Erk_TZ02FYYTT
Erk_TZ02HPIZD
MSp_MO01CMEL3
Erk_TZ02J6RYA
Erk_TZ02FW5X9
A_L09_2236661623
MSp_A101CQMSB
Erk_TZ02H9825
Erk_TZ02HCR0T
MSu_QV02HQATC
MSp_A102GS31T
MSp_A102F3ZO4
MSp_A102F7X7X
MSp_A101DDUBZ
MSp_5H02JBPZ5
MSp_MO01CIBUL
MSp_MO01A97PZ
MSp_MO01ETB2O
Erk_TZ02JUHVJ
MSp_MO01C5P4X
Erk_TZ02FURZ0
Eko_XU02IJROF
Erk_TZ02JHTMM
Erk_TZ02HG29E
Erk_TZ02J6251
Erk_TZ02JLJIM
Erk_TZ02JBXVV
Eko_XU02GMV5R
Erk_TZ02FJUOO
Erk_TZ02FXB4L
Erk_TZ02H8WNK
Erk_TZ02IU767
Erk_TZ02GKR2I
Erk_TZ02HGAM6
Erk_TZ02HMTU3
Erk_TZ02HKZMT
Erk_TZ02IUSDU
Erk_TZ02IAC3F
Erk_TZ02FYGNH
Erk_TZ02GPOFE
Erk_TZ02J57X1
Erk_TZ02I6A72
Erk_TZ02FKAD6
Erk_TZ02HZRYI
Erk_TZ02GXOU1
Erk_TZ02F08DA
Erk_TZ02GHWG4
Erk_TZ02HNTZT
Erk_TZ02JJG85
Erk_TZ02GIHN0
Erk_TZ02H241M
Erk_TZ02FUS34
Erk_TZ02FL1BN
Erk_TZ02HGMCD
Erk_TZ02GFOOO
Erk_TZ02JI7F2
Erk_TZ02GNA75
Erk_TZ02I5OV2
Erk_TZ02GZBEQ
Eko_XU02HU1DX
Erk_TZ02HGSDB
Erk_TZ02IY6N4
Vat_XU02H9HKU
B11_2246773331
DSu_QV01BUIRG
Erk_TZ02JBIZC
Erk_TZ02GZGE8
Eko_XU02II0WV
Erk_TZ02HJZC4
Eko_XU02GHPJO
MSp_MO01DVISP
C07_2236669595
Erk_TZ02GP18R
Erk_TZ02GGXK7
Erk_TZ02JWOCN
Eko_XU02HMOJL
Erk_TZ02G4FKT
Erk_TZ02HSTIQ
MSp_MO01DDP0S
MSp_MO01EF6BZ
Eko_XU02GCKI5
Erk_TZ02J2SSZ
Eko_XU02I3Z1O
Erk_TZ02GM7SH
Eko_XU02J3TTC
Eko_XU02IWE5B
Erk_TZ02JNF0M
Eko_XU02GGY5Z
Erk_TZ02GK8MX
Erk_TZ02H7ZXB
Erk_TZ02IKJ0U
Erk_TZ02GX3E1
Erk_TZ02I5O9K
Erk_TZ02HXWR5
Erk_TZ02HSD41
Erk_TZ02GXNOF
Erk_TZ02JFKM9
Erk_TZ02JP3G6
Erk_TZ02I79NY
Eko_XU02GGU02
Eko_XU02HPTA6
Eko_XU02HL5L3
Erk_TZ02FLRKX
Erk_TZ02IPGIJ
Erk_TZ02IPGFH
Erk_TZ02JQBLL
Erk_TZ02GIPNT
Erk_TZ02IN1EO
Erk_TZ02IXOLG
Erk_TZ02JW282
Erk_TZ02JEQNB
Erk_TZ02HLCLE
Erk_TZ02H2RN0
Eko_XU02GMQPO
Erk_TZ02H87ER
Erk_TZ02JUPY5
B_C06_2264663234
B_J10_2246771649
MSp_A102IFH9V
B_L15_2246771776
A_N17_2246769175
MSu_QV02F9XOK
Vat_XU02IK8XX
MSp_MO01C7M14
DSu_QV01A05KU
DSu_QV02JBFMS
DSu_QV01CO1JL
DSu_QV02H1AO1
DSu_QV01CCF1E
DSu_QV02IWFIL
C_P20_2246770433
DSu_QV02IBD7Z
DSu_QV02HMEY3
DSu_QV02IY649
60
50
65
63
89
98
77
74
51
52
70
94
75
87
93
99
70
83
69
72
78
51
56
61
87
98
90
55
83
75
0.05

130 ubiH

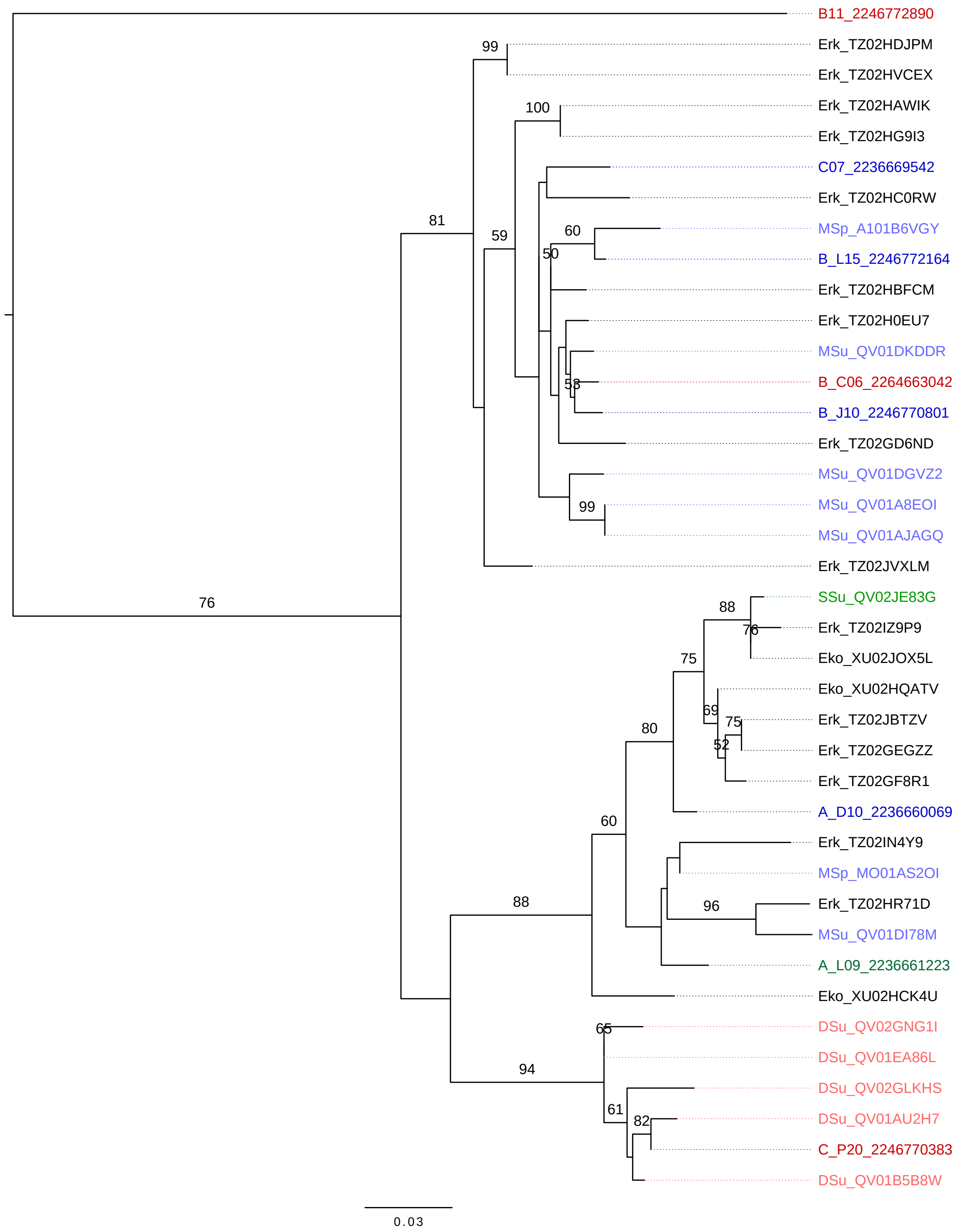
B11_2246772890
Erk_TZ02HDJPM
Erk_TZ02HVCEX
Erk_TZ02HAWIK
Erk_TZ02HG9I3
C07_2236669542
Erk_TZ02HC0RW
MSp_A101B6VGY
B_L15_2246772164
Erk_TZ02HBFCM
Erk_TZ02H0EU7
MSu_QV01DKDDR
B_C06_2264663042
B_J10_2246770801
Erk_TZ02GD6ND
MSu_QV01DGVZ2
MSu_QV01A8EOI
MSu_QV01AJAGQ
Erk_TZ02JVXLM
SSu_QV02JE83G
Erk_TZ02IZ9P9
Eko_XU02JOX5L
Eko_XU02HQATV
Erk_TZ02JBTZV
Erk_TZ02GEGZZ
Erk_TZ02GF8R1
A_D10_2236660069
Erk_TZ02IN4Y9
MSp_MO01AS2OI
Erk_TZ02HR71D
MSu_QV01DI78M
A_L09_2236661223
Eko_XU02HCK4U
DSu_QV02GNG1I
DSu_QV01EA86L
DSu_QV02GLKHS
DSu_QV01AU2H7
C_P20_2246770383
DSu_QV01B5B8W
99
100
81
59
60
50
58
99
76
88
76
75
69
75
52
80
60
96
88
65
94
61
82
0.03

162 recJ

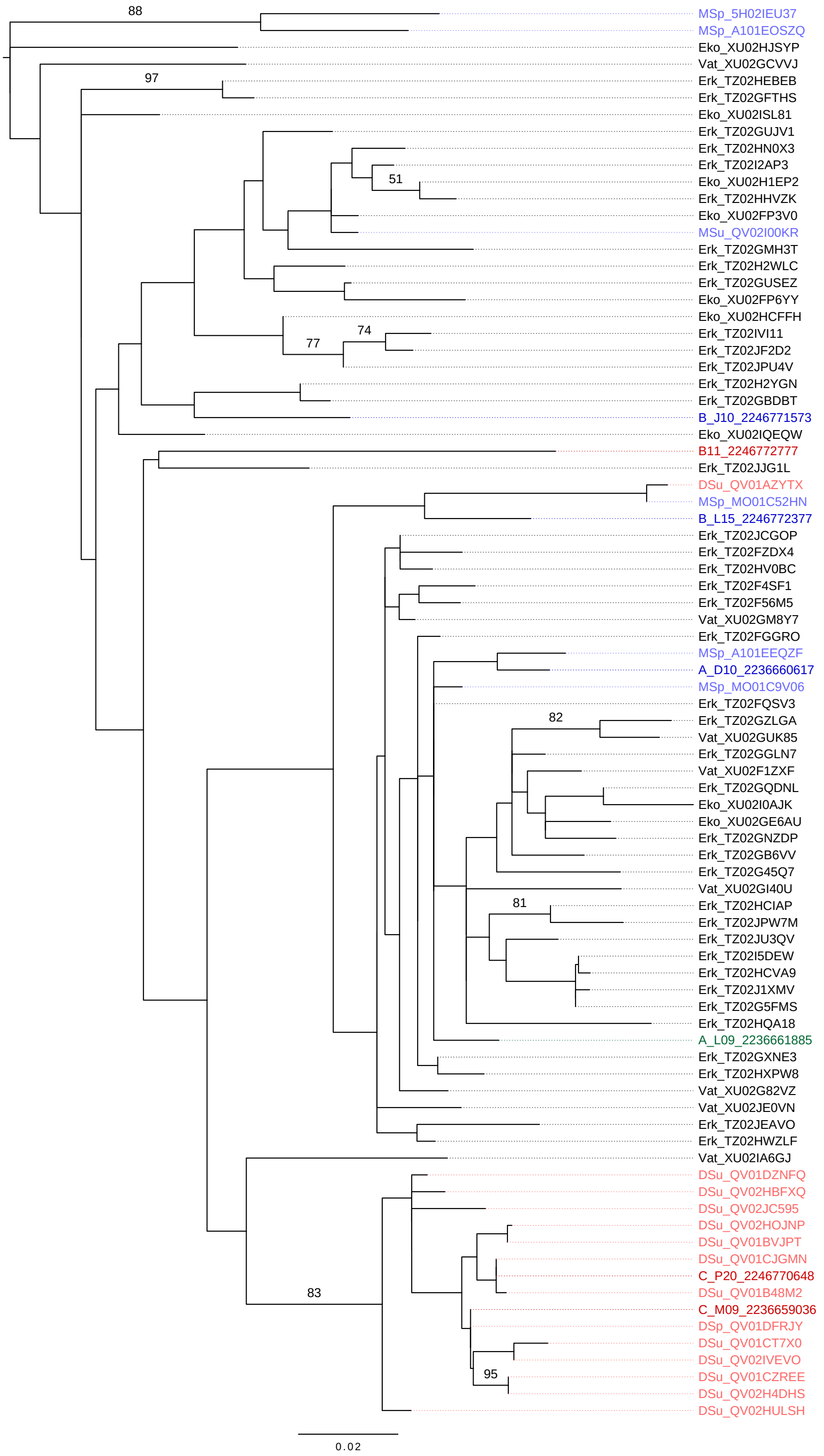
MSp_5H02IEU37
MSp_A101EOSZQ
Eko_XU02HJSYP
Vat_XU02GCVVJ
Erk_TZ02HEBEB
Erk_TZ02GFTHS
Eko_XU02ISL81
Erk_TZ02GUJV1
Erk_TZ02HN0X3
Erk_TZ02I2AP3
Eko_XU02H1EP2
Erk_TZ02HHVZK
Eko_XU02FP3V0
MSu_QV02I00KR
Erk_TZ02GMH3T
Erk_TZ02H2WLC
Erk_TZ02GUSEZ
Eko_XU02FP6YY
Eko_XU02HCFFH
Erk_TZ02IVI11
Erk_TZ02JF2D2
Erk_TZ02JPU4V
Erk_TZ02H2YGN
Erk_TZ02GBDBT
B_J10_2246771573
Eko_XU02IQEQW
B11_2246772777
Erk_TZ02JJG1L
DSu_QV01AZYTX
MSp_MO01C52HN
B_L15_2246772377
Erk_TZ02JCGOP
Erk_TZ02FZDX4
Erk_TZ02HV0BC
Erk_TZ02F4SF1
Erk_TZ02F56M5
Vat_XU02GM8Y7
Erk_TZ02FGGRO
MSp_A101EEQZF
A_D10_2236660617
MSp_MO01C9V06
Erk_TZ02FQSV3
Erk_TZ02GZLGA
Vat_XU02GUK85
Erk_TZ02GGLN7
Vat_XU02F1ZXF
Erk_TZ02GQDNL
Eko_XU02I0AJK
Eko_XU02GE6AU
Erk_TZ02GNZDP
Erk_TZ02GB6VV
Erk_TZ02G45Q7
Vat_XU02GI40U
Erk_TZ02HCIAP
Erk_TZ02JPW7M
Erk_TZ02JU3QV
Erk_TZ02I5DEW
Erk_TZ02HCVA9
Erk_TZ02J1XMV
Erk_TZ02G5FMS
Erk_TZ02HQA18
A_L09_2236661885
Erk_TZ02GXNE3
Erk_TZ02HXPW8
Vat_XU02G82VZ
Vat_XU02JE0VN
Erk_TZ02JEAVO
Erk_TZ02HWZLF
Vat_XU02IA6GJ
DSu_QV01DZNFQ
DSu_QV02HBFXQ
DSu_QV02JC595
DSu_QV02HOJNP
DSu_QV01BVJPT
DSu_QV01CJGMN
C_P20_2246770648
DSu_QV01B48M2
C_M09_2236659036
DSp_QV01DFRJY
DSu_QV01CT7X0
DSu_QV02IVEVO
DSu_QV01CZREE
DSu_QV02H4DHS
DSu_QV02HULSH
88
97
51
74
77
82
81
83
95
0.02

168 rpoA

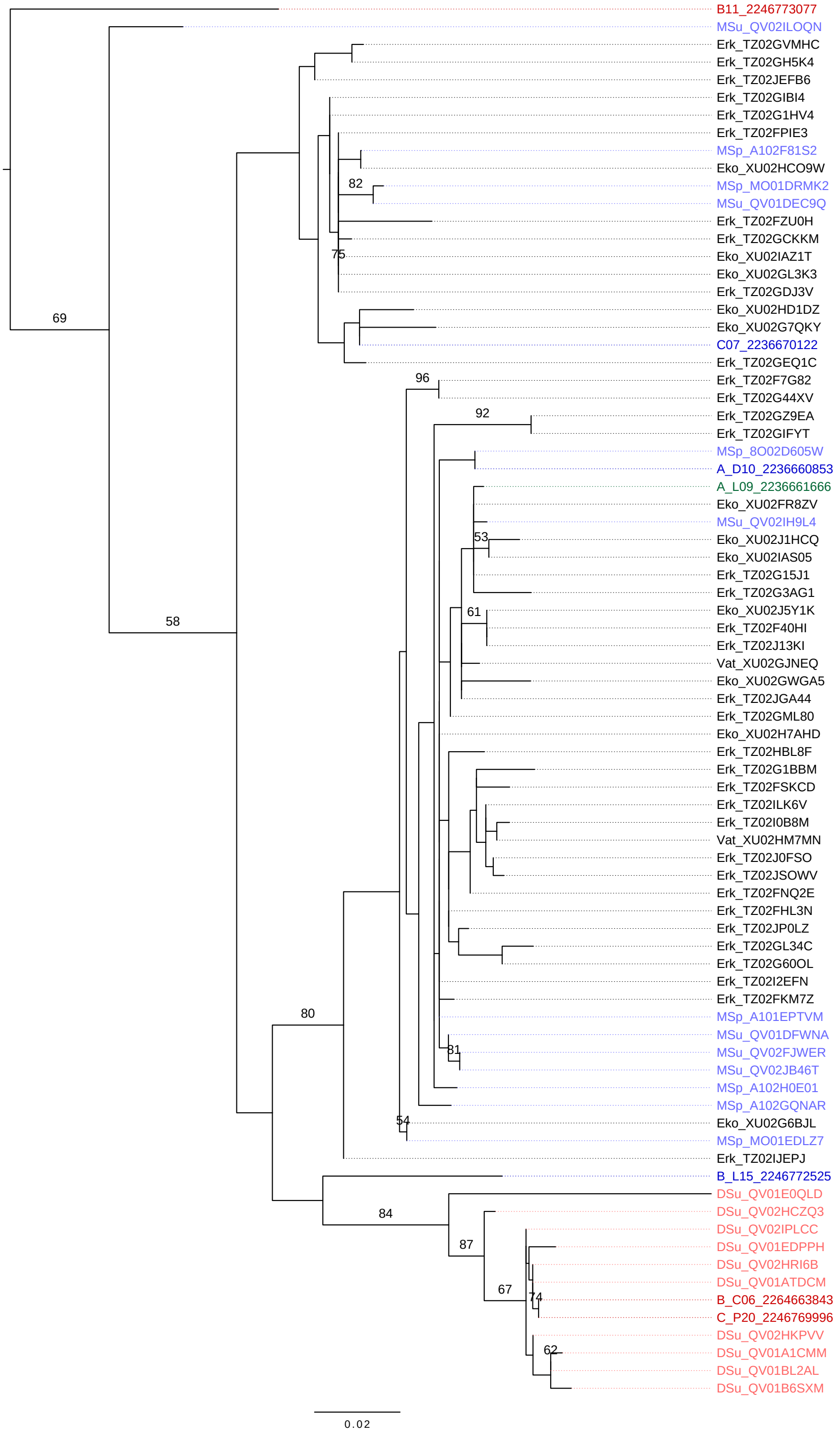
B11_2246773077
MSu_QV02ILOQN
Erk_TZ02GVMHC
Erk_TZ02GH5K4
Erk_TZ02JEFB6
Erk_TZ02GIBI4
Erk_TZ02G1HV4
Erk_TZ02FPIE3
MSp_A102F81S2
Eko_XU02HCO9W
MSp_MO01DRMK2
MSu_QV01DEC9Q
Erk_TZ02FZU0H
Erk_TZ02GCKKM
Eko_XU02IAZ1T
Eko_XU02GL3K3
Erk_TZ02GDJ3V
Eko_XU02HD1DZ
Eko_XU02G7QKY
C07_2236670122
Erk_TZ02GEQ1C
Erk_TZ02F7G82
Erk_TZ02G44XV
Erk_TZ02GZ9EA
Erk_TZ02GIFYT
MSp_8O02D605W
A_D10_2236660853
A_L09_2236661666
Eko_XU02FR8ZV
MSu_QV02IH9L4
Eko_XU02J1HCQ
Eko_XU02IAS05
Erk_TZ02G15J1
Erk_TZ02G3AG1
Eko_XU02J5Y1K
Erk_TZ02F40HI
Erk_TZ02J13KI
Vat_XU02GJNEQ
Eko_XU02GWGA5
Erk_TZ02JGA44
Erk_TZ02GML80
Eko_XU02H7AHD
Erk_TZ02HBL8F
Erk_TZ02G1BBM
Erk_TZ02FSKCD
Erk_TZ02ILK6V
Erk_TZ02I0B8M
Vat_XU02HM7MN
Erk_TZ02J0FSO
Erk_TZ02JSOWV
Erk_TZ02FNQ2E
Erk_TZ02FHL3N
Erk_TZ02JP0LZ
Erk_TZ02GL34C
Erk_TZ02G60OL
Erk_TZ02I2EFN
Erk_TZ02FKM7Z
MSp_A101EPTVM
MSu_QV01DFWNA
MSu_QV02FJWER
MSu_QV02JB46T
MSp_A102H0E01
MSp_A102GQNAR
Eko_XU02G6BJL
MSp_MO01EDLZ7
Erk_TZ02IJEPJ
B_L15_2246772525
DSu_QV01E0QLD
DSu_QV02HCZQ3
DSu_QV02IPLCC
DSu_QV01EDPPH
DSu_QV02HRI6B
DSu_QV01ATDCM
B_C06_2264663843
C_P20_2246769996
DSu_QV02HKPVV
DSu_QV01A1CMM
DSu_QV01BL2AL
DSu_QV01B6SXM
69
58
82
75
96
92
53
61
81
54
80
84
87
67
74
62
0.02

177 pepP

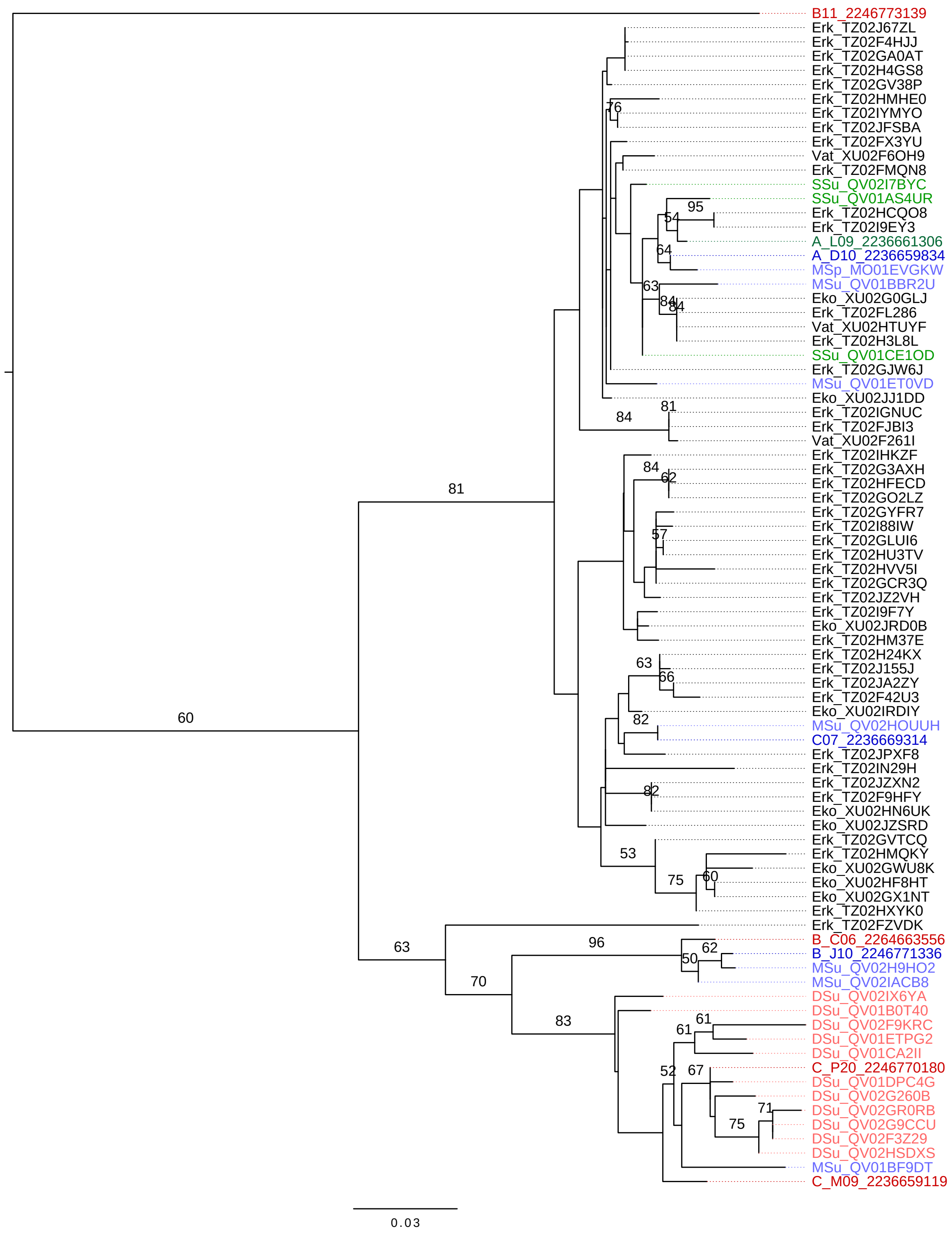
B11_2246773139
Erk_TZ02J67ZL
Erk_TZ02F4HJJ
Erk_TZ02GA0AT
Erk_TZ02H4GS8
Erk_TZ02GV38P
Erk_TZ02HMHE0
Erk_TZ02IYMYO
Erk_TZ02JFSBA
Erk_TZ02FX3YU
Vat_XU02F6OH9
Erk_TZ02FMQN8
SSu_QV02I7BYC
SSu_QV01AS4UR
Erk_TZ02HCQO8
Erk_TZ02I9EY3
A_L09_2236661306
A_D10_2236659834
MSp_MO01EVGKW
MSu_QV01BBR2U
Eko_XU02G0GLJ
Erk_TZ02FL286
Vat_XU02HTUYF
Erk_TZ02H3L8L
SSu_QV01CE1OD
Erk_TZ02GJW6J
MSu_QV01ET0VD
Eko_XU02JJ1DD
Erk_TZ02IGNUC
Erk_TZ02FJBI3
Vat_XU02F261I
Erk_TZ02IHKZF
Erk_TZ02G3AXH
Erk_TZ02HFECD
Erk_TZ02GO2LZ
Erk_TZ02GYFR7
Erk_TZ02I88IW
Erk_TZ02GLUI6
Erk_TZ02HU3TV
Erk_TZ02HVV5I
Erk_TZ02GCR3Q
Erk_TZ02JZ2VH
Erk_TZ02I9F7Y
Eko_XU02JRD0B
Erk_TZ02HM37E
Erk_TZ02H24KX
Erk_TZ02J155J
Erk_TZ02JA2ZY
Erk_TZ02F42U3
Eko_XU02IRDIY
MSu_QV02HOUUH
C07_2236669314
Erk_TZ02JPXF8
Erk_TZ02IN29H
Erk_TZ02JZXN2
Erk_TZ02F9HFY
Eko_XU02HN6UK
Eko_XU02JZSRD
Erk_TZ02GVTCQ
Erk_TZ02HMQKY
Eko_XU02GWU8K
Eko_XU02HF8HT
Eko_XU02GX1NT
Erk_TZ02HXYK0
Erk_TZ02FZVDK
B_C06_2264663556
B_J10_2246771336
MSu_QV02H9HO2
MSu_QV02IACB8
DSu_QV02IX6YA
DSu_QV01B0T40
DSu_QV02F9KRC
DSu_QV01ETPG2
DSu_QV01CA2II
C_P20_2246770180
DSu_QV01DPC4G
DSu_QV02G260B
DSu_QV02GR0RB
DSu_QV02G9CCU
DSu_QV02F3Z29
DSu_QV02HSDXS
MSu_QV01BF9DT
C_M09_2236659119
0.03

186 gcp

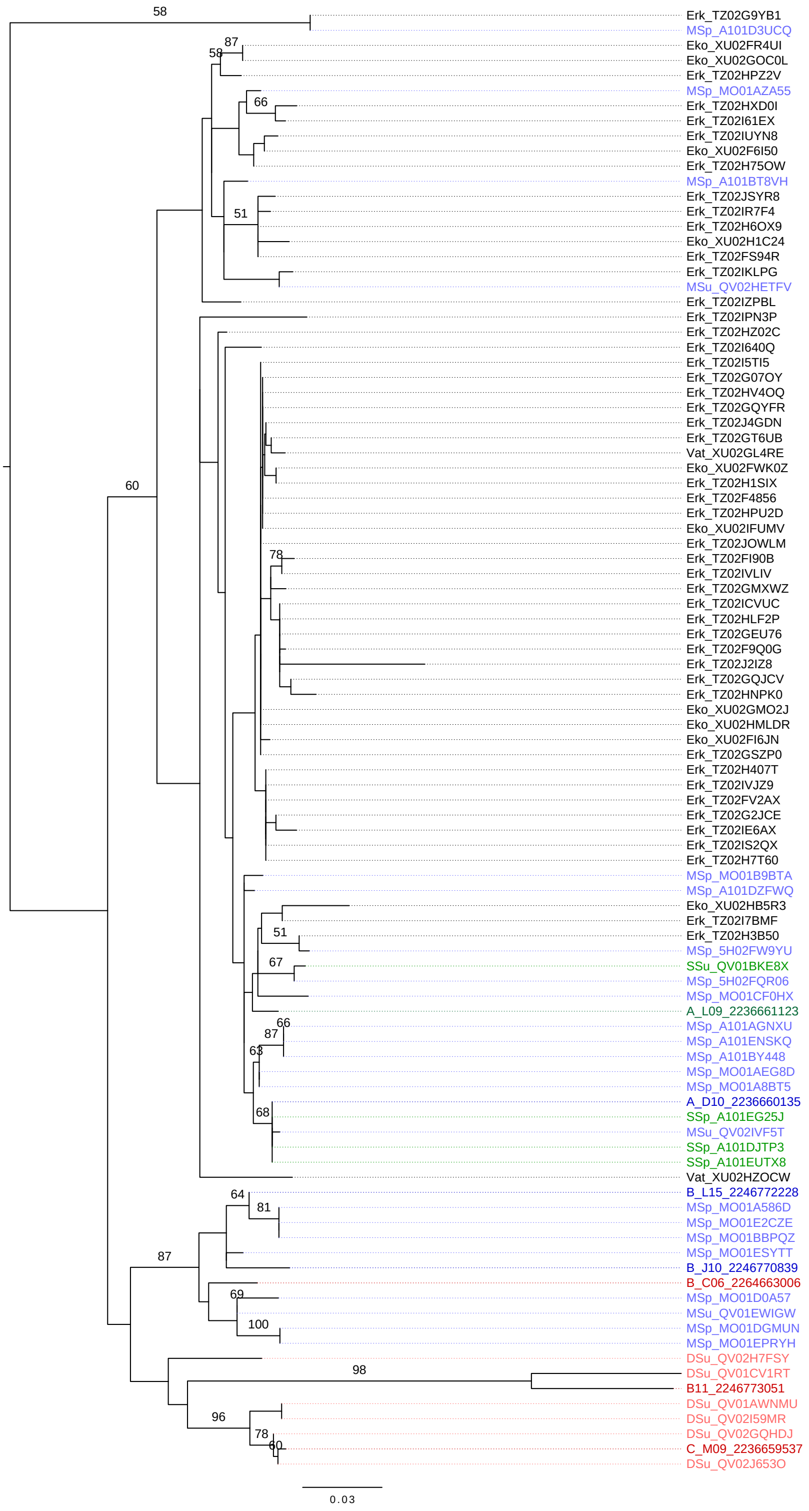
Erk_TZ02G9YB1
MSp_A101D3UCQ
Eko_XU02FR4UI
Eko_XU02GOC0L
Erk_TZ02HPZ2V
MSp_MO01AZA55
Erk_TZ02HXD0I
Erk_TZ02I61EX
Erk_TZ02IUYN8
Eko_XU02F6I50
Erk_TZ02H75OW
MSp_A101BT8VH
Erk_TZ02JSYR8
Erk_TZ02IR7F4
Erk_TZ02H6OX9
Eko_XU02H1C24
Erk_TZ02FS94R
Erk_TZ02IKLPG
MSu_QV02HETFV
Erk_TZ02IZPBL
Erk_TZ02IPN3P
Erk_TZ02HZ02C
Erk_TZ02I640Q
Erk_TZ02I5TI5
Erk_TZ02G07OY
Erk_TZ02HV4OQ
Erk_TZ02GQYFR
Erk_TZ02J4GDN
Erk_TZ02GT6UB
Vat_XU02GL4RE
Eko_XU02FWK0Z
Erk_TZ02H1SIX
Erk_TZ02F4856
Erk_TZ02HPU2D
Eko_XU02IFUMV
Erk_TZ02JOWLM
Erk_TZ02FI90B
Erk_TZ02IVLIV
Erk_TZ02GMXWZ
Erk_TZ02ICVUC
Erk_TZ02HLF2P
Erk_TZ02GEU76
Erk_TZ02F9Q0G
Erk_TZ02J2IZ8
Erk_TZ02GQJCV
Erk_TZ02HNPK0
Eko_XU02GMO2J
Eko_XU02HMLDR
Eko_XU02FI6JN
Erk_TZ02GSZP0
Erk_TZ02H407T
Erk_TZ02IVJZ9
Erk_TZ02FV2AX
Erk_TZ02G2JCE
Erk_TZ02IE6AX
Erk_TZ02IS2QX
Erk_TZ02H7T60
MSp_MO01B9BTA
MSp_A101DZFWQ
Eko_XU02HB5R3
Erk_TZ02I7BMF
Erk_TZ02H3B50
MSp_5H02FW9YU
SSu_QV01BKE8X
MSp_5H02FQR06
MSp_MO01CF0HX
A_L09_2236661123
MSp_A101AGNXU
MSp_A101ENSKQ
MSp_A101BY448
MSp_MO01AEG8D
MSp_MO01A8BT5
A_D10_2236660135
SSp_A101EG25J
MSu_QV02IVF5T
SSp_A101DJTP3
SSp_A101EUTX8
Vat_XU02HZOCW
B_L15_2246772228
MSp_MO01A586D
MSp_MO01E2CZE
MSp_MO01BBPQZ
MSp_MO01ESYTT
B_J10_2246770839
B_C06_2264663006
MSp_MO01D0A57
MSu_QV01EWIGW
MSp_MO01DGMUN
MSp_MO01EPRYH
DSu_QV02H7FSY
DSu_QV01CV1RT
B11_2246773051
DSu_QV01AWNMU
DSu_QV02I59MR
DSu_QV02GQHDJ
C_M09_2236659537
DSu_QV02J653O
0.03

205 pheS

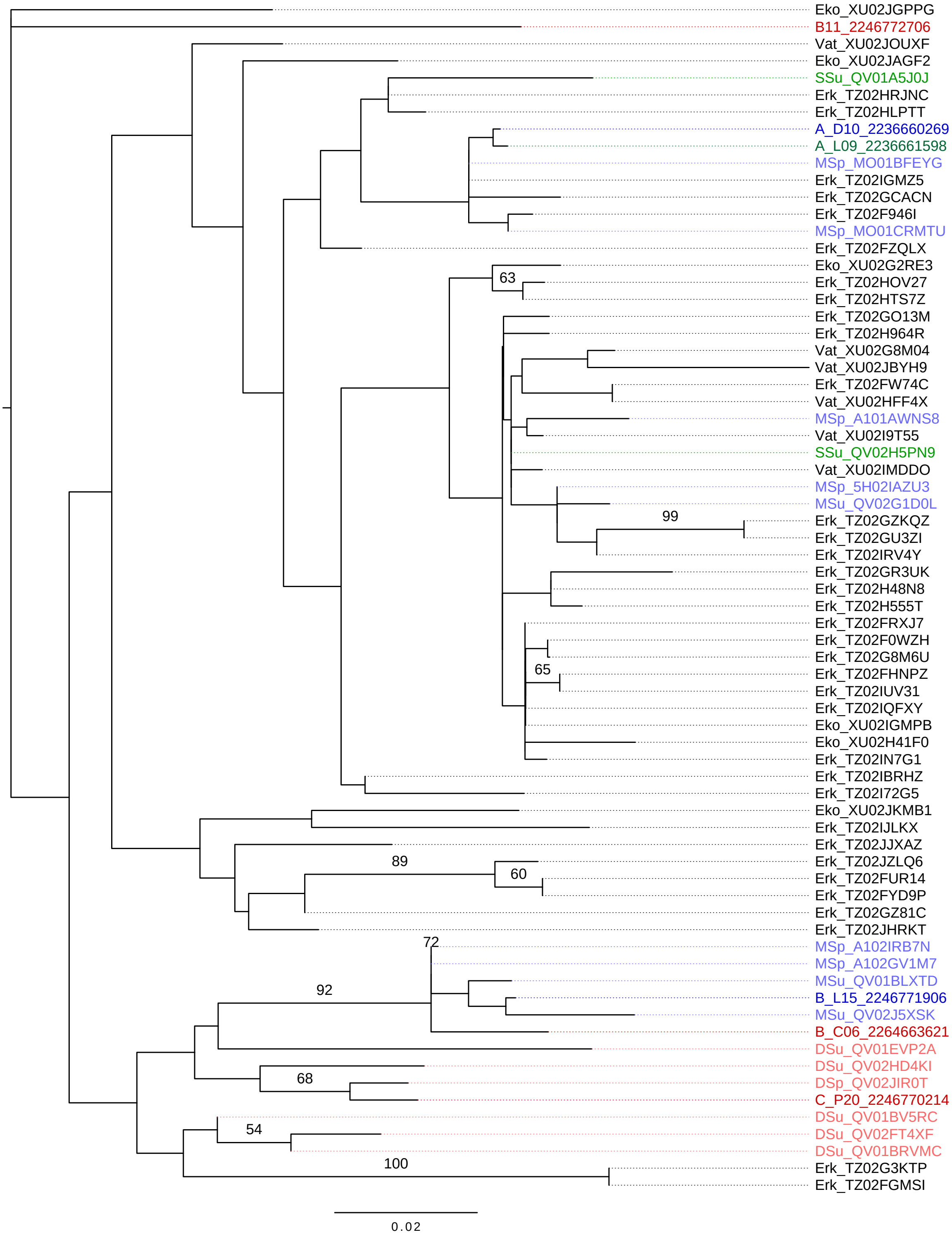

Eko_XU02JGPPG
B11_2246772706
Vat_XU02JOUXF
Eko_XU02JAGF2
SSu_QV01A5J0J
Erk_TZ02HRJNC
Erk_TZ02HLPTT
A_D10_2236660269
A_L09_2236661598
MSp_MO01BFEYG
Erk_TZ02IGMZ5
Erk_TZ02GCACN
Erk_TZ02F946I
MSp_MO01CRMTU
Erk_TZ02FZQLX
Eko_XU02G2RE3
Erk_TZ02HOV27
Erk_TZ02HTS7Z
Erk_TZ02GO13M
Erk_TZ02H964R
Vat_XU02G8M04
Vat_XU02JBYH9
Erk_TZ02FW74C
Vat_XU02HFF4X
MSp_A101AWNS8
Vat_XU02I9T55
SSu_QV02H5PN9
Vat_XU02IMDDO
MSp_5H02IAZU3
MSu_QV02G1D0L
Erk_TZ02GZKQZ
Erk_TZ02GU3ZI
Erk_TZ02IRV4Y
Erk_TZ02GR3UK
Erk_TZ02H48N8
Erk_TZ02H555T
Erk_TZ02FRXJ7
Erk_TZ02F0WZH
Erk_TZ02G8M6U
Erk_TZ02FHNPZ
Erk_TZ02IUV31
Erk_TZ02IQFXY
Eko_XU02IGMPB
Eko_XU02H41F0
Erk_TZ02IN7G1
Erk_TZ02IBRHZ
Erk_TZ02I72G5
Eko_XU02JKMB1
Erk_TZ02IJLKX
Erk_TZ02JJXAZ
Erk_TZ02JZLQ6
Erk_TZ02FUR14
Erk_TZ02FYD9P
Erk_TZ02GZ81C
Erk_TZ02JHRKT
MSp_A102IRB7N
MSp_A102GV1M7
MSu_QV01BLXTD
B_L15_2246771906
MSu_QV02J5XSK
B_C06_2264663621
DSu_QV01EVP2A
DSu_QV02HD4KI
DSp_QV02JIR0T
C_P20_2246770214
DSu_QV01BV5RC
DSu_QV02FT4XF
DSu_QV01BRVMC
Erk_TZ02G3KTP
Erk_TZ02FGMSI
63
99
65
89
60
72
92
68
54
100
0.02

206 pheT

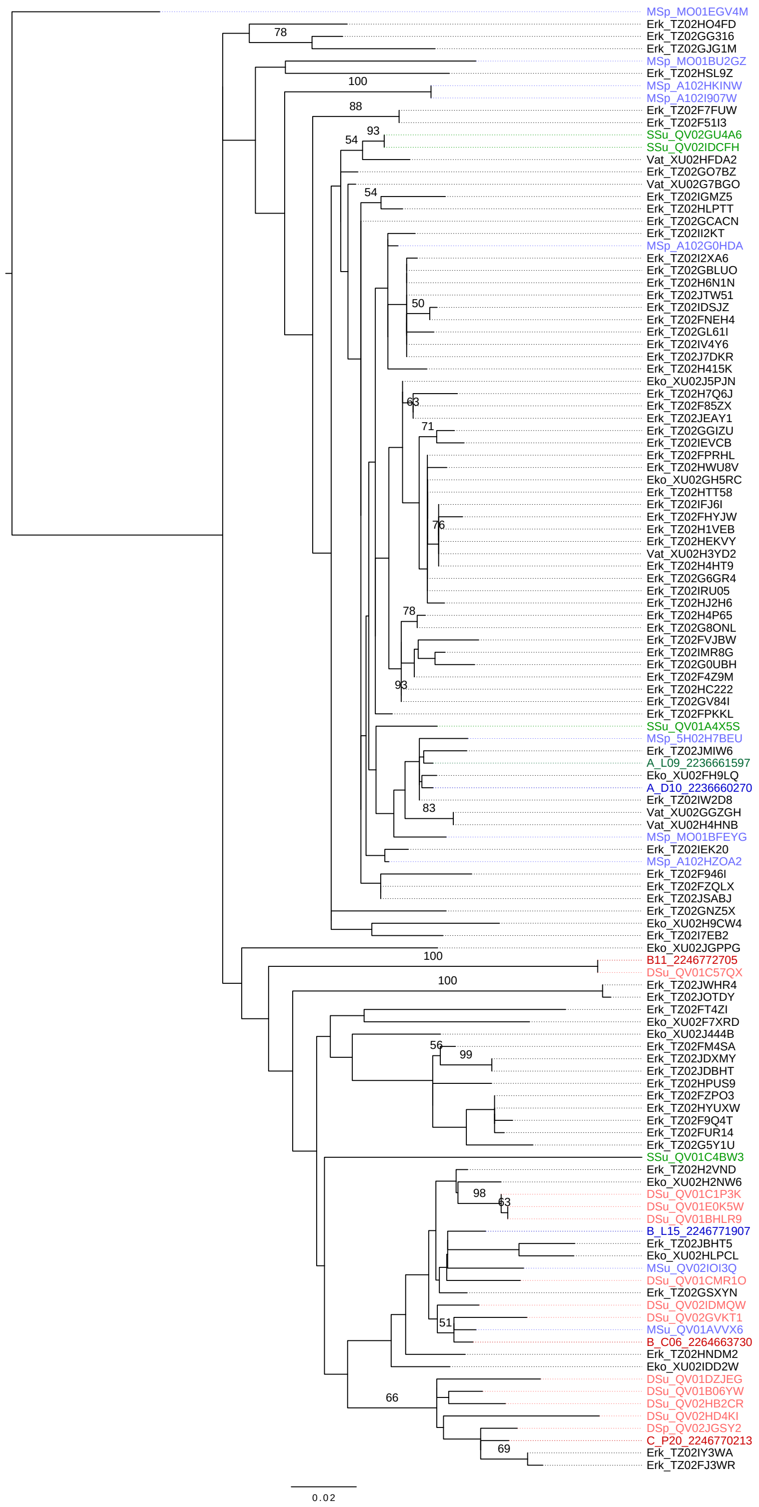
MSp_MO01EGV4M
Erk_TZ02HO4FD
Erk_TZ02GG316
Erk_TZ02GJG1M
MSp_MO01BU2GZ
Erk_TZ02HSL9Z
MSp_A102HKINW
MSp_A102I907W
Erk_TZ02F7FUW
Erk_TZ02F51I3
SSu_QV02GU4A6
SSu_QV02IDCFH
Vat_XU02HFDA2
Erk_TZ02GO7BZ
Vat_XU02G7BGO
Erk_TZ02IGMZ5
Erk_TZ02HLPTT
Erk_TZ02GCACN
Erk_TZ02II2KT
MSp_A102G0HDA
Erk_TZ02I2XA6
Erk_TZ02GBLUO
Erk_TZ02H6N1N
Erk_TZ02JTW51
Erk_TZ02IDSJZ
Erk_TZ02FNEH4
Erk_TZ02GL61I
Erk_TZ02IV4Y6
Erk_TZ02J7DKR
Erk_TZ02H415K
Eko_XU02J5PJN
Erk_TZ02H7Q6J
Erk_TZ02F85ZX
Erk_TZ02JEAY1
Erk_TZ02GGIZU
Erk_TZ02IEVCB
Erk_TZ02FPRHL
Erk_TZ02HWU8V
Eko_XU02GH5RC
Erk_TZ02HTT58
Erk_TZ02IFJ6I
Erk_TZ02FHYJW
Erk_TZ02H1VEB
Erk_TZ02HEKVY
Vat_XU02H3YD2
Erk_TZ02H4HT9
Erk_TZ02G6GR4
Erk_TZ02IRU05
Erk_TZ02HJ2H6
Erk_TZ02H4P65
Erk_TZ02G8ONL
Erk_TZ02FVJBW
Erk_TZ02IMR8G
Erk_TZ02G0UBH
Erk_TZ02F4Z9M
Erk_TZ02HC222
Erk_TZ02GV84I
Erk_TZ02FPKKL
SSu_QV01A4X5S
MSp_5H02H7BEU
Erk_TZ02JMIW6
A_L09_2236661597
Eko_XU02FH9LQ
A_D10_2236660270
Erk_TZ02IW2D8
Vat_XU02GGZGH
Vat_XU02H4HNB
MSp_MO01BFEYG
Erk_TZ02IEK20
MSp_A102HZOA2
Erk_TZ02F946I
Erk_TZ02FZQLX
Erk_TZ02JSABJ
Erk_TZ02GNZ5X
Eko_XU02H9CW4
Erk_TZ02I7EB2
Eko_XU02JGPPG
B11_2246772705
DSu_QV01C57QX
Erk_TZ02JWHR4
Erk_TZ02JOTDY
Erk_TZ02FT4ZI
Eko_XU02F7XRD
Eko_XU02J444B
Erk_TZ02FM4SA
Erk_TZ02JDXMY
Erk_TZ02JDBHT
Erk_TZ02HPUS9
Erk_TZ02FZPO3
Erk_TZ02HYUXW
Erk_TZ02F9Q4T
Erk_TZ02FUR14
Erk_TZ02G5Y1U
SSu_QV01C4BW3
Erk_TZ02H2VND
Eko_XU02H2NW6
DSu_QV01C1P3K
DSu_QV01E0K5W
DSu_QV01BHLR9
B_L15_2246771907
Erk_TZ02JBHT5
Eko_XU02HLPCL
MSu_QV02IOI3Q
DSu_QV01CMR1O
Erk_TZ02GSXYN
DSu_QV02IDMQW
DSu_QV02GVKT1
MSu_QV01AVVX6
B_C06_2264663730
Erk_TZ02HNDM2
Eko_XU02IDD2W
DSu_QV01DZJEG
DSu_QV01B06YW
DSu_QV02HB2CR
DSu_QV02HD4KI
DSp_QV02JGSY2
C_P20_2246770213
Erk_TZ02IY3WA
Erk_TZ02FJ3WR
78
100
88
93
54
54
50
63
71
76
78
93
83
100
100
56
99
98
63
51
66
69
0.02

227 thrS

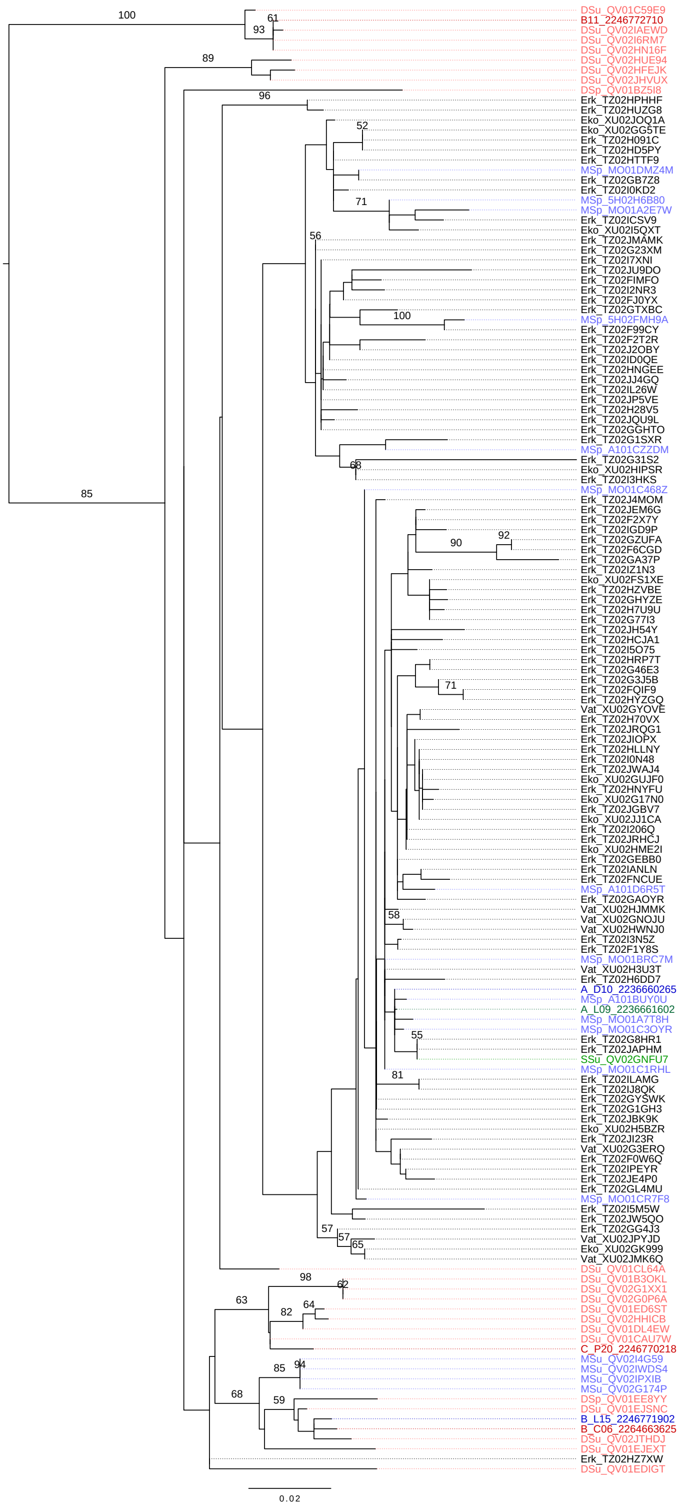

DSu_QV01C59E9
B11_2246772710
DSu_QV02IAEWD
DSu_QV02I6RM7
DSu_QV02HN16F
DSu_QV02HUE94
DSu_QV02HFEJK
DSu_QV02JHVUX
DSp_QV01BZ5I8
Erk_TZ02HPHHF
Erk_TZ02HUZG8
Eko_XU02JOQ1A
Eko_XU02GG5TE
Erk_TZ02H091C
Erk_TZ02HD5PY
Erk_TZ02HTTF9
MSp_MO01DMZ4M
Erk_TZ02GB7Z8
Erk_TZ02I0KD2
MSp_5H02H6B80
MSp_MO01A2E7W
Erk_TZ02ICSV9
Eko_XU02I5QXT
Erk_TZ02JMAMK
Erk_TZ02G23XM
Erk_TZ02I7XNI
Erk_TZ02JU9DO
Erk_TZ02FIMFO
Erk_TZ02I2NR3
Erk_TZ02FJ0YX
Erk_TZ02GTXBC
MSp_5H02FMH9A
Erk_TZ02F99CY
Erk_TZ02F2T2R
Erk_TZ02J2OBY
Erk_TZ02ID0QE
Erk_TZ02HNGEE
Erk_TZ02JJ4GQ
Erk_TZ02IL26W
Erk_TZ02JP5VE
Erk_TZ02H28V5
Erk_TZ02JQU9L
Erk_TZ02GGHTO
Erk_TZ02G1SXR
MSp_A101CZZDM
Erk_TZ02G31S2
Eko_XU02HIPSR
Erk_TZ02I3HKS
MSp_MO01C468Z
Erk_TZ02J4MOM
Erk_TZ02JEM6G
Erk_TZ02F2X7Y
Erk_TZ02IGD9P
Erk_TZ02GZUFA
Erk_TZ02F6CGD
Erk_TZ02GA37P
Erk_TZ02IZ1N3
Eko_XU02FS1XE
Erk_TZ02HZVBE
Erk_TZ02GHYZE
Erk_TZ02H7U9U
Erk_TZ02G77I3
Erk_TZ02JH54Y
Erk_TZ02HCJA1
Erk_TZ02I5O75
Erk_TZ02HRP7T
Erk_TZ02G46E3
Erk_TZ02G3J5B
Erk_TZ02FQIF9
Erk_TZ02HYZGQ
Vat_XU02GYOVE
Erk_TZ02H70VX
Erk_TZ02JRQG1
Erk_TZ02JIOPX
Erk_TZ02HLLNY
Erk_TZ02I0N48
Erk_TZ02JWAJ4
Eko_XU02GUJF0
Erk_TZ02HNYFU
Eko_XU02G17N0
Erk_TZ02JGBV7
Eko_XU02JJ1CA
Erk_TZ02I206Q
Erk_TZ02JRHCJ
Eko_XU02HME2I
Erk_TZ02GEBB0
Erk_TZ02IANLN
Erk_TZ02FNCUE
MSp_A101D6R5T
Erk_TZ02GAOYR
Vat_XU02HJMMK
Vat_XU02GNOJU
Vat_XU02HWNJ0
Erk_TZ02I3N5Z
Erk_TZ02F1Y8S
MSp_MO01BRC7M
Vat_XU02H3U3T
Erk_TZ02H6DD7
A_D10_2236660265
MSp_A101BUY0U
A_L09_2236661602
MSp_MO01A7T8H
MSp_MO01C3OYR
Erk_TZ02G8HR1
Erk_TZ02JAPHM
SSu_QV02GNFU7
MSp_MO01C1RHL
Erk_TZ02ILAMG
Erk_TZ02IJ8QK
Erk_TZ02GYSWK
Erk_TZ02G1GH3
Erk_TZ02JBK9K
Eko_XU02H5BZR
Erk_TZ02JI23R
Vat_XU02G3ERQ
Erk_TZ02F0W6Q
Erk_TZ02IPEYR
Erk_TZ02JE4P0
Erk_TZ02GL4MU
MSp_MO01CR7F8
Erk_TZ02I5M5W
Erk_TZ02JW5QO
Erk_TZ02GG4J3
Vat_XU02JPYJD
Eko_XU02GK999
Vat_XU02JMK6Q
DSu_QV01CL64A
DSu_QV01B3OKL
DSu_QV02G1XX1
DSu_QV02G0P6A
DSu_QV01ED6ST
DSu_QV02HHICB
DSu_QV01DL4EW
DSu_QV01CAU7W
C_P20_2246770218
MSu_QV02I4G59
MSu_QV02IWDS4
MSu_QV02IPXIB
MSu_QV02G174P
DSp_QV01EE8YY
DSu_QV01EJSNC
B_L15_2246771902
B_C06_2264663625
DSu_QV02JTHDJ
DSu_QV01EJEXT
Erk_TZ02HZ7XW
DSu_QV01EDIGT
100
61
93
89
96
52
71
56
100
68
85
90
92
71
58
55
81
57
57
65
98
62
63
64
82
85
94
68
59
0.02

247 hemE

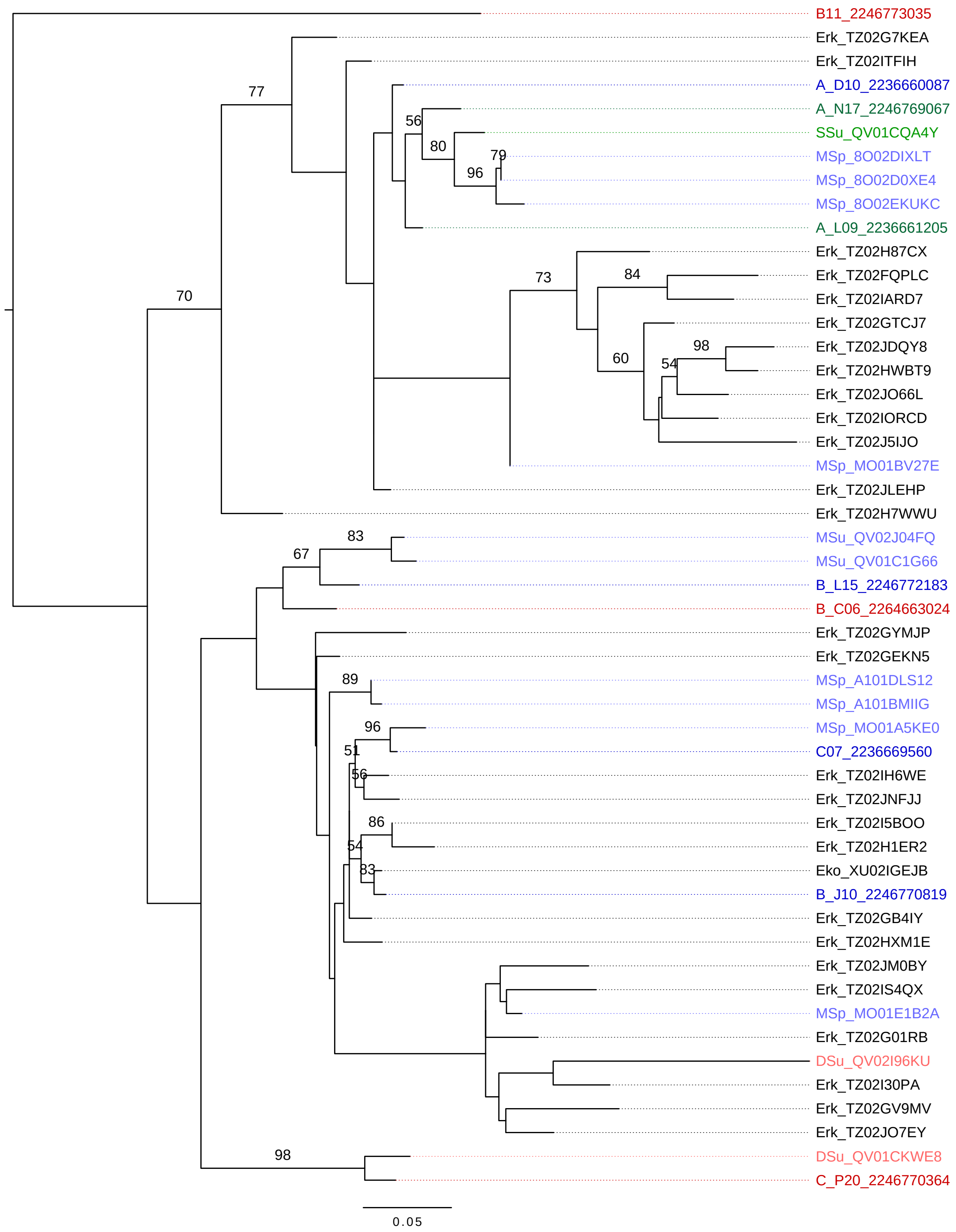

B11_2246773035
Erk_TZ02G7KEA
Erk_TZ02ITFIH
A_D10_2236660087
A_N17_2246769067
SSu_QV01CQA4Y
MSp_8O02DIXLT
MSp_8O02D0XE4
MSp_8O02EKUKC
A_L09_2236661205
Erk_TZ02H87CX
Erk_TZ02FQPLC
Erk_TZ02IARD7
Erk_TZ02GTCJ7
Erk_TZ02JDQY8
Erk_TZ02HWBT9
Erk_TZ02JO66L
Erk_TZ02IORCD
Erk_TZ02J5IJO
MSp_MO01BV27E
Erk_TZ02JLEHP
Erk_TZ02H7WWU
MSu_QV02J04FQ
MSu_QV01C1G66
B_L15_2246772183
B_C06_2264663024
Erk_TZ02GYMJP
Erk_TZ02GEKN5
MSp_A101DLS12
MSp_A101BMIIG
MSp_MO01A5KE0
C07_2236669560
Erk_TZ02IH6WE
Erk_TZ02JNFJJ
Erk_TZ02I5BOO
Erk_TZ02H1ER2
Eko_XU02IGEJB
B_J10_2246770819
Erk_TZ02GB4IY
Erk_TZ02HXM1E
Erk_TZ02JM0BY
Erk_TZ02IS4QX
MSp_MO01E1B2A
Erk_TZ02G01RB
DSu_QV02I96KU
Erk_TZ02I30PA
Erk_TZ02GV9MV
Erk_TZ02JO7EY
DSu_QV01CKWE8
C_P20_2246770364
77
56
80
79
96
70
73
84
98
60
54
83
67
89
96
51
56
86
54
83
98
0.05

257 ubiB

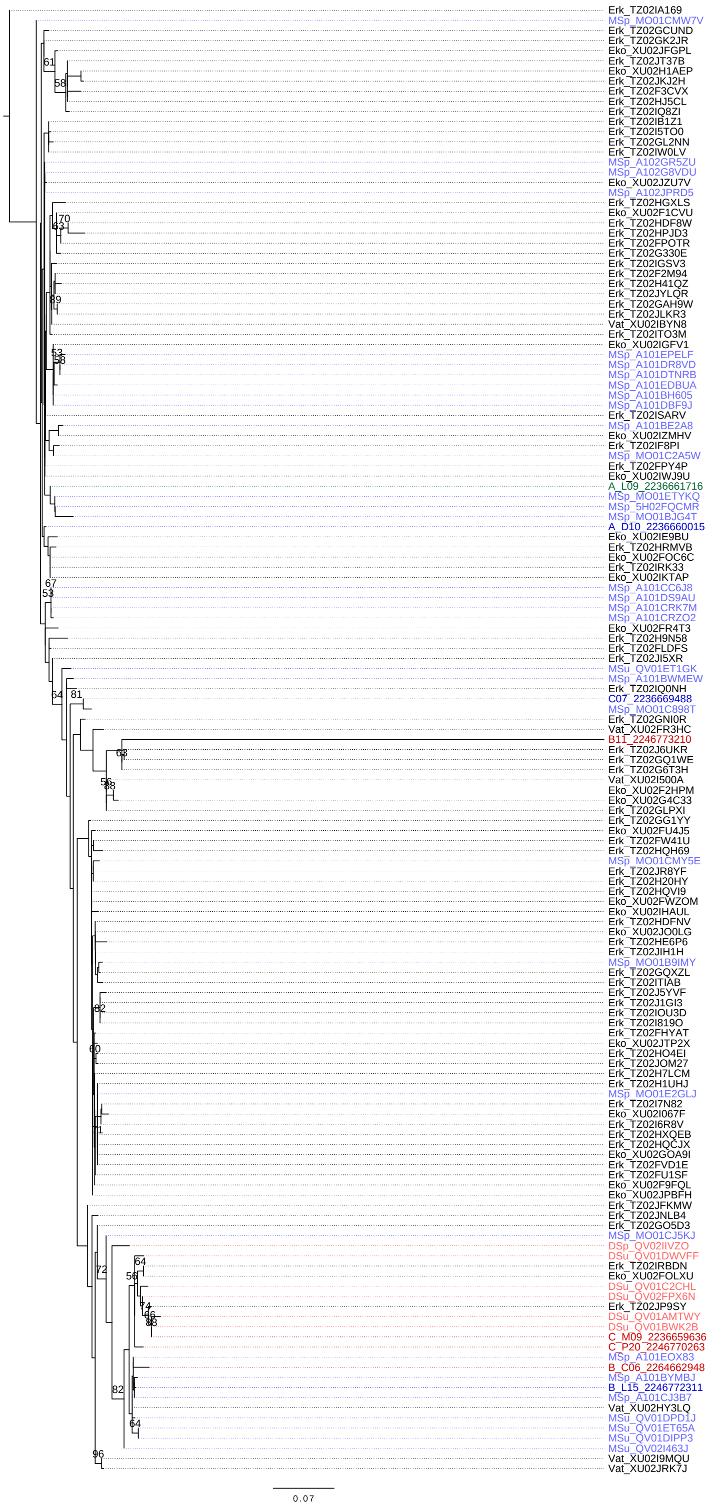

Erk_TZ02IA169
MSp_MO01CMW7V
Erk_TZ02GCUND
Erk_TZ02GK2JR
Eko_XU02JFGPL
Erk_TZ02JT37B
Eko_XU02H1AEP
Erk_TZ02JKJ2H
Erk_TZ02F3CVX
Erk_TZ02HJ5CL
Erk_TZ02IQ8ZI
Erk_TZ02IB1Z1
Erk_TZ02I5TO0
Erk_TZ02GL2NN
Erk_TZ02IW0LV
MSp_A102GR5ZU
MSp_A102G8VDU
Eko_XU02JZU7V
MSp_A102JPRD5
Erk_TZ02HGXLS
Eko_XU02F1CVU
Erk_TZ02HDF8W
Erk_TZ02HPJD3
Erk_TZ02FPOTR
Erk_TZ02G330E
Erk_TZ02IGSV3
Erk_TZ02F2M94
Erk_TZ02H41QZ
Erk_TZ02JYLQR
Erk_TZ02GAH9W
Erk_TZ02JLKR3
Vat_XU02IBYN8
Erk_TZ02ITO3M
Eko_XU02IGFV1
MSp_A101EPELF
MSp_A101DR8VD
MSp_A101DTNRB
MSp_A101EDBUA
MSp_A101BH605
MSp_A101DBF9J
Erk_TZ02ISARV
MSp_A101BE2A8
Eko_XU02IZMHV
Erk_TZ02IF8PI
MSp_MO01C2A5W
Erk_TZ02FPY4P
Eko_XU02IWJ9U
A_L09_2236661716
MSp_MO01ETYKQ
MSp_5H02FQCMR
MSp_MO01BJG4T
A_D10_2236660015
Eko_XU02IE9BU
Erk_TZ02HRMVB
Eko_XU02FOC6C
Erk_TZ02IRK33
Eko_XU02IKTAP
MSp_A101CC6J8
MSp_A101DS9AU
MSp_A101CRK7M
MSp_A101CRZO2
Eko_XU02FR4T3
Erk_TZ02H9N58
Erk_TZ02FLDFS
Erk_TZ02JI5XR
MSu_QV01ET1GK
MSp_A101BWMEW
Erk_TZ02IQ0NH
C07_2236669488
MSp_MO01C898T
Erk_TZ02GNI0R
Vat_XU02FR3HC
B11_2246773210
Erk_TZ02J6UKR
Erk_TZ02GQ1WE
Erk_TZ02G6T3H
Vat_XU02I500A
Eko_XU02F2HPM
Eko_XU02G4C33
Erk_TZ02GLPXI
Erk_TZ02GG1YY
Eko_XU02FU4J5
Erk_TZ02FW41U
Erk_TZ02HQH69
MSp_MO01CMY5E
Erk_TZ02JR8YF
Erk_TZ02H20HY
Erk_TZ02HQVI9
Eko_XU02FWZOM
Eko_XU02IHAUL
Erk_TZ02HDFNV
Eko_XU02JO0LG
Erk_TZ02HE6P6
Erk_TZ02JIH1H
MSp_MO01B9IMY
Erk_TZ02GQXZL
Erk_TZ02ITIAB
Erk_TZ02J5YVF
Erk_TZ02J1GI3
Erk_TZ02IOU3D
Erk_TZ02I819O
Erk_TZ02FHYAT
Eko_XU02JTP2X
Erk_TZ02HO4EI
Erk_TZ02JOM27
Erk_TZ02H7LCM
Erk_TZ02H1UHJ
MSp_MO01E2GLJ
Erk_TZ02I7N82
Eko_XU02I067F
Erk_TZ02I6R8V
Erk_TZ02HXQEB
Erk_TZ02HQCJX
Eko_XU02GOA9I
Erk_TZ02FVD1E
Erk_TZ02FU1SF
Eko_XU02F9FQL
Eko_XU02JPBFH
Erk_TZ02JFKMW
Erk_TZ02JNLB4
Erk_TZ02GO5D3
MSp_MO01CJ5KJ
DSp_QV02IIVZO
DSu_QV01DWVFF
Erk_TZ02IRBDN
Eko_XU02FOLXU
DSu_QV01C2CHL
DSu_QV02FPX6N
Erk_TZ02JP9SY
DSu_QV01AMTWY
DSu_QV01BWK2B
C_M09_2236659636
C_P20_2246770263
MSp_A101EOX83
B_C06_2264662948
MSp_A101BYMBJ
B_L15_2246772311
MSp_A101CJ3B7
Vat_XU02HY3LQ
MSu_QV01DPD1J
MSu_QV01ET65A
MSu_QV01DIPP3
MSu_QV02I463J
Vat_XU02I9MQU
Vat_XU02JRK7J
0.07

294 hemH

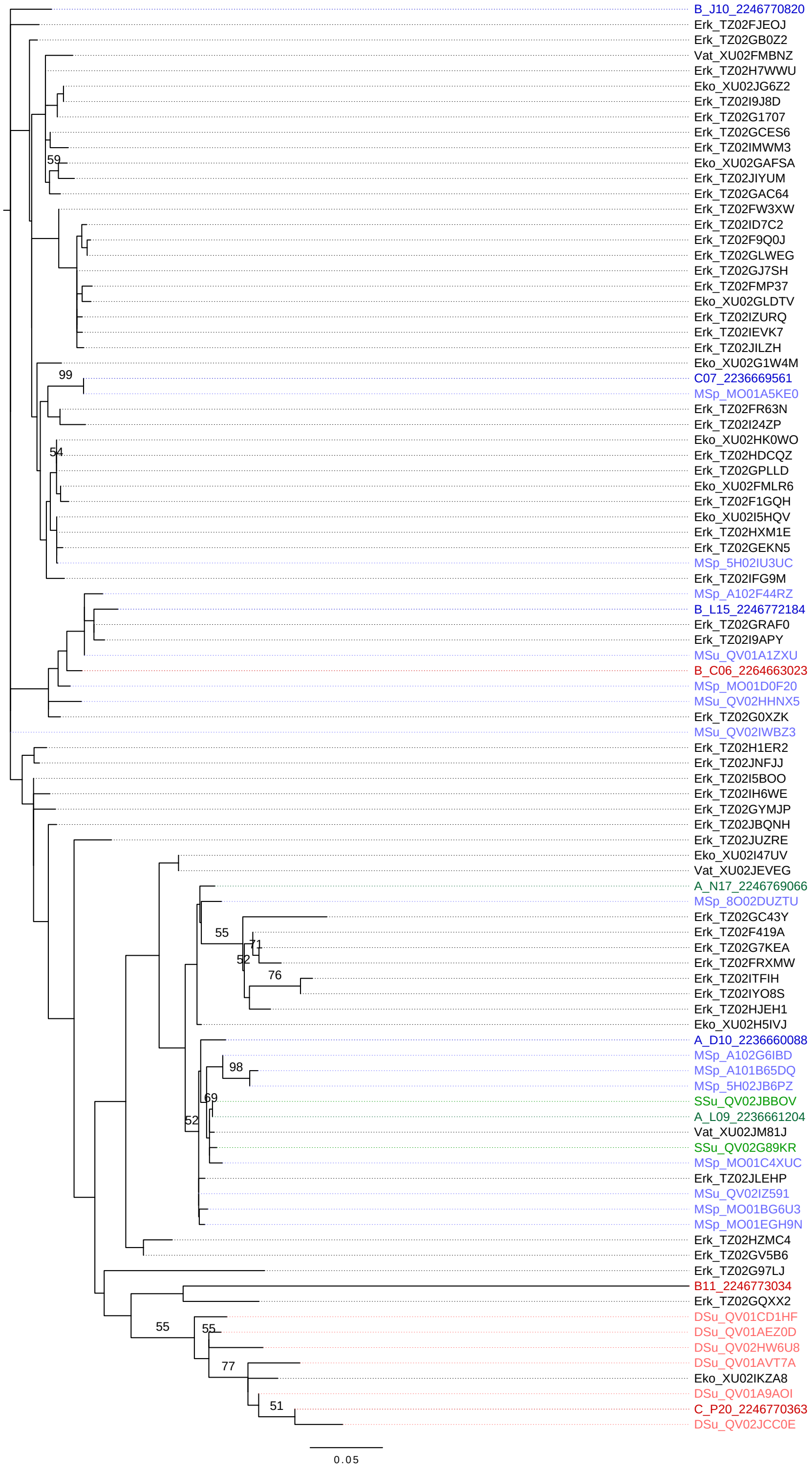
B_J10_2246770820
Erk_TZ02FJEOJ
Erk_TZ02GB0Z2
Vat_XU02FMBNZ
Erk_TZ02H7WWU
Eko_XU02JG6Z2
Erk_TZ02I9J8D
Erk_TZ02G1707
Erk_TZ02GCES6
Erk_TZ02IMWM3
Eko_XU02GAFSA
Erk_TZ02JIYUM
Erk_TZ02GAC64
Erk_TZ02FW3XW
Erk_TZ02ID7C2
Erk_TZ02F9Q0J
Erk_TZ02GLWEG
Erk_TZ02GJ7SH
Erk_TZ02FMP37
Eko_XU02GLDTV
Erk_TZ02IZURQ
Erk_TZ02IEVK7
Erk_TZ02JILZH
Eko_XU02G1W4M
C07_2236669561
MSp_MO01A5KE0
Erk_TZ02FR63N
Erk_TZ02I24ZP
Eko_XU02HK0WO
Erk_TZ02HDCQZ
Erk_TZ02GPLLD
Eko_XU02FMLR6
Erk_TZ02F1GQH
Eko_XU02I5HQV
Erk_TZ02HXM1E
Erk_TZ02GEKN5
MSp_5H02IU3UC
Erk_TZ02IFG9M
MSp_A102F44RZ
B_L15_2246772184
Erk_TZ02GRAF0
Erk_TZ02I9APY
MSu_QV01A1ZXU
B_C06_2264663023
MSp_MO01D0F20
MSu_QV02HHNX5
Erk_TZ02G0XZK
MSu_QV02IWBZ3
Erk_TZ02H1ER2
Erk_TZ02JNFJJ
Erk_TZ02I5BOO
Erk_TZ02IH6WE
Erk_TZ02GYMJP
Erk_TZ02JBQNH
Erk_TZ02JUZRE
Eko_XU02I47UV
Vat_XU02JEVEG
A_N17_2246769066
MSp_8O02DUZTU
Erk_TZ02GC43Y
Erk_TZ02F419A
Erk_TZ02G7KEA
Erk_TZ02FRXMW
Erk_TZ02ITFIH
Erk_TZ02IYO8S
Erk_TZ02HJEH1
Eko_XU02H5IVJ
A_D10_2236660088
MSp_A102G6IBD
MSp_A101B65DQ
MSp_5H02JB6PZ
SSu_QV02JBBOV
A_L09_2236661204
Vat_XU02JM81J
SSu_QV02G89KR
MSp_MO01C4XUC
Erk_TZ02JLEHP
MSu_QV02IZ591
MSp_MO01BG6U3
MSp_MO01EGH9N
Erk_TZ02HZMC4
Erk_TZ02GV5B6
Erk_TZ02G97LJ
B11_2246773034
Erk_TZ02GQXX2
DSu_QV01CD1HF
DSu_QV01AEZ0D
DSu_QV02HW6U8
DSu_QV01AVT7A
Eko_XU02IKZA8
DSu_QV01A9AOI
C_P20_2246770363
DSu_QV02JCC0E
59
99
54
55
71
52
76
98
69
52
55
55
77
51
0.05

398 murC

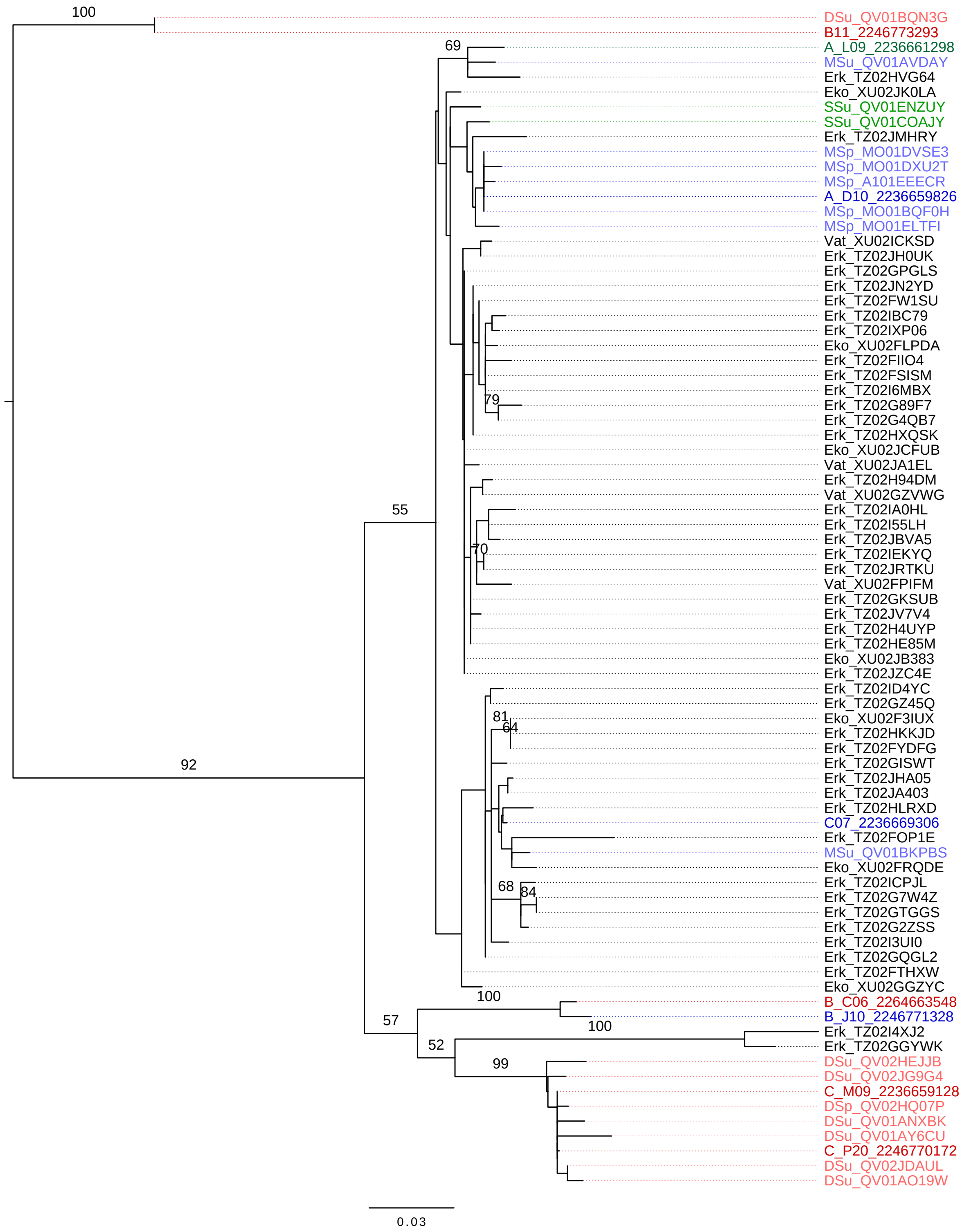
DSu_QV01BQN3G
B11_2246773293
A_L09_2236661298
MSu_QV01AVDAY
Erk_TZ02HVG64
Eko_XU02JK0LA
SSu_QV01ENZUY
SSu_QV01COAJY
Erk_TZ02JMHRY
MSp_MO01DVSE3
MSp_MO01DXU2T
MSp_A101EEECR
A_D10_2236659826
MSp_MO01BQF0H
MSp_MO01ELTFI
Vat_XU02ICKSD
Erk_TZ02JH0UK
Erk_TZ02GPGLS
Erk_TZ02JN2YD
Erk_TZ02FW1SU
Erk_TZ02IBC79
Erk_TZ02IXP06
Eko_XU02FLPDA
Erk_TZ02FIIO4
Erk_TZ02FSISM
Erk_TZ02I6MBX
Erk_TZ02G89F7
Erk_TZ02G4QB7
Erk_TZ02HXQSK
Eko_XU02JCFUB
Vat_XU02JA1EL
Erk_TZ02H94DM
Vat_XU02GZVWG
Erk_TZ02IA0HL
Erk_TZ02I55LH
Erk_TZ02JBVA5
Erk_TZ02IEKYQ
Erk_TZ02JRTKU
Vat_XU02FPIFM
Erk_TZ02GKSUB
Erk_TZ02JV7V4
Erk_TZ02H4UYP
Erk_TZ02HE85M
Eko_XU02JB383
Erk_TZ02JZC4E
Erk_TZ02ID4YC
Erk_TZ02GZ45Q
Eko_XU02F3IUX
Erk_TZ02HKKJD
Erk_TZ02FYDFG
Erk_TZ02GISWT
Erk_TZ02JHA05
Erk_TZ02JA403
Erk_TZ02HLRXD
C07_2236669306
Erk_TZ02FOP1E
MSu_QV01BKPBS
Eko_XU02FRQDE
Erk_TZ02ICPJL
Erk_TZ02G7W4Z
Erk_TZ02GTGGS
Erk_TZ02G2ZSS
Erk_TZ02I3UI0
Erk_TZ02GQGL2
Erk_TZ02FTHXW
Eko_XU02GGZYC
B_C06_2264663548
B_J10_2246771328
Erk_TZ02I4XJ2
Erk_TZ02GGYWK
DSu_QV02HEJJB
DSu_QV02JG9G4
C_M09_2236659128
DSp_QV02HQ07P
DSu_QV01ANXBK
DSu_QV01AY6CU
C_P20_2246770172
DSu_QV02JDAUL
DSu_QV01AO19W
100
69
55
92
79
70
81
64
68
84
100
100
57
52
99
0.03

399 murG

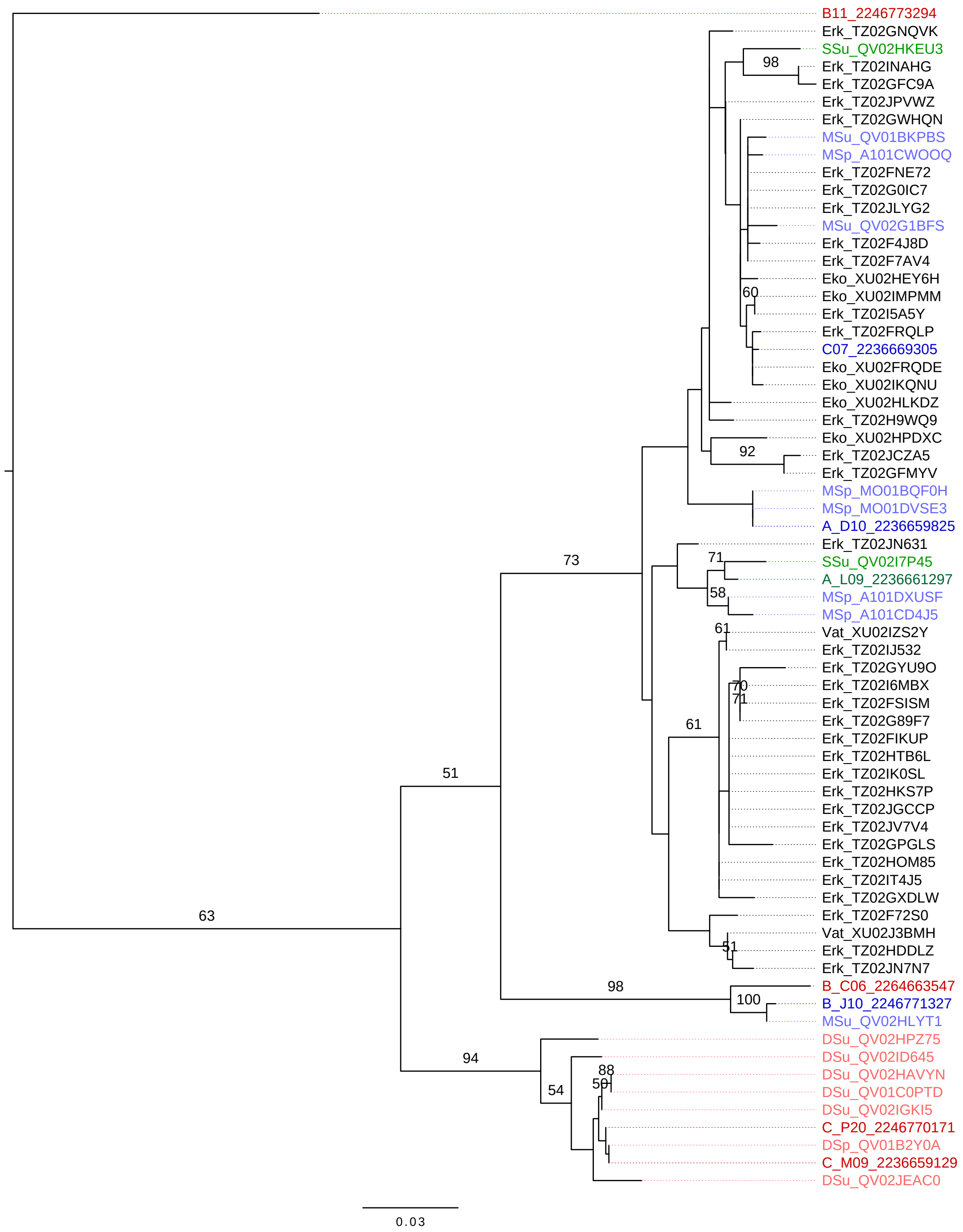
B11_2246773294
Erk_TZ02GNQVK
SSu_QV02HKEU3
Erk_TZ02INAHG
Erk_TZ02GFC9A
Erk_TZ02JPVWZ
Erk_TZ02GWHQN
MSu_QV01BKPBS
MSp_A101CWOOQ
Erk_TZ02FNE72
Erk_TZ02G0IC7
Erk_TZ02JLYG2
MSu_QV02G1BFS
Erk_TZ02F4J8D
Erk_TZ02F7AV4
Eko_XU02HEY6H
Eko_XU02IMPMM
Erk_TZ02I5A5Y
Erk_TZ02FRQLP
C07_2236669305
Eko_XU02FRQDE
Eko_XU02IKQNU
Eko_XU02HLKDZ
Erk_TZ02H9WQ9
Eko_XU02HPDXC
Erk_TZ02JCZA5
Erk_TZ02GFMYV
MSp_MO01BQF0H
MSp_MO01DVSE3
A_D10_2236659825
Erk_TZ02JN631
SSu_QV02I7P45
A_L09_2236661297
MSp_A101DXUSF
MSp_A101CD4J5
Vat_XU02IZS2Y
Erk_TZ02IJ532
Erk_TZ02GYU9O
Erk_TZ02I6MBX
Erk_TZ02FSISM
Erk_TZ02G89F7
Erk_TZ02FIKUP
Erk_TZ02HTB6L
Erk_TZ02IK0SL
Erk_TZ02HKS7P
Erk_TZ02JGCCP
Erk_TZ02JV7V4
Erk_TZ02GPGLS
Erk_TZ02HOM85
Erk_TZ02IT4J5
Erk_TZ02GXDLW
Erk_TZ02F72S0
Vat_XU02J3BMH
Erk_TZ02HDDLZ
Erk_TZ02JN7N7
B_C06_2264663547
B_J10_2246771327
MSu_QV02HLYT1
DSu_QV02HPZ75
DSu_QV02ID645
DSu_QV02HAVYN
DSu_QV01C0PTD
DSu_QV02IGKI5
C_P20_2246770171
DSp_QV01B2Y0A
C_M09_2236659129
DSu_QV02JEAC0
98
60
92
73
71
58
61
70
71
61
51
63
51
98
100
94
88
50
54
0.03

409 coaBC

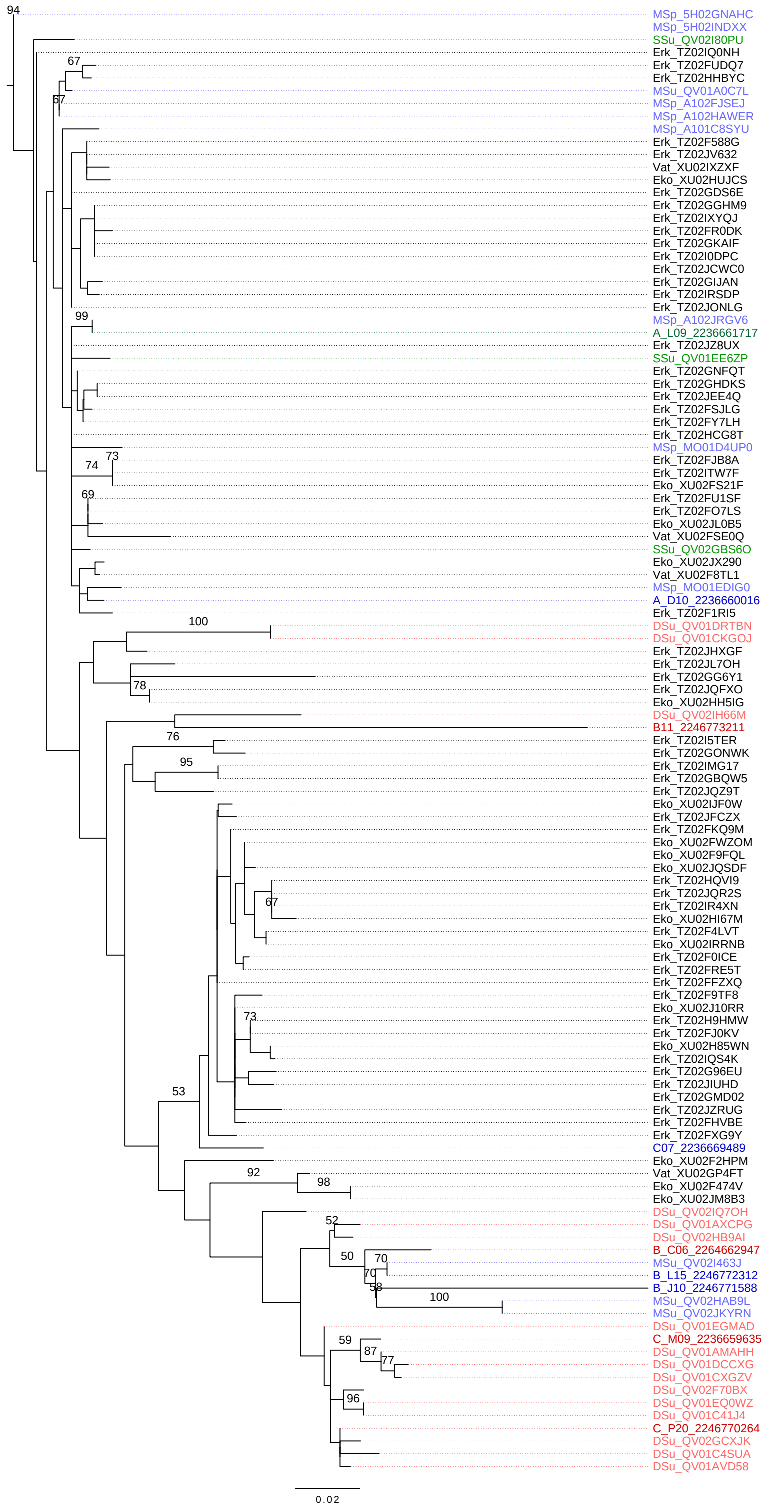

MSp_5H02GNAHC
MSp_5H02INDXX
SSu_QV02I80PU
Erk_TZ02IQ0NH
Erk_TZ02FUDQ7
Erk_TZ02HHBYC
MSu_QV01A0C7L
MSp_A102FJSEJ
MSp_A102HAWER
MSp_A101C8SYU
Erk_TZ02F588G
Erk_TZ02JV632
Vat_XU02IXZXF
Eko_XU02HUJCS
Erk_TZ02GDS6E
Erk_TZ02GGHM9
Erk_TZ02IXYQJ
Erk_TZ02FR0DK
Erk_TZ02GKAIF
Erk_TZ02I0DPC
Erk_TZ02JCWC0
Erk_TZ02GIJAN
Erk_TZ02IRSDP
Erk_TZ02JONLG
MSp_A102JRGV6
A_L09_2236661717
Erk_TZ02JZ8UX
SSu_QV01EE6ZP
Erk_TZ02GNFQT
Erk_TZ02GHDKS
Erk_TZ02JEE4Q
Erk_TZ02FSJLG
Erk_TZ02FY7LH
Erk_TZ02HCG8T
MSp_MO01D4UP0
Erk_TZ02FJB8A
Erk_TZ02ITW7F
Eko_XU02FS21F
Erk_TZ02FU1SF
Erk_TZ02FO7LS
Eko_XU02JL0B5
Vat_XU02FSE0Q
SSu_QV02GBS6O
Eko_XU02JX290
Vat_XU02F8TL1
MSp_MO01EDIG0
A_D10_2236660016
Erk_TZ02F1RI5
DSu_QV01DRTBN
DSu_QV01CKGOJ
Erk_TZ02JHXGF
Erk_TZ02JL7OH
Erk_TZ02GG6Y1
Erk_TZ02JQFXO
Eko_XU02HH5IG
DSu_QV02IH66M
B11_2246773211
Erk_TZ02I5TER
Erk_TZ02GONWK
Erk_TZ02IMG17
Erk_TZ02GBQW5
Erk_TZ02JQZ9T
Eko_XU02IJF0W
Erk_TZ02JFCZX
Erk_TZ02FKQ9M
Eko_XU02FWZOM
Eko_XU02F9FQL
Eko_XU02JQSDF
Erk_TZ02HQVI9
Erk_TZ02JQR2S
Erk_TZ02IR4XN
Eko_XU02HI67M
Erk_TZ02F4LVT
Eko_XU02IRRNB
Erk_TZ02F0ICE
Erk_TZ02FRE5T
Erk_TZ02FFZXQ
Erk_TZ02F9TF8
Eko_XU02J10RR
Erk_TZ02H9HMW
Erk_TZ02FJ0KV
Eko_XU02H85WN
Erk_TZ02IQS4K
Erk_TZ02G96EU
Erk_TZ02JIUHD
Erk_TZ02GMD02
Erk_TZ02JZRUG
Erk_TZ02FHVBE
Erk_TZ02FXG9Y
C07_2236669489
Eko_XU02F2HPM
Vat_XU02GP4FT
Eko_XU02F474V
Eko_XU02JM8B3
DSu_QV02IQ7OH
DSu_QV01AXCPG
DSu_QV02HB9AI
B_C06_2264662947
MSu_QV02I463J
B_L15_2246772312
B_J10_2246771588
MSu_QV02HAB9L
MSu_QV02JKYRN
DSu_QV01EGMAD
C_M09_2236659635
DSu_QV01AMAHH
DSu_QV01DCCXG
DSu_QV01CXGZV
DSu_QV02F70BX
DSu_QV01EQ0WZ
DSu_QV01C41J4
C_P20_2246770264
DSu_QV02GCXJK
DSu_QV01C4SUA
DSu_QV01AVD58
0.02

468 abcT2

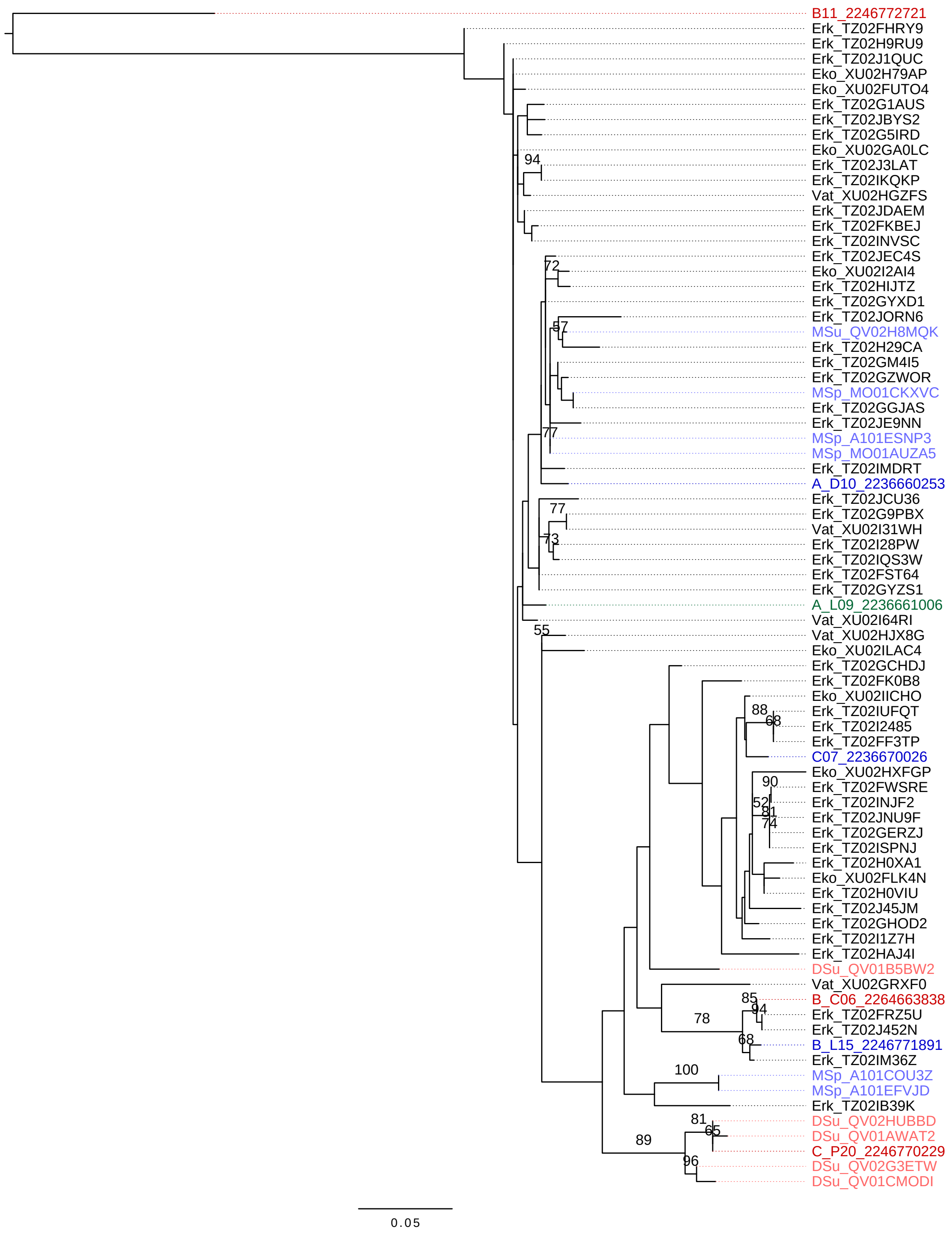

B11_2246772721
Erk_TZ02FHRY9
Erk_TZ02H9RU9
Erk_TZ02J1QUC
Eko_XU02H79AP
Eko_XU02FUTO4
Erk_TZ02G1AUS
Erk_TZ02JBYS2
Erk_TZ02G5IRD
Eko_XU02GA0LC
Erk_TZ02J3LAT
Erk_TZ02IKQKP
Vat_XU02HGZFS
Erk_TZ02JDAEM
Erk_TZ02FKBEJ
Erk_TZ02INVSC
Erk_TZ02JEC4S
Eko_XU02I2AI4
Erk_TZ02HIJTZ
Erk_TZ02GYXD1
Erk_TZ02JORN6
MSu_QV02H8MQK
Erk_TZ02H29CA
Erk_TZ02GM4I5
Erk_TZ02GZWOR
MSp_MO01CKXVC
Erk_TZ02GGJAS
Erk_TZ02JE9NN
MSp_A101ESNP3
MSp_MO01AUZA5
Erk_TZ02IMDRT
A_D10_2236660253
Erk_TZ02JCU36
Erk_TZ02G9PBX
Vat_XU02I31WH
Erk_TZ02I28PW
Erk_TZ02IQS3W
Erk_TZ02FST64
Erk_TZ02GYZS1
A_L09_2236661006
Vat_XU02I64RI
Vat_XU02HJX8G
Eko_XU02ILAC4
Erk_TZ02GCHDJ
Erk_TZ02FK0B8
Eko_XU02IICHO
Erk_TZ02IUFQT
Erk_TZ02I2485
Erk_TZ02FF3TP
C07_2236670026
Eko_XU02HXFGP
Erk_TZ02FWSRE
Erk_TZ02INJF2
Erk_TZ02JNU9F
Erk_TZ02GERZJ
Erk_TZ02ISPNJ
Erk_TZ02H0XA1
Eko_XU02FLK4N
Erk_TZ02H0VIU
Erk_TZ02J45JM
Erk_TZ02GHOD2
Erk_TZ02I1Z7H
Erk_TZ02HAJ4I
DSu_QV01B5BW2
Vat_XU02GRXF0
B_C06_2264663838
Erk_TZ02FRZ5U
Erk_TZ02J452N
B_L15_2246771891
Erk_TZ02IM36Z
MSp_A101COU3Z
MSp_A101EFVJD
Erk_TZ02IB39K
DSu_QV02HUBBD
DSu_QV01AWAT2
C_P20_2246770229
DSu_QV02G3ETW
DSu_QV01CMODI
94
72
67
77
77
73
55
88
68
90
52
81
74
85
94
78
68
100
81
65
89
96
0.05

485 purA

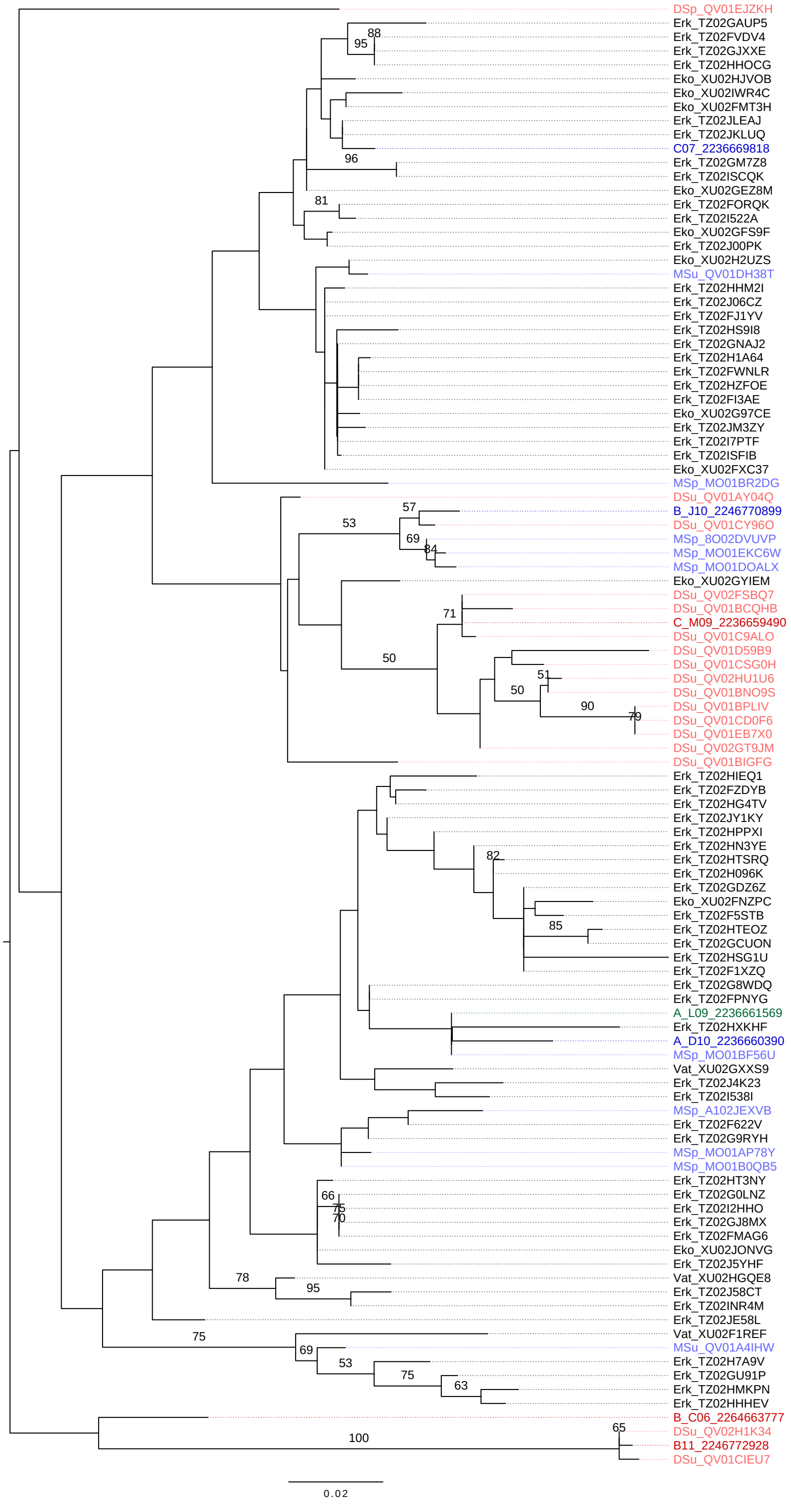

DSp_QV01EJZKH
Erk_TZ02GAUP5
Erk_TZ02FVDV4
Erk_TZ02GJXXE
Erk_TZ02HHOCG
Eko_XU02HJVOB
Eko_XU02IWR4C
Eko_XU02FMT3H
Erk_TZ02JLEAJ
Erk_TZ02JKLUQ
C07_2236669818
Erk_TZ02GM7Z8
Erk_TZ02ISCQK
Eko_XU02GEZ8M
Erk_TZ02FORQK
Erk_TZ02I522A
Eko_XU02GFS9F
Erk_TZ02J00PK
Eko_XU02H2UZS
MSu_QV01DH38T
Erk_TZ02HHM2I
Erk_TZ02J06CZ
Erk_TZ02FJ1YV
Erk_TZ02HS9I8
Erk_TZ02GNAJ2
Erk_TZ02H1A64
Erk_TZ02FWNLR
Erk_TZ02HZFOE
Erk_TZ02FI3AE
Eko_XU02G97CE
Erk_TZ02JM3ZY
Erk_TZ02I7PTF
Erk_TZ02ISFIB
Eko_XU02FXC37
MSp_MO01BR2DG
DSu_QV01AY04Q
B_J10_2246770899
DSu_QV01CY96O
MSp_8O02DVUVP
MSp_MO01EKC6W
MSp_MO01DOALX
Eko_XU02GYIEM
DSu_QV02FSBQ7
DSu_QV01BCQHB
C_M09_2236659490
DSu_QV01C9ALO
DSu_QV01D59B9
DSu_QV01CSG0H
DSu_QV02HU1U6
DSu_QV01BNO9S
DSu_QV01BPLIV
DSu_QV01CD0F6
DSu_QV01EB7X0
DSu_QV02GT9JM
DSu_QV01BIGFG
Erk_TZ02HIEQ1
Erk_TZ02FZDYB
Erk_TZ02HG4TV
Erk_TZ02JY1KY
Erk_TZ02HPPXI
Erk_TZ02HN3YE
Erk_TZ02HTSRQ
Erk_TZ02H096K
Erk_TZ02GDZ6Z
Eko_XU02FNZPC
Erk_TZ02F5STB
Erk_TZ02HTEOZ
Erk_TZ02GCUON
Erk_TZ02HSG1U
Erk_TZ02F1XZQ
Erk_TZ02G8WDQ
Erk_TZ02FPNYG
A_L09_2236661569
Erk_TZ02HXKHF
A_D10_2236660390
MSp_MO01BF56U
Vat_XU02GXXS9
Erk_TZ02J4K23
Erk_TZ02I538I
MSp_A102JEXVB
Erk_TZ02F622V
Erk_TZ02G9RYH
MSp_MO01AP78Y
MSp_MO01B0QB5
Erk_TZ02HT3NY
Erk_TZ02G0LNZ
Erk_TZ02I2HHO
Erk_TZ02GJ8MX
Erk_TZ02FMAG6
Eko_XU02JONVG
Erk_TZ02J5YHF
Vat_XU02HGQE8
Erk_TZ02J58CT
Erk_TZ02INR4M
Erk_TZ02JE58L
Vat_XU02F1REF
MSu_QV01A4IHW
Erk_TZ02H7A9V
Erk_TZ02GU91P
Erk_TZ02HMKPN
Erk_TZ02HHHEV
B_C06_2264663777
DSu_QV02H1K34
B11_2246772928
DSu_QV01CIEU7
0.02

509 acsA

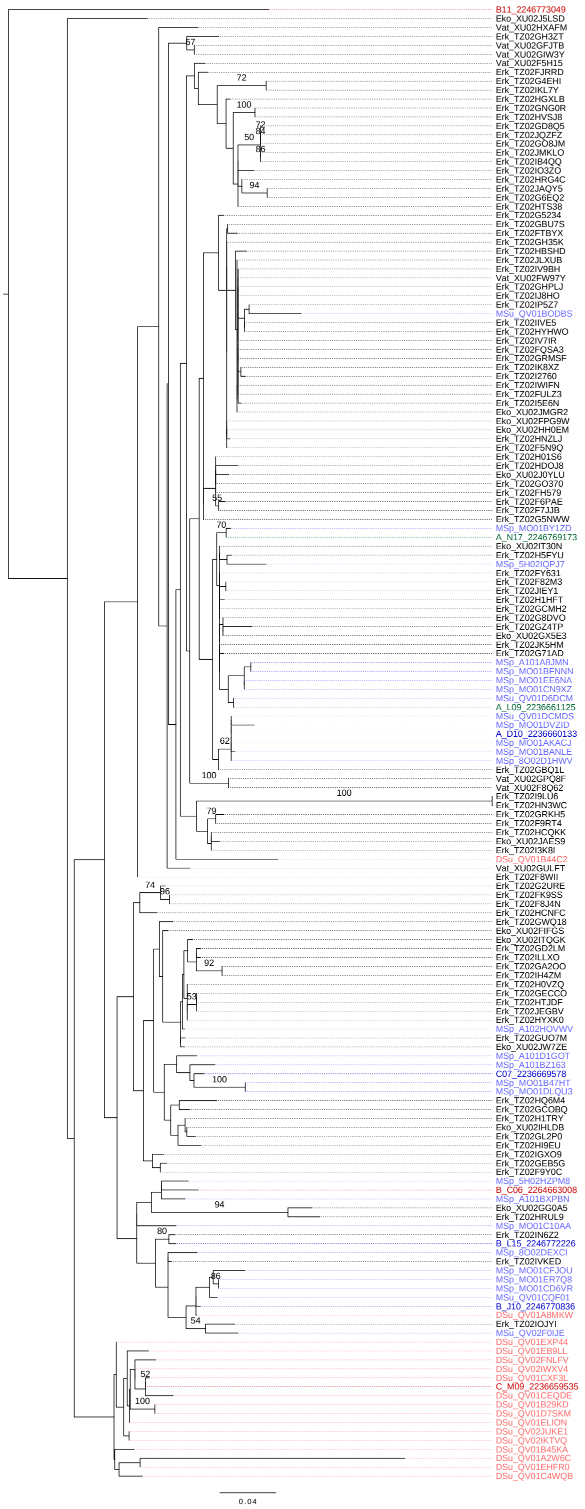

B11_2246773049
Eko_XU02J5LSD
Vat_XU02HXAFM
Erk_TZ02GH3ZT
Vat_XU02GFJTB
Vat_XU02GIW3Y
Vat_XU02F5H15
Erk_TZ02FJRRD
Erk_TZ02G4EHI
Erk_TZ02IKL7Y
Erk_TZ02HGXLB
Erk_TZ02GNG0R
Erk_TZ02HVSJ8
Erk_TZ02GD8Q5
Erk_TZ02JQZFZ
Erk_TZ02GO8JM
Erk_TZ02JMKLO
Erk_TZ02IB4QQ
Erk_TZ02IO3ZO
Erk_TZ02HRG4C
Erk_TZ02JAQY5
Erk_TZ02G6EQ2
Erk_TZ02HTS38
Erk_TZ02G5234
Erk_TZ02GBU7S
Erk_TZ02FTBYX
Erk_TZ02GH35K
Erk_TZ02HBSHD
Erk_TZ02JLXUB
Erk_TZ02IV9BH
Vat_XU02FW97Y
Erk_TZ02GHPLJ
Erk_TZ02IJ8HO
Erk_TZ02IP5Z7
MSu_QV01BODBS
Erk_TZ02IIVE5
Erk_TZ02HYHWO
Erk_TZ02IV7IR
Erk_TZ02FQSA3
Erk_TZ02GRMSF
Erk_TZ02IK8XZ
Erk_TZ02I2760
Erk_TZ02IWIFN
Erk_TZ02FULZ3
Erk_TZ02I5E6N
Eko_XU02JMGR2
Eko_XU02FPG9W
Eko_XU02HH0EM
Erk_TZ02HNZLJ
Erk_TZ02F5N9Q
Erk_TZ02H01S6
Erk_TZ02HDOJ8
Eko_XU02J0YLU
Erk_TZ02GO370
Erk_TZ02FH579
Erk_TZ02F6PAE
Erk_TZ02F7JJB
Erk_TZ02G5NWW
MSp_MO01BY1ZD
A_N17_2246769173
Eko_XU02IT30N
Erk_TZ02H5FYU
MSp_5H02IQPJ7
Erk_TZ02FY631
Erk_TZ02F82M3
Erk_TZ02JIEY1
Erk_TZ02H1HFT
Erk_TZ02GCMH2
Erk_TZ02G8DVO
Erk_TZ02GZ4TP
Eko_XU02GX5E3
Erk_TZ02JK5HM
Erk_TZ02G71AD
MSp_A101A8JMN
MSp_MO01BFNNN
MSp_MO01EE6NA
MSp_MO01CN9XZ
MSu_QV01D6DCM
A_L09_2236661125
MSu_QV01DCMDS
MSp_MO01DVZID
A_D10_2236660133
MSp_MO01AKACJ
MSp_MO01BANLE
MSp_8O02D1HWV
Erk_TZ02GBQ1L
Vat_XU02GPQ8F
Vat_XU02F8Q62
Erk_TZ02I9LU6
Erk_TZ02HN3WC
Erk_TZ02GRKH5
Erk_TZ02F9RT4
Erk_TZ02HCQKK
Eko_XU02JAES9
Erk_TZ02I3K8I
DSu_QV01B44C2
Vat_XU02GULFT
Erk_TZ02F8WII
Erk_TZ02G2URE
Erk_TZ02FK9SS
Erk_TZ02F8J4N
Erk_TZ02HCNFC
Erk_TZ02GWQ18
Eko_XU02FIFGS
Eko_XU02ITQGK
Erk_TZ02GD2LM
Erk_TZ02ILLXO
Erk_TZ02GA2OO
Erk_TZ02IH4ZM
Erk_TZ02H0VZQ
Erk_TZ02GECCO
Erk_TZ02HTJDF
Erk_TZ02JEGBV
Erk_TZ02HYXK0
MSp_A102HOVWV
Erk_TZ02GUO7M
Eko_XU02JW7ZE
MSp_A101D1GOT
MSp_A101BZ163
C07_2236669578
MSp_MO01B47HT
MSp_MO01DLQU3
Erk_TZ02HQ6M4
Erk_TZ02GCOBQ
Erk_TZ02H1TRY
Eko_XU02IHLDB
Erk_TZ02GL2P0
Erk_TZ02HI9EU
Erk_TZ02IGXO9
Erk_TZ02GEB5G
Erk_TZ02F9Y0C
MSp_5H02HZPM8
B_C06_2264663008
MSp_A101BXPBN
Eko_XU02GG0A5
Erk_TZ02HRUL9
MSp_MO01C10AA
Erk_TZ02IN6Z2
B_L15_2246772226
MSp_8O02DEXCI
Erk_TZ02IVKED
MSp_MO01CFJOU
MSp_MO01ER7Q8
MSp_MO01CD6VR
MSu_QV01CQF01
B_J10_2246770836
DSu_QV01A8MKW
Erk_TZ02IOJYI
MSu_QV02F0IJE
DSu_QV01EXP44
DSu_QV01EB9LL
DSu_QV02FNLFV
DSu_QV02IWXV4
DSu_QV01CXF3L
C_M09_2236659535
DSu_QV01CEQDE
DSu_QV01B29KD
DSu_QV01D7SKM
DSu_QV01ELION
DSu_QV02JUKE1
DSu_QV02IKTVQ
DSu_QV01B45KA
DSu_QV01A2W6C
DSu_QV01EHFR0
DSu_QV01C4WQB
0.04

515 pmbA

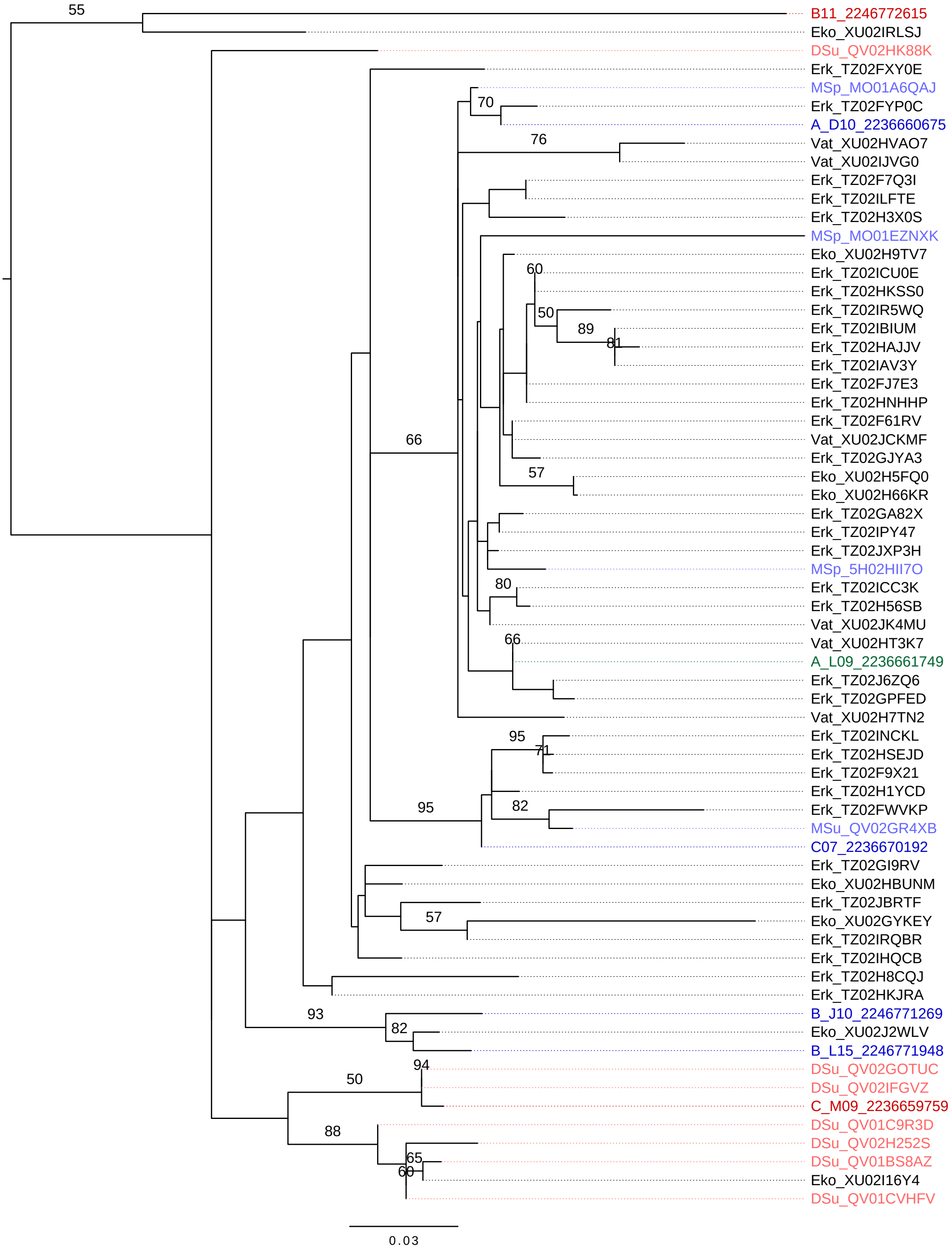
B11_2246772615
Eko_XU02IRLSJ
DSu_QV02HK88K
Erk_TZ02FXY0E
MSp_MO01A6QAJ
Erk_TZ02FYP0C
A_D10_2236660675
Vat_XU02HVAO7
Vat_XU02IJVG0
Erk_TZ02F7Q3I
Erk_TZ02ILFTE
Erk_TZ02H3X0S
MSp_MO01EZNXK
Eko_XU02H9TV7
Erk_TZ02ICU0E
Erk_TZ02HKSS0
Erk_TZ02IR5WQ
Erk_TZ02IBIUM
Erk_TZ02HAJJV
Erk_TZ02IAV3Y
Erk_TZ02FJ7E3
Erk_TZ02HNHHP
Erk_TZ02F61RV
Vat_XU02JCKMF
Erk_TZ02GJYA3
Eko_XU02H5FQ0
Eko_XU02H66KR
Erk_TZ02GA82X
Erk_TZ02IPY47
Erk_TZ02JXP3H
MSp_5H02HII7O
Erk_TZ02ICC3K
Erk_TZ02H56SB
Vat_XU02JK4MU
Vat_XU02HT3K7
A_L09_2236661749
Erk_TZ02J6ZQ6
Erk_TZ02GPFED
Vat_XU02H7TN2
Erk_TZ02INCKL
Erk_TZ02HSEJD
Erk_TZ02F9X21
Erk_TZ02H1YCD
Erk_TZ02FWVKP
MSu_QV02GR4XB
C07_2236670192
Erk_TZ02GI9RV
Eko_XU02HBUNM
Erk_TZ02JBRTF
Eko_XU02GYKEY
Erk_TZ02IRQBR
Erk_TZ02IHQCB
Erk_TZ02H8CQJ
Erk_TZ02HKJRA
B_J10_2246771269
Eko_XU02J2WLV
B_L15_2246771948
DSu_QV02GOTUC
DSu_QV02IFGVZ
C_M09_2236659759
DSu_QV01C9R3D
DSu_QV02H252S
DSu_QV01BS8AZ
Eko_XU02I16Y4
DSu_QV01CVHFV
55
70
76
60
50
89
81
66
57
80
66
95
71
95
82
57
93
82
94
50
88
65
60
0.03

556 M dacF

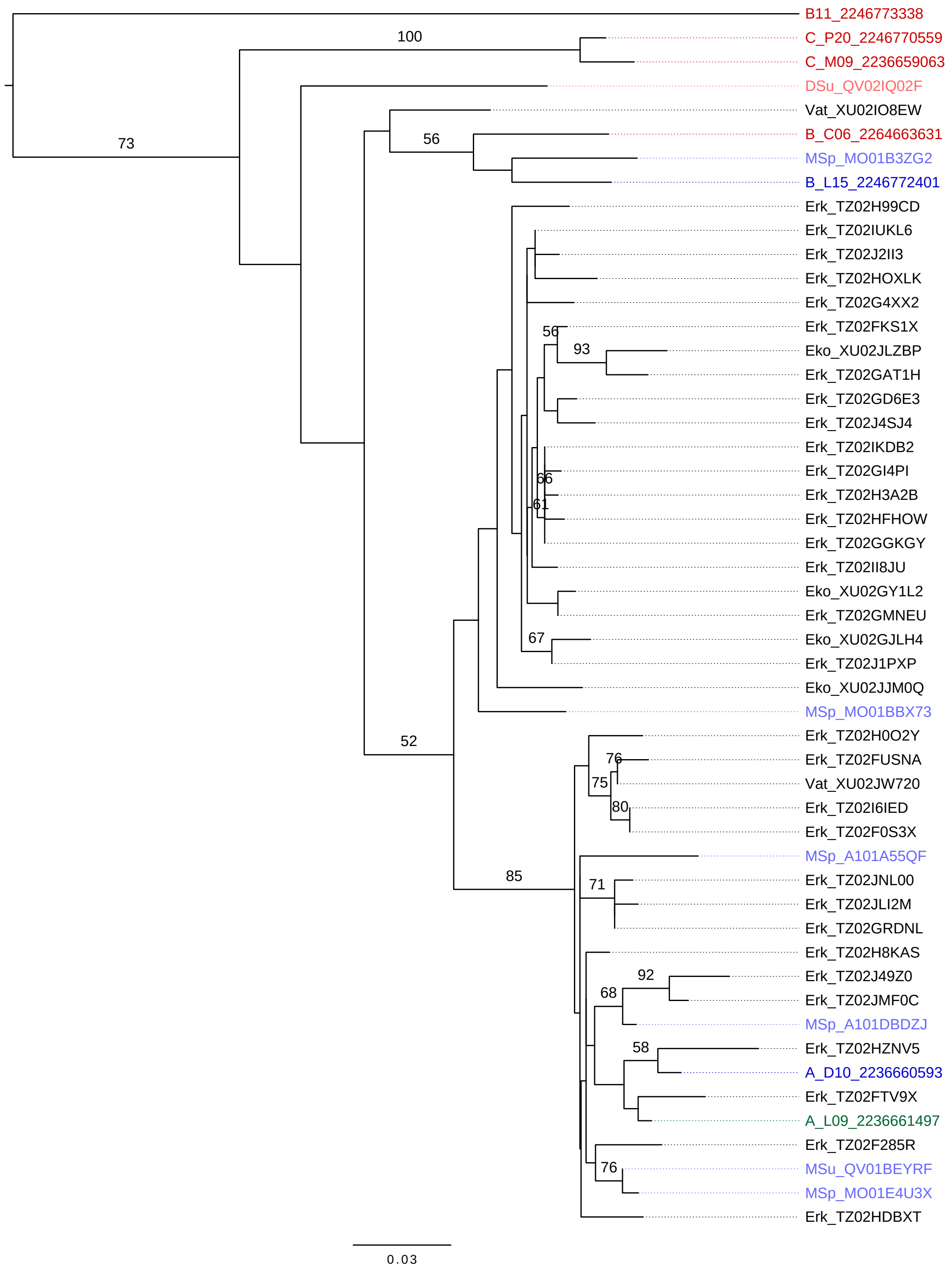
B11_2246773338
C_P20_2246770559
C_M09_2236659063
DSu_QV02IQ02F
Vat_XU02IO8EW
B_C06_2264663631
MSp_MO01B3ZG2
B_L15_2246772401
Erk_TZ02H99CD
Erk_TZ02IUKL6
Erk_TZ02J2II3
Erk_TZ02HOXLK
Erk_TZ02G4XX2
Erk_TZ02FKS1X
Eko_XU02JLZBP
Erk_TZ02GAT1H
Erk_TZ02GD6E3
Erk_TZ02J4SJ4
Erk_TZ02IKDB2
Erk_TZ02GI4PI
Erk_TZ02H3A2B
Erk_TZ02HFHOW
Erk_TZ02GGKGY
Erk_TZ02II8JU
Eko_XU02GY1L2
Erk_TZ02GMNEU
Eko_XU02GJLH4
Erk_TZ02J1PXP
Eko_XU02JJM0Q
MSp_MO01BBX73
Erk_TZ02H0O2Y
Erk_TZ02FUSNA
Vat_XU02JW720
Erk_TZ02I6IED
Erk_TZ02F0S3X
MSp_A101A55QF
Erk_TZ02JNL00
Erk_TZ02JLI2M
Erk_TZ02GRDNL
Erk_TZ02H8KAS
Erk_TZ02J49Z0
Erk_TZ02JMF0C
MSp_A101DBDZJ
Erk_TZ02HZNV5
A_D10_2236660593
Erk_TZ02FTV9X
A_L09_2236661497
Erk_TZ02F285R
MSu_QV01BEYRF
MSp_MO01E4U3X
Erk_TZ02HDBXT
0.03

588 argG

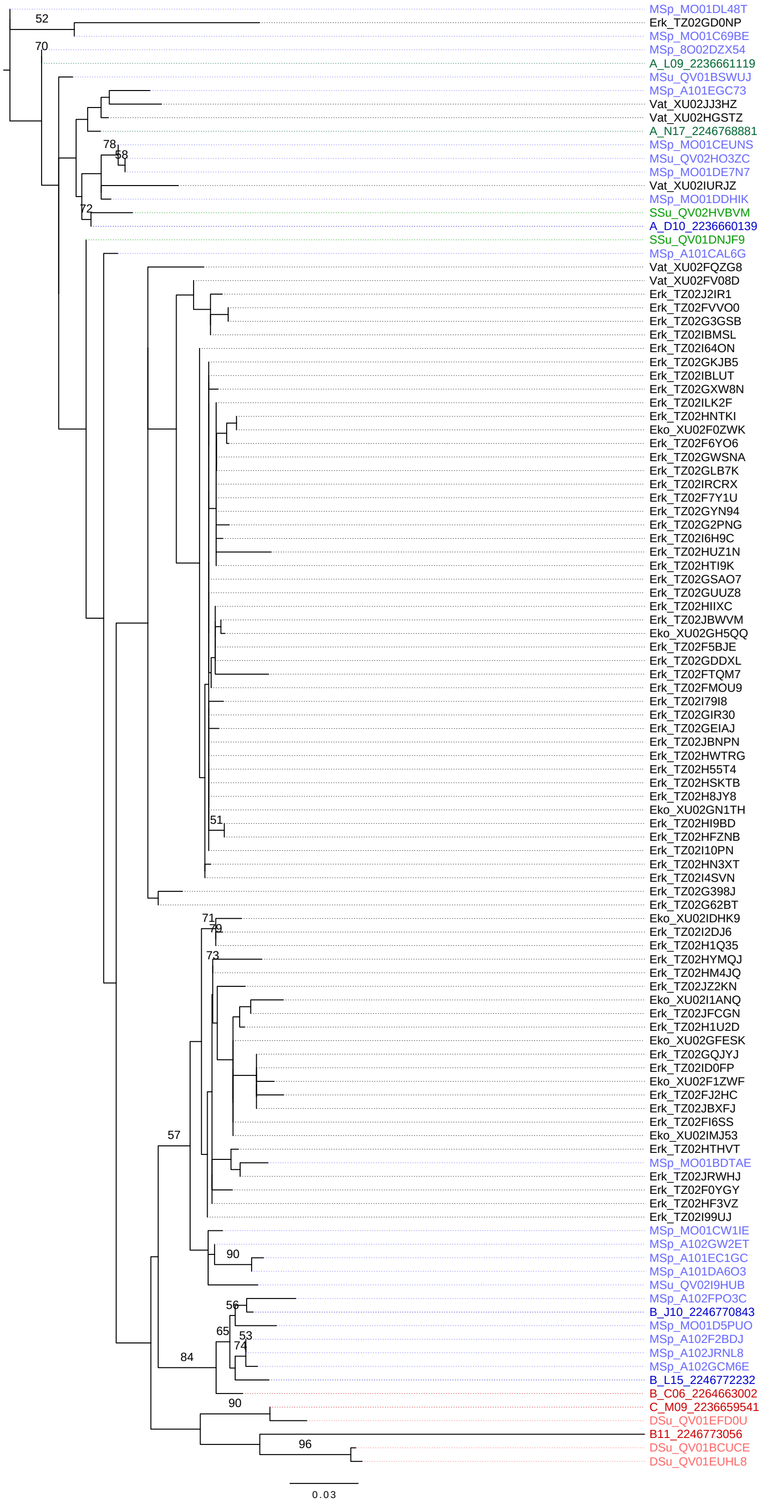
MSp_MO01DL48T
Erk_TZ02GD0NP
MSp_MO01C69BE
MSp_8O02DZX54
A_L09_2236661119
MSu_QV01BSWUJ
MSp_A101EGC73
Vat_XU02JJ3HZ
Vat_XU02HGSTZ
A_N17_2246768881
MSp_MO01CEUNS
MSu_QV02HO3ZC
MSp_MO01DE7N7
Vat_XU02IURJZ
MSp_MO01DDHIK
SSu_QV02HVBVM
A_D10_2236660139
SSu_QV01DNJF9
MSp_A101CAL6G
Vat_XU02FQZG8
Vat_XU02FV08D
Erk_TZ02J2IR1
Erk_TZ02FVVO0
Erk_TZ02G3GSB
Erk_TZ02IBMSL
Erk_TZ02I64ON
Erk_TZ02GKJB5
Erk_TZ02IBLUT
Erk_TZ02GXW8N
Erk_TZ02ILK2F
Erk_TZ02HNTKI
Eko_XU02F0ZWK
Erk_TZ02F6YO6
Erk_TZ02GWSNA
Erk_TZ02GLB7K
Erk_TZ02IRCRX
Erk_TZ02F7Y1U
Erk_TZ02GYN94
Erk_TZ02G2PNG
Erk_TZ02I6H9C
Erk_TZ02HUZ1N
Erk_TZ02HTI9K
Erk_TZ02GSAO7
Erk_TZ02GUUZ8
Erk_TZ02HIIXC
Erk_TZ02JBWVM
Eko_XU02GH5QQ
Erk_TZ02F5BJE
Erk_TZ02GDDXL
Erk_TZ02FTQM7
Erk_TZ02FMOU9
Erk_TZ02I79I8
Erk_TZ02GIR30
Erk_TZ02GEIAJ
Erk_TZ02JBNPN
Erk_TZ02HWTRG
Erk_TZ02H55T4
Erk_TZ02HSKTB
Erk_TZ02H8JY8
Eko_XU02GN1TH
Erk_TZ02HI9BD
Erk_TZ02HFZNB
Erk_TZ02I10PN
Erk_TZ02HN3XT
Erk_TZ02I4SVN
Erk_TZ02G398J
Erk_TZ02G62BT
Eko_XU02IDHK9
Erk_TZ02I2DJ6
Erk_TZ02H1Q35
Erk_TZ02HYMQJ
Erk_TZ02HM4JQ
Erk_TZ02JZ2KN
Eko_XU02I1ANQ
Erk_TZ02JFCGN
Erk_TZ02H1U2D
Eko_XU02GFESK
Erk_TZ02GQJYJ
Erk_TZ02ID0FP
Eko_XU02F1ZWF
Erk_TZ02FJ2HC
Erk_TZ02JBXFJ
Erk_TZ02FI6SS
Eko_XU02IMJ53
Erk_TZ02HTHVT
MSp_MO01BDTAE
Erk_TZ02JRWHJ
Erk_TZ02F0YGY
Erk_TZ02HF3VZ
Erk_TZ02I99UJ
MSp_MO01CW1IE
MSp_A102GW2ET
MSp_A101EC1GC
MSp_A101DA6O3
MSu_QV02I9HUB
MSp_A102FPO3C
B_J10_2246770843
MSp_MO01D5PUO
MSp_A102F2BDJ
MSp_A102JRNL8
MSp_A102GCM6E
B_L15_2246772232
B_C06_2264663002
C_M09_2236659541
DSu_QV01EFD0U
B11_2246773056
DSu_QV01BCUCE
DSu_QV01EUHL8
0.03

694 mutY

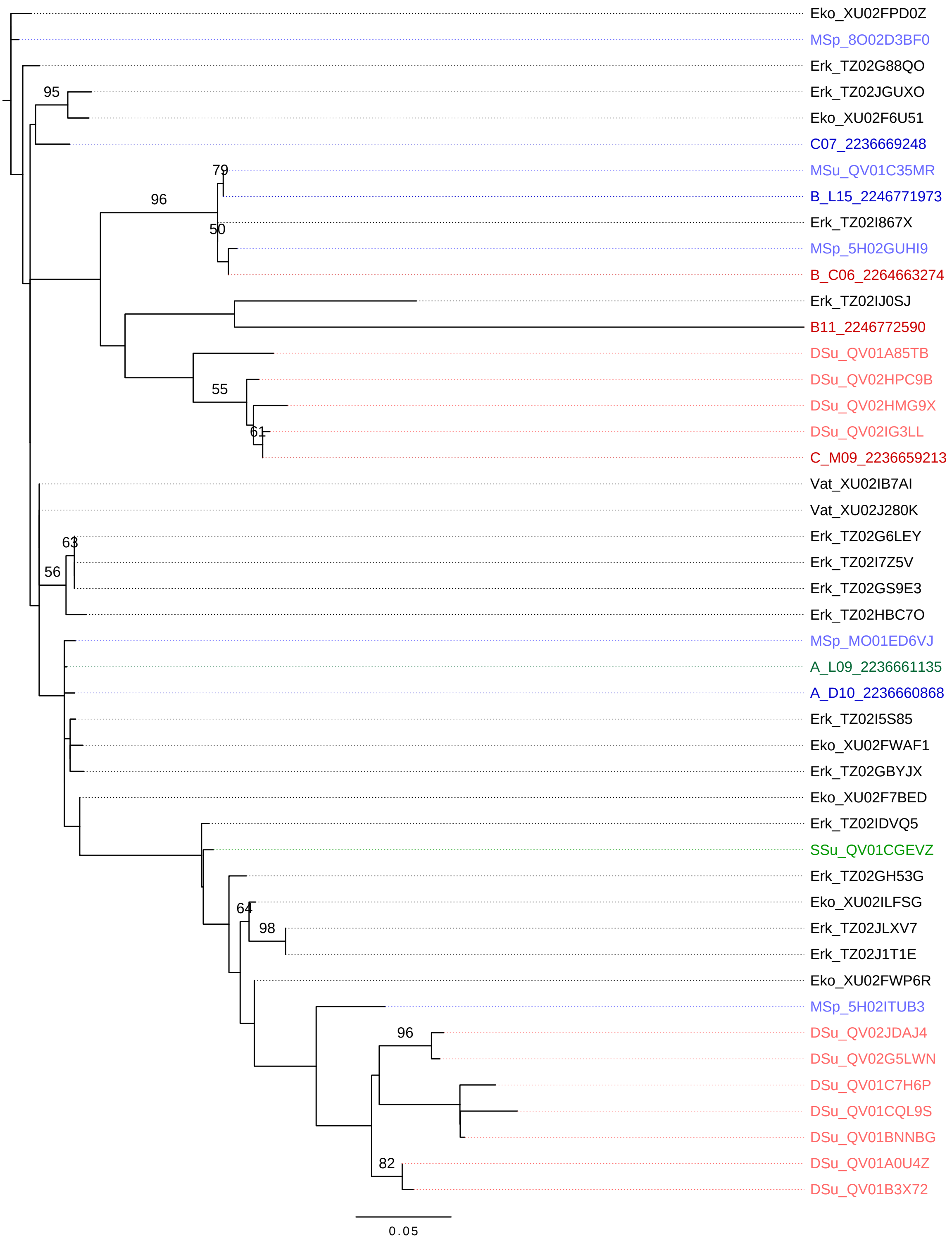
Eko_XU02FPD0Z
MSp_8O02D3BF0
Erk_TZ02G88QO
95
Erk_TZ02JGUXO
Eko_XU02F6U51
C07_2236669248
79
MSu_QV01C35MR
96
B_L15_2246771973
Erk_TZ02I867X
50
MSp_5H02GUHI9
B_C06_2264663274
Erk_TZ02IJ0SJ
B11_2246772590
DSu_QV01A85TB
DSu_QV02HPC9B
55
DSu_QV02HMG9X
61
DSu_QV02IG3LL
C_M09_2236659213
Vat_XU02IB7AI
Vat_XU02J280K
63
Erk_TZ02G6LEY
56
Erk_TZ02I7Z5V
Erk_TZ02GS9E3
Erk_TZ02HBC7O
MSp_MO01ED6VJ
A_L09_2236661135
A_D10_2236660868
Erk_TZ02I5S85
Eko_XU02FWAF1
Erk_TZ02GBYJX
Eko_XU02F7BED
Erk_TZ02IDVQ5
SSu_QV01CGEVZ
Erk_TZ02GH53G
64
Eko_XU02ILFSG
98
Erk_TZ02JLXV7
Erk_TZ02J1T1E
Eko_XU02FWP6R
MSp_5H02ITUB3
96
DSu_QV02JDAJ4
DSu_QV02G5LWN
DSu_QV01C7H6P
DSu_QV01CQL9S
DSu_QV01BNNBG
82
DSu_QV01A0U4Z
DSu_QV01B3X72
0.05
